# Class Ib Ribonucleotide Reductases: Activation of a Peroxido-Mn^II^Mn^III^ to Generate a Reactive Oxo-Mn^III^Mn^IV^ Oxidant

**DOI:** 10.1021/acs.inorgchem.3c04163

**Published:** 2024-01-17

**Authors:** Lorna Doyle, Adriana Magherusan, Shuangning Xu, Kayleigh Murphy, Erik R. Farquhar, Florian Molton, Carole Duboc, Lawrence Que, Aidan R. McDonald

**Affiliations:** † School of Chemistry, Trinity College Dublin, The University of Dublin, College Green, Dublin 2, Ireland; ‡ Department of Chemistry and Centre for Metals in Biocatalysis, University of Minnesota, 207 Pleasant Street SE, Minneapolis, Minnesota 55455, United States; § Case Western Reserve University Center for Synchrotron Biosciences, National Synchrotron Light Source II, Brookhaven National Laboratory Upton, New York 11973, United States; ∥ CNRS UMR 5250, DCM, Univ. Grenoble Alpes, Grenoble F-38000, France

## Abstract

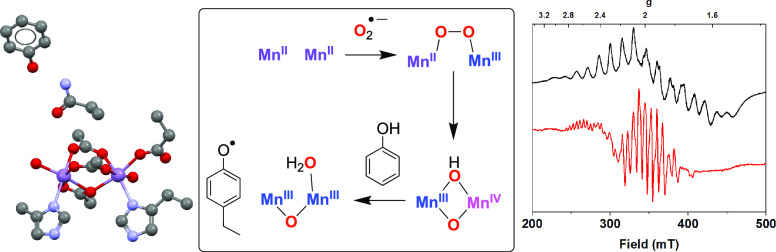

In the postulated catalytic cycle of class Ib Mn_2_ ribonucleotide reductases (RNRs), a Mn^II^_2_ core is suggested to react with superoxide (O_2_^·–^) to generate peroxido-Mn^II^Mn^III^ and oxo-Mn^III^Mn^IV^ entities prior to proton-coupled electron transfer (PCET) oxidation of tyrosine. There is limited experimental support for this mechanism. We demonstrate that [Mn^II^_2_(BPMP)(OAc)_2_](ClO_4_) (**1**, HBPMP = 2,6-bis[(bis(2 pyridylmethyl)amino)methyl]-4-methylphenol) was converted to peroxido-Mn^II^Mn^III^ (**2**) in the presence of superoxide anion that converted to (μ-O)(μ-OH)Mn^III^Mn^IV^ (**3**) via the addition of an H^+^-donor (*p-*TsOH) or (μ-O)_2_Mn^III^Mn^IV^ (**4**) upon warming to room temperature. The physical properties of **3** and **4** were probed using UV–vis, EPR, X-ray absorption, and IR spectroscopies and mass spectrometry. Compounds **3** and **4** were capable of phenol oxidation to yield a phenoxyl radical via a concerted PCET oxidation, supporting the proposed mechanism of tyrosyl radical cofactor generation in RNRs. The synthetic models demonstrate that the postulated O_2_/Mn_2_/tyrosine activation mechanism in class Ib Mn_2_ RNRs is plausible and provides spectral insights into intermediates currently elusive in the native enzyme.

## Abstract 2

The conversion of a peroxido-Mn^II^Mn^III^ complex to an oxo-Mn^III^Mn^IV^ species was achieved, yielding an entity capable of activating phenolic O−H bonds, providing experimental support for the postulated tyrosyl cofactor generation in class Ib Mn_2_ ribonucleotide reductases.

## Introduction

Ribonucleotide reductases (RNRs) are responsible for catalyzing the conversion of ribonucleotides to the corresponding deoxyribonucleotide, providing precursors for deoxyribonucleic acid (DNA) synthesis and repair in all organisms.^1−4^ RNRs are an important target for anticancer drugs, and therefore understanding the enzyme’s mechanism is important.^5^ Each of the three classes of RNRs (I, II, and III) initiates ribonucleotide reduction through the generation of a thiyl radical, which initiates the reductive dehydration of nucleotides. The thiyl radical generation method differs across the three classes in the nature of the metallo-cofactor that facilitates thiyl radical formation.^1−4^ The class I metallo-cofactors contain a dinuclear transition-metal cluster that activates dioxygen to initiate radical production. Classes II and III employ adenosylcobalamin and a 4Fe–4S cluster, respectively, for thiyl radical generation. Class I is further divided into subclasses (a, b, c, and d) depending on the identity of the metallo-cofactor:^1−4^ an Fe_2_ cluster (class Ia), a Mn_2_ cluster (Ib, Id), or an FeMn cluster (Ic).

**Scheme 1:**
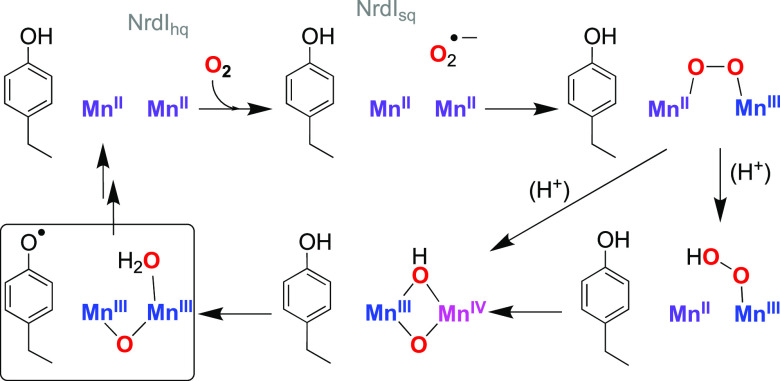
Proposed Catalytic Cycle for Tyrosyl Radical Cofactor Generation in Class Ib RNRs; NrdI_hq_ = Flavodoxin-Like Protein Hydroquinone Cofactor; NrdI_sq_ = Semiquinone Cofactor; Presence of H in the Hydroxide Bridge Has Not Yet Been Experimentally Verified

The mechanism of activation of O_2_ in class Ia RNRs is well-established:^6^ a Fe^II^_2_ core reacts with O_2_ to yield a μ-peroxido-Fe^III^Fe^III^ entity, followed by electron donation and O–O bond scission to yield a bis-μ-oxo-Fe^III^Fe^IV^ oxidant that activates a tyrosine group to yield a tyrosyl radical and Fe^III^_2_. The tyrosyl radical is responsible for initiating the thiyl radical formation. Class Ib RNRs,^7−9^ in contrast, have a considerably less well-understood mode of O_2_ activation (Scheme 1). The Mn_2_ cluster does not react with O_2_: superoxide anion (O_2_^·–^) is postulated to be generated upon the activation of O_2_ by a nearby flavodoxin protein (NrdI-hydroquinone (hq) to semiquinone (sq) conversion), followed by the reaction of O_2_^·–^ with the Mn^II^_2_ core.^10,11^ This is proposed to yield a mixed-valent peroxido-Mn^II^Mn^III^ adduct, which subsequently undergoes O–O bond cleavage to generate bis-μ-oxo-Mn^III^Mn^IV^ species. This Mn^III^Mn^IV^ unit is postulated to be the active oxidant responsible for the oxidation of tyrosine to yield a tyrosyl radical, which then initiates thiyl radical formation. While there is strong evidence of O_2_^·–^ generation during the first step of the process, there is little support for the identity of the products of the reaction between the Mn^II^_2_ core and O_2_^·–^.^6^ The formation of a transient Mn^III^Mn^IV^ entity is supported by a characteristic 16-line electron paramagnetic resonance (EPR) signal,^11^ although little more is known of this species. Furthermore, the role of a proton donor in O–O bond cleavage is poorly understood, and the protonation state of the bis-μ-oxo-Mn^III^Mn^IV^ core is unknown. Another area of uncertainty is the mechanism of the tyrosyl radical generation by the putative bis-μ-oxo-Mn^III^Mn^IV^ species.^12^ This proton-coupled electron transfer (PCET) reaction could proceed in a single concerted step or a stepwise process, whereby electron transfer (ET) is followed by proton transfer (PT) or vice versa.^13^

**Scheme 2:**
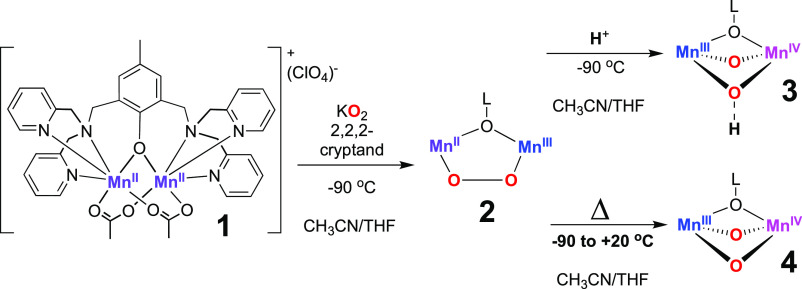
Reaction of **1** with KO_2_ to Yield **2** and the Activation of **2** Using Acids (Top, **2** to **3** Conversion) or via Thermal Decay (Bottom, **2** to **4** Conversion); L–O = BPMP

We explored the properties of synthetic Mn_2_ model complexes in order to understand the postulated mechanism of class Ib RNRs and investigate the structural and electronic properties of the intermediates (Scheme 1). We found that [(Mn^II^_2_)(HPTB)(OAc)](ClO_4_)_2_ (HPTB = *N*,*N*,*N*′,*N*′-tetrakis(2-(benzimidazolyl))-2-hydroxy-1,3-diaminopropane; OAc = acetate) and [Mn^II^_2_(BPMP)(OAc)_2_](ClO_4_), (**1**, Scheme 2, HBPMP = 2,6-bis[(bis(2 pyridylmethyl)amino)methyl]-4-methylphenol) reacted with O_2_^·–^ (KO_2_ solubilized with 18-crown-6) to form peroxido-Mn^II^Mn^III^ species.^14−16^ These studies verified the postulated reactivity of a Mn^II^_2_ core in class Ib RNRs with O_2_·^–^ and were the first examples of mixed-valent peroxido-Mn^II^Mn^III^ complexes. As these peroxide species were relatively inert and unable to activate the weak O–H bonds in phenols, we postulated that such species were not directly responsible for the oxidation of tyrosine in class Ib RNRs. Herein, we describe the activation of a peroxido-Mn^II^Mn^III^ complex to yield Mn^III^Mn^IV^ adducts that were characterized by spectroscopic and spectrometric analyses and for which their PCET phenol oxidation has been explored.

## Results and Discussion

**Figure 1:**
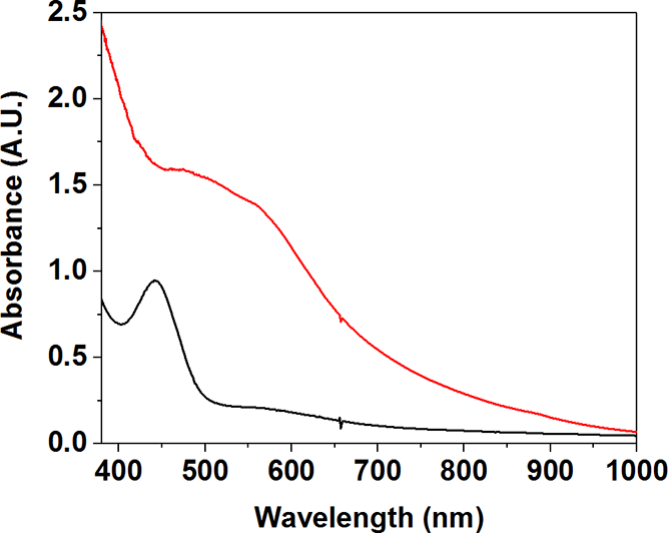
Electronic absorption spectra of **2** (black) and **3** (red trace, formed from the reaction between **2** and *p*-TsOH (2 equiv)).

Compound **1** was prepared according to previous reports.^14−16^ [Mn^II^Mn^III^(O_2_)(BPMP)]^2+^ (**2**) was prepared using a modified procedure (Scheme 1): **1** (1.5 mM) was reacted with KO_2_ (1 equiv) in 1:9 CH_3_CN/tetrahydrofuran (THF) at −90 °C to yield **2**. However, the previously used 18-crown-6 (used to solubilize KO_2_) was replaced with 2,2,2-cryptand (4,7,13,16,21,24-hexaoxa-1,10-diazabicyclo[8.8.8]hexacosane), allowing a notable increase in yield of **2** (Figure S1; see Supporting Information for details). The substitution of 2,2,2-cryptand for 18-crown-6 did not have any effect on the spectroscopic properties of **2**, as evidenced by the same electronic absorption features (λ = 440, 590 nm, Figures 1 and S1). In the same vein, the EPR spectrum of **2** prepared with 2,2,2-cryptand displayed the same 22-line EPR signal centered at *g* = 2, observed when 18-crown-6 was used (Figure S2).

### Acid-Mediated Activation of **2**

Treatment of **2** in 1:9 CH_3_CN/THF (1.5 mM at −90 °C) with *para*-toluenesulfonic acid (*p*-TsOH) resulted in the disappearance of the features assigned to **2**, followed by the immediate formation of a broad electronic absorption feature assigned to a new species (designated as **3**, Figure 1). The maximum yield of **3** was achieved upon the addition of 2 equiv of *p*-TsOH according to electronic absorption and EPR spectroscopies (Figures S3 and S4). It should be noted that treatment of **3** with more than 2 equiv of TsOH or indeed with stronger acids (HCl, HClO_4_, and HBF_4_) resulted in spectral features that can be assigned to a [Mn^II^Mn^III^(BPMP)]^4+^ adduct, comparable to that reported by Hendrikson and co-workers.^17^ We surmise that a large excess of acid or stronger acids caused the release of H_2_O_2_ in those instances. Compound **3** displayed a broad band with λ_max_ ∼ 550 and 590 nm that trailed into the near-infrared (near-IR) region. Complex **3** was found to be metastable, with a half-life *t*_1/2_ = 1200 s at −90 °C. The electronic absorption features of **3** were red-shifted and more intense in comparison to those of the peroxido-Mn^II^Mn^III^ complex **2**. Bis-(μ-O)Mn^III^Mn^IV^ complexes have been found to display similar features. For such complexes, absorption bands typically fall between 520 and 570 nm (assigned to d-to-d transitions) and 590–700 nm (assigned to ligand-to-metal charge transfer (LMCT), e.g., oxo-to-Mn, Table 1).^18−25^ For example, [Mn^III^Mn^IV^(μ-O_2_)(κ^4^-N4py)_2_]^3+^ displayed an intense feature at λ = 565 nm and a less intense one at λ = 667 nm.^25^ The μ-O-Mn^III^Mn^IV^ species, supported by a phenolate ligand, exhibited a broad absorption band at λ_max_ = 570 nm that was attributed to phenolate-to-Mn charge transfer (with oxo-to-Mn at higher energy).^26,27^ Therefore, we concluded that **3** plausibly represents a μ-oxo-Mn_2_ complex based on its electronic absorption features which are consistent with oxo- and phenolate-to-Mn^IV^ transitions.

**Table 1:** Electronic Absorption and Electron Paramagnetic Resonance Properties of Mn^III^Mn^IV^ Complexes

complex	λ (nm)	*g*-value	# of EPR lines (total spectral width [mT])	ref
**3**	550, 590	2	16 (125)	this work
**4**	broad 400–800	2	16 (110)	this work
Ib RNR	broad 300–350	2	16 (130)	(11)
[Mn^III^Mn^IV^(μ-O_2_)(κ^4^-N4py)_2_]^3+^	565, 667	2	16 (140)	(25)
[Mn^III^Mn^IV^(μ-O)_2_(terpy)_2_(OH_2_)_2_]^3+^	553, 654			(23)
[Mn^III^Mn^IV^(μ-O)_2_ (bisimMe_2_en)_2_]^3+^	419, 536, 644	2	16 (135)	(21)
[Mn^III^Mn^IV^ (μ-O)_2_(bpy)_4_]^3+^	525, 555, 684	2	16 (—)	(18)
[Mn^III^Mn^IV^(μ-O)_2_(14-aneN_4_)_2_]^3+^	556, 646	2	16 (—)	(19)
[Mn^III^Mn^IV^(μ-O)_2_(μ-O_2_CMe)(L^M^_2_)]^2+^	259, 400, 440, 548, 640, 800	2	16 (135)	(20)
[Mn^III^Mn^IV^(2-OH)(3,5-Cl_2_Sal)pn)_2_]^+^	400	2	16 (160)	(22)
[Mn^III^Mn^IV^(μ-O)_2_(tmpa)_2_]^3+^	443, 561, 658	2	16 (150)	(28)
[Mn^III^Mn^IV^(μ-O)(L^H^_2_)]^3+^	344, 408, 570	2	18 (130)	(26)

**Figure 2:**
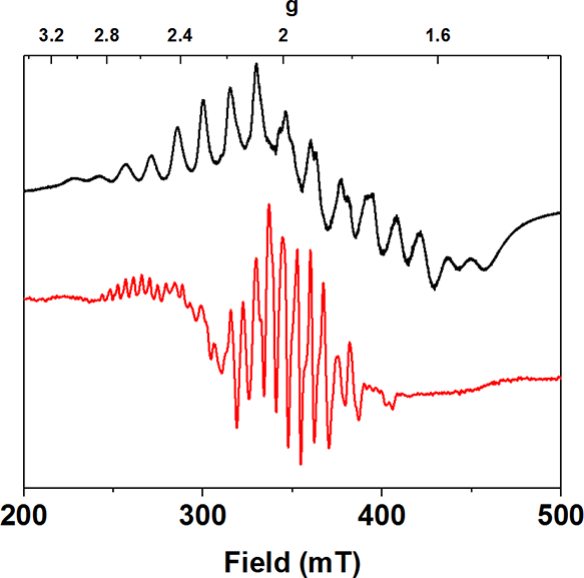
Perpendicular-mode X-band EPR spectra of **2** (black trace) measured at 2 K and of **3** (red trace) measured at 30 K (9.65 GHz, 0.2 mW microwave power).

EPR spectra were recorded on frozen CH_3_CN/THF solutions of **3** (Figures 2 and S5). They displayed a 16-line signal centered at *g* = 2. The average hyperfine coupling constant (*A*_av_) was measured as *A*_av_ = 7.4 mT. Both the number of lines and the *A*_av_ value were consistent with the synthetic Mn^III^Mn^IV^ species and, importantly, Mn^III^Mn^IV^ identified in the studies on class Ib RNRs (Table 1).^29−31^ This is in contrast to the larger value of *A*_av_ = 12.4 mT measured for **2**. Furthermore, the spectral width for the 16-line *g* = 2 signal of **3** was approximately 125 mT, consistent with that measured for the previously described Mn^III^Mn^IV^ complexes.^32,33^ In contrast, the spectral width for **2** spans 250 mT. Dismukes and co-workers postulated that the narrow spectral width allowed Mn^III^Mn^IV^ to be distinguished from Mn^II^Mn^III^ systems.^32^ This disparity in spectral width comes from the spin-coupling coefficients that multiply the hyperfine coupling constants (hfc’s) that are larger for the Mn^II^Mn^III^ complexes than for the Mn^III^Mn^IV^ ones.^34^ Indeed, the individual ion hfc’s for Mn^*n*+^ vary little with *n* for ligands with similar covalency. Moreover, the 16-line *g* = 2 signal showed no temperature dependence, displaying well-resolved signals up to 77 K (Figure S14).^20,35,36^ In contrast, the EPR spectra of Mn^II^Mn^III^ complexes generally need to be recorded below 10 K to be well-resolved. Therefore, we assigned the 16-line *g* = 2 signal of **3** to a Mn^III^Mn^IV^ complex. The yield of the 16-line species was determined by EPR integration to be ∼50 ± 20% (using a Cu^II^ salt as a reference).

A second low-intensity signal was present in the EPR spectrum of **3**: an 11-line feature centered at *g* = 2.6 with an *A*_av_ value of 4.4 mT, corresponding to approximately half of that determined for **3** (7.8 mT). Such a hyperfine pattern is consistent with a complex containing two equivalent Mn (^Mn^I = 5/2) centers. Interestingly, the previously characterized [Mn^II^_2_(TMEDA)_2_(OCCH_3_)_4_(H_2_O)] and [Mn^II^_2_(Me_3_TACN)_2_(μ-OCCH_3_)_3_]BPh_4_ complexes also displayed an 11-line signal centered at *g* = 2.34 with *A*_av_ = 4.5 mT,^37^ and *g* = 1.96 with *A*_av_ = 4.6 mT,^38^ respectively. Therefore, this 11-line EPR feature can be assigned to a Mn^II^_2_ species that differs from **1**. The EPR properties of **1** have been previously investigated by the Hendrickson group and our group, and no such signal has been observed (by us or Hendrickson).^17^ Variable-temperature EPR experiments recorded on peroxide complex **2**, however, revealed the presence of the 11-line EPR signal but in negligible amounts (Figure S6). Therefore, we postulate that upon the addition of superoxide to **1** to yield **2**, a residual Mn^II^_2_ formed in low yield, which remains in solution upon the acid-mediated conversion of **2** to **3**.

Electrospray ionization mass spectrometry (ESI-MS) analysis of a just-thawed methanol solution of **3** revealed a peak at *m*/*Z* = 335.5837, consistent with the mass of the dication [(Mn)_2_(O)_2_(BPMP)]^2+^ (Figure S7; expected mass *m*/*Z* = 335.5682). Other peaks in the mass spectra can be assigned to the free ligand, cryptand, and starting material **1**. The *m*/*Z* = 335 peak was not observed in the ESI-MS spectrum of the peroxide precursor **2**. In the ESI-MS spectrum of **2**, the *m*/*Z* = 770.29 monocation corresponding to [[(Mn)_2_(O)_2_(BPMP)](ClO_4_)]^+^ was observed, which was greatly diminished in the spectrum of **3** (Figure S8). This suggested that the peroxide O–O bond in **2** was cleaved and yielded two oxo-ligands. Preparation of **2** using K^18^O_2_, followed by activation with *p*-TsOH to yield the isotopically labeled form of complex **3**, caused the mass peak to shift by two mass units (*m*/*Z* = 337.5788), consistent with the incorporation of two ^18^O atoms in this dicationic ion, consistent with **3** containing two O atoms in a bis-μ-oxo core derived from KO_2_.

Mn K-edge X-ray absorption near-edge spectroscopy (XANES) was performed on **2** and **3** in order to gain an understanding of the oxidation states of the metal centers and their local geometry (Figure S9). The XANES spectra of the peroxido-Mn^II^Mn^III^**2** displayed a rising edge with the first inflection point calculated to be at 6548.9 eV, consistent with the previously recorded data on the same complex.^14−16^ Upon conversion from **2** to **3,** the edge energy shifted minimally (0.5 eV). Upon irradiation of **3**, we noted a color change of the samples (despite best efforts to irradiate fresh spots with every scan). An increase in the oxidation number of Mn by one unit is typically associated with an edge energy shift of 2–4 eV.^39,40^ We attributed the lack of a shift to the following: photoreduction of **3** by incident X-rays during the XAS measurement and a less-than-optimal yield of Mn^III^Mn^IV^ species **3**, only ∼50 ± 20% by EPR integration. We concluded that XAS would not provide fruitful insights into the electronic and geometric structures of **3**, and thus, we did not pursue further EXAFS measurements.

### Thermal Activation of **2**

**Figure 3:**
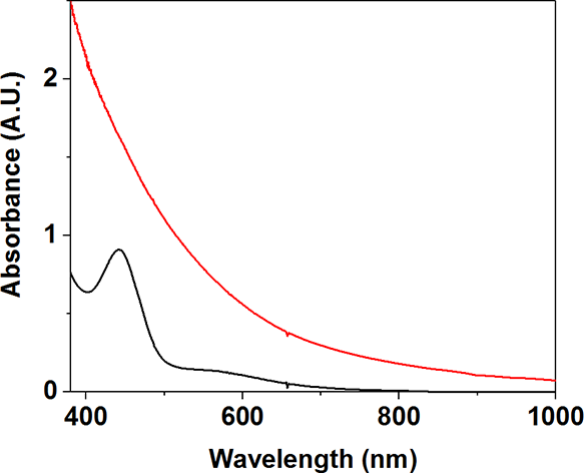
Electronic absorption features for the conversion of **2** (black trace) to **4** (red trace) through thermal activation (−90 to +20 °C) in 1:9 CH_3_CN/THF.

A solution of **2** in CH_3_CN/THF (1:9, 1.5 mM) was warmed from −90 to +20 °C, a process that lasted 900 s (Scheme 2). This resulted in the disappearance of the electronic absorption features assigned to **2**, to yield a broad indistinct absorption that trailed from the UV into the near-IR region, associated with the formation of a new species (defined as **4**, Figure 3). Compound **4** displayed a half-life of *t*_1/2_ = 2300 s at 20 °C.

**Figure 4:**
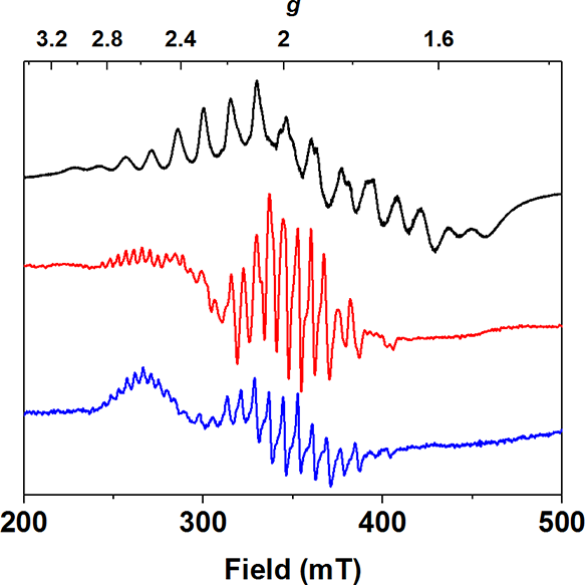
EPR spectra of **2** (black trace) recorded at 2 K, **3** (red trace) at 30 K, and **4** (blue trace) at 7 K (9.65 GHz, 0.2 mW microwave power).

EPR analysis of **4** measured at 2 K revealed two new signals, a 16-line signal centered at *g* = 2 with *A*_av_ = 7.9 mT and a 11-line signal centered at *g* = 2.6 with *A*_av_ = 4.4 mT (Figures 4 and S10). The spectrum obtained was remarkably similar to that measured for **3**. For the same reasons outlined above, we assigned the 16-line *g* = 2.0 signal observed for **4** to a Mn^III^Mn^IV^ species. The quantification of the 16-line EPR spectrum indicates a conversion from **3** to **4** in ∼60% ± 20% yield. As in the case of **3**, we postulated that the *g* = 2.6 signal originates from a Mn^II^_2_ species. The EPR spectra of **3** and **4** display similar signals at *g* = 2 regarding their line-shape and the number of lines. The *g* = 2 signals of both **3** and **4** display similar spectral widths of 125 and 110 mT, respectively. Furthermore, both features were resolved at temperatures higher than 10 K (Figure S11). Based on the similarities of the EPR characteristics of complexes **3** and **4**, we postulated that they are both Mn^III^Mn^IV^ species, with a bis-μ-oxo Mn^III^Mn^IV^ core for **4** and a μ-oxo-μ-hydroxo-Mn^III^Mn^IV^ core for **3**. We ascribe a difference in the protonation states in **3** and **4** based on the analysis of their absorption spectra and on the reactivity studies described below.

ESI-MS of a just-thawed solution of **4** revealed a peak at *m*/*Z* = 335.58 that corresponded to the dication [Mn_2_(O)_2_(BPMP)]^2+^ (Figure S12). Preparation of **4** using K^18^O_2_ caused this peak to shift by 2 mass units, (*m*/*Z* = 337.5788) consistent with two ^18^O oxygen atoms in the oxo moiety in the dicationic molecule. As with **3**, this is indicative of the formation of a Mn^III^Mn^IV^ species with a bis(μ-oxo) core structure. Both complexes **3** and **4** displayed the same dication peaks in their mass spectra, indicating that both complexes form similar species during the activation of **2**.

The broad, tailing UV–vis spectrum observed for **4** has previously been observed for Mn^III^Mn^IV^ complexes (Table 1), which generally display absorbance features in the visible region, typically derived from the ligand-to-metal charge transfer or d–d transitions.^18−25^ For example, the UV–vis spectrum of a putative bis(μ-oxo)Mn^III^Mn^IV^ species in the recently discovered class Id RNRs displayed a similar tailing absorption into the UV region.^41^ Likewise, [bis(μ-oxo)Mn^III^Mn^IV^(2-OH(3,5-Cl_2_Sal)pn)_2_]ClO_4_ showed a broad spectrum that tailed into the UV region.^22^ Therefore, **4** is plausibly a bis(μ-oxo)Mn^III^Mn^IV^ species. Overlay of the electronic absorption spectra of **3** and **4** revealed some similarities (Figure S13). Both **3** and **4** displayed broad absorptions that trail into the near-IR region of the spectra. However, the important difference between the two are the features at λ_max_ = 550, 590 nm, postulated to be oxo- or phenolate-to-Mn^IV^ charge transfer, displayed by **3,** that is not present in the spectrum of **4**. In studies exploring the protonation of the bis(μ-oxo) cores of Mn^IV^_2_ complexes, conducted by Pecoraro, it was discovered that the addition of H^+^ resulted in the shifting of the absorption features attributed to O-to-Mn^IV^ charge transfer.^27^ We surmise that the loss of features in **4** with respect to **3** could be attributed to a similar shifting of charge-transfer bands. This supports the conclusion that a protonation state difference exists in **3** and **4**, suggesting that perhaps **3** contains a protonated core, while **4** contains an unprotonated bis(μ-oxo) core, given that no external protons have been added to the reaction mixture containing **4**.

The addition of a base to **3** allowed us to probe its protonation state further. Upon the addition of triethylamine (NEt_3_, 100 equiv) to **3**, an immediate reaction was observed (Figure S14). The obtained electronic absorption spectrum was similar to the broad, featureless spectrum of **4**. We postulate that a (μ-oxo)(μ-hydroxo) core in **3** was deprotonated by the base NEt_3_ to yield the bis(μ-oxo) complex, **4**. This is consistent with the fact that **3** is generated from an acid-mediated activation, whereas **4** was prepared in the absence of an acid.

Mn K-edge XANES was performed on **4;** however, the results were equally as nonfruitful as those obtained for **3** (Figure S15).

Measurement of the vibrational properties of **3** and **4** by Raman and infrared (IR) spectroscopies provided limited insights. The following limitations of our vibrational analysis were identified: resonances of interest (ν_O–O_, ν_Mn–O_) for **3** and **4** could be masked by the solvent (mixture of CH_3_CN/THF) and/or 2,2,2-cryptand and/or BPMP signals. Furthermore, the thermal instability of compound **3** prevented IR analysis. The IR analysis of **4** showed a new feature at ν_Mn–O_ = 712 cm^–1^ that we tentatively assign to a Mn_2_O_2_ diamond core, the so-called breathing mode (Figure S16). Unfortunately, due to an unexpected explosion of our in-house-prepared K^18^O_2_ (see Supporting Information for details), we were unable to analyze an isotopically labeled sample. The vibrational properties of μ-oxo-Mn_2_ species have been probed through Raman and IR spectroscopies,^42−45^ showing features for ν_Mn–O_ ∼ 690 cm^–1^ assigned to the Mn_2_O_2_ diamond core breathing modes. We tentatively conclude that **4** contains a Mn_2_O_2_ diamond core.

With the combination of electronic absorption, EPR, IR, and mass spectrometry data, we assign the new species **3** to be a (μ-oxo)(μ-hydroxo)Mn^III^Mn^IV^ species and **4** to be a bis(μ-oxo)Mn^III^Mn^IV^ complex formed during the protonation or thermal decay of peroxido-Mn^II^Mn^III^**2**, respectively (Scheme 2). We postulate that the addition of the weak acid *p*-TsOH as a proton source facilitated O–O bond scission in the peroxide complex **2** and promoted conversion to the oxo-bridged Mn^III^Mn^IV^ species. In close analogy to the conversion of **2** to **3**, Que and co-workers recently reported the cleavage of a μ-1,2-peroxido-Fe^III^_2_ species upon the addition of the Lewis acid Sc^3+^ to generate a bis-μ-oxo-Fe^IV^_2_core.^46^ Moreover, with respect to the formation of **4** from **2**, Jackson and co-workers reported a mononuclear peroxido-Mn^III^ species ([Mn^III^(O_2_)(κ^4^-N4Py)]^2+^) that thermally decayed into a bis(μ-oxo)Mn^III^Mn^IV^ species [Mn^III^(μ-O)_2_Mn^IV^(κ^4^-N4py)_2_]^3+^.^25^ Alongside the reactivity studies below, these literature precedents and our collated spectroscopic data support our structural assignments of **3** and **4**.

### Reactivity of **3** with Phenols

For the catalytic cycle of class Ib RNRs, it has been postulated that a bis-μ-oxo-Mn^III^Mn^IV^ species is responsible for the oxidation of a nearby tyrosine residue initiating ribonucleotide reduction.^5^ To explore this reactivity further, Mn^III^Mn^IV^ complexes **3** and **4** were reacted with phenols.

**Figure 5:**
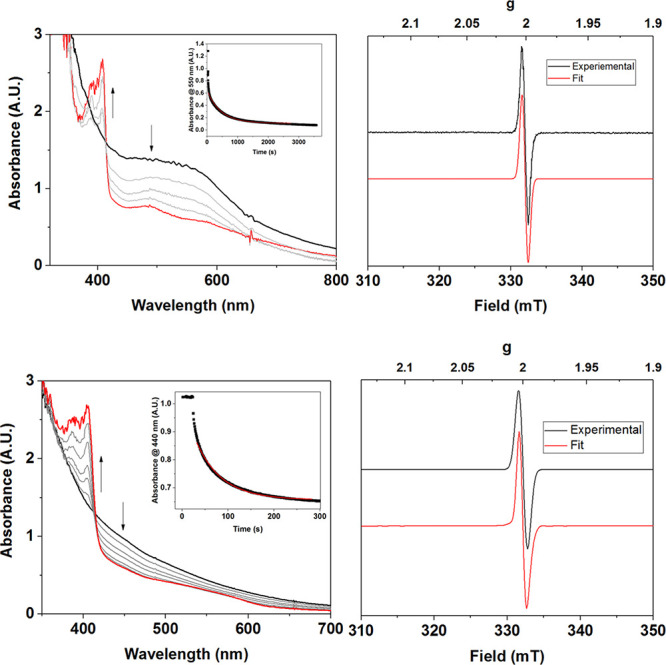
Top left: electronic absorption spectra of the reaction of **3** (black) with 4-CH_3_O-2,6-DTBP (50 equiv; red trace is the spectrum at the end of the reaction). Inset: plot of the change of absorption at λ = 550 nm over time. Top right: Perpendicular-mode X-band EPR spectrum of the reaction of **3** and 4-CH_3_O-2,6-DTBP measured at 77 K (9.65 GHz, 0.2 mW microwave power, black trace) and simulated spectra (red trace). Bottom left: electronic absorption spectra changes in the reaction of **4** with 4-CH_3_O-2,6-DTBP. Inset: plot of the change in absorption at λ = 440 nm over time. Bottom right: perpendicular-mode X-band EPR spectrum of the reaction of **4** with 4-CH_3_O-2,6-DTBP measured at 77 K (9.65 GHz, 0.2 mW microwave power, black trace) and the simulated spectrum (red trace).

The addition of 4-methoxy-2,6-di-*tert*-butylphenol (4-CH_3_O-2,6-DTBP, 50 equiv) to **3** at −90 °C resulted in the decay of the electronic absorption features associated with **3** and the simultaneous growth of a sharp feature at λ_max_ = 405 nm (Figure 5) which matched the features attributed to the independently synthesized 4-methoxy-2,6-di-*tert*-butylphenoxyl radical (Figure S17).^47,48^ The EPR analysis of the postreaction mixture of **3** and 4-CH_3_O-2,6-DTBP revealed a sharp and isotropic signal centered at *g* = 2, indicative of the formation of an organic radical (Figure 5),^49^ with a yield of 35% ± 20%. Interestingly, no other signals (or signals displaying Mn-hyperfine) were observed in the postreaction EPR. This implies the formation of an EPR-silent Mn product following the reaction of **3** with 4-CH_3_O-2,6-DTBP, possibly a Mn^III^Mn^III^ moeity. Compound **3** thus facilitated the PCET oxidation of a phenol to yield a phenoxyl radical, mimicking the proposed tyrosyl radical generation process in class Ib RNRs.

Compound **3** also reacted with 9-azabicyclo[3.3.1]nonane-*N*-hydroxide (ABNO-H) and substrates from the 1-hydroxy-2,2,6,6-tetramethylpiperidine (4-X-TEMPO-H, where X = O, H, and C(O)CH_3_) family, allowing us to gain mechanistic insights (Table 2 and Figures S18–S30). These reactions showed the disappearance of the features associated with **3**, as determined by electronic absorption spectroscopy. EPR analysis of the postreaction mixture of **3** and TEMPO-H displayed an intense rhombic signal with *g*_av_ = 2.00, consistent with the previously reported spectra for the 2,2,6,6-tetramethyl-piperidin-1-yl)oxyl (TEMPO) radical product (Figures S31–S34).^50^ The postreaction mixtures for the **3** + ABNO-H, 4-CH_3_O-TEMPO-H, and 4-oxo-TEMPOH reactions all displayed similar intense rhombic signals with *g*_av_ = 2.00. The yields of the radical species were calculated to be 35–75% (±20%) using EPR spectroscopy, assuming a one-electron oxidation (Table 2). In the ESI-MS spectrum of the postreaction mixture of **3** and TEMPO-H, the mass peak assigned to **3** (*m*/*Z* = 335.5837) was no longer present (Figure S35). This provides further evidence for **3** being the active oxidant responsible for the activation of the O–H bonds. In summary, for a series of substrates containing O–H bonds, **3** facilitated PCET oxidation to yield an organic-oxyl radical product in good- to-high yields.

**Table 2:** Kinetic Parameters, Yields of Products, and Bond Dissociation Free Energy (BDFE) Values for the Reactions of O–H Bond-Containing Substrates with **3** and **4**

	*k*_2_ (M^–1^ s^–1^)	*k*_1_ (s^–1^)	*K*_M_ (M^–1^)	BDFE_O–H_ (kcal mol^–1^)	EPR yield of radical (±20%)
**3**^a^					
[H]-4-CH_3_O-2,6-DTBP	0.0072			74.9	35
[D]-4-CH_3_O-2,6-DTBP	0.0028				
ABNO-H	0.018			71.5	40
TEMPO-H	0.150			66.5	75
4-CH_3_O-TEMPO-H	0.258			65	60
4-oxo-TEMPO-H	0.134			65.6	75
**4**^b^					
4-CN-2,6-DTBP		0.014	0.81	79.4	0
4-H-2,6-DTBP		0.023	0.02	78.3	10
4-CH_3_-2,6-DTBP		0.025	0.12	76.9	0
4-C_2_H_5_-2,6-DTBP		0.058	0.09	75.5	0
2,4,6-TTBP		0.024	0.52	76.7	20
[H]-4-CH_3_O-2,6-DTBP		0.086	0.002	73.8	70
[D]-4-CH_3_O-2,6-DTBP		0.032	0.0006		

a For **3**: measured at −90 °C; the BDFE_O–H_ values of each substrate are reported in CH_3_CN;^51−53^ BDFE_O–H_ values for ABNO-H were not available in CH_3_CN, and hence the value was derived using gas-phase bond dissociation enthalpies. Details in Supporting Information.

b For **4**: measured at +20 °C; the BDFE_O–H_ values for the substrates were not available in 1:9 CH_3_CN/THF; hence, we have reported BDFE_O–H_ values for benzene.

Fitting of the decay of **3** by following the feature at λ = 550 nm after the addition of substrate (>10 equiv to ensure pseudo*-*first-order conditions) yielded a rate of decay (*k*_obs_). A plot of the *k*_obs_ values versus substrate concentration revealed a linear relationship for all substrates, the slope of which was determined to obtain second-order reaction rate constants (*k*_2_). The free energies of activation of the substrates (Δ*G*^‡^) were calculated using the Arrhenius equation, and using these values, a Bell–Evans–Polyani (BEP) plot was constructed (Figure S36). A linear fit of the BEP plot for complex **3** afforded a slope of 0.16, which is within the range (0.15–0.7) predicted by the Marcus theory for concerted PCET mechanisms (thus, hydrogen atom transfer (HAT) or concerted proton and electron transfer (CPET)).^51−53^ Furthermore, a stepwise reaction (thus, PT, followed by ET, or vice versa) with TEMPO-H is thermodynamically unlikely because there is a high barrier to the initial deprotonation or one-electron oxidation of TEMPO-H (p*K*_a_ = 41 and *E*^o^ = 0.71 V vs ferrocene/ferrocenium in CH_3_CN).^51−53^ A kinetic isotope effect (KIE, *k*_H_/*k*_D_) of 2.7 was determined for the reaction of **3** with O–H bonds using H/D-4-CH_3_O-2,6-DTBP (Figure S37). This KIE value falls within the classical range (2–7) and indicates that PT or HAT was rate-limiting and is in agreement with the KIE of 3.5 determined for the similar complex (μ-oxo)(μ-hydroxo)Mn^III^Mn^IV^(L^Mepy^) that performs HAT.^54^ The similarities offer further indication that **3** utilizes a concerted PCET mechanism during the oxidation of O–H bond-containing substrates.

### Reactivity of **4** with Phenols

Addition of 4-CH_3_O-2,6-DTBP to a solution of **4** (1.5 mM, 1:9 CH_3_CN/THF, 10 equiv) resulted in an immediate decrease in the absorption features associated with **4**, concomitant with an increase in the new feature at λ = 405 nm (Figure 5). As described above, this sharp peak is associated with the formation of the 4-methoxy-2,6-di-*tert*-butyl-phenoxyl radical (Figure S38). A frozen EPR sample of the postreaction mixture showed a sharp isotropic signal centered at *g* = 2, confirming the formation of the radical (Figure 5). The yield of 4-methoxy-2,6-di-*tert*-butyl-phenoxyl radical was determined to be 75 ± 20% by EPR. Thus, as with **3**, **4** was capable of PCET oxidation of phenols to yield the phenoxyl radical, also mimicking the postulated tyrosyl radical generation process in class Ib RNRs.

Complex **4** was reacted with a family of phenols 4-X-2,6-DTBP, where X = (CH_3_O, C(CH_3_)_3_, C_2_H_5_, CH_3_, H, and CN) to gain mechanistic insights (Table 2 and Figures S39–S59). The reactions between **4** and ABNO-H or TEMPO-H and its derivatives were too fast for accurate kinetic analysis. This does not suggest that **4** was more potent than **3** because the reactivity difference can more likely be ascribed to the different temperatures at which the reactions were performed (−90 °C for **3** and + 20 °C for **4**). The postreaction mixtures were analyzed via EPR spectroscopy (Figures S60 and S61). 4-CH_3_O-2,6-DTBP, 2,4,6-TTBP, and 2,6-DTBP revealed the formation of the corresponding phenoxyl radical species in 75, 20, and 10% (±20%) yields, respectively. The postreaction mixtures for 4-C_2_H_5_-2,6-DTBP, 4-CH_3_-2,6-DTBP, and 4-CN-2,6-DTBP displayed no EPR signals at 77 K, indicating that any formed phenoxyl radicals had decayed further. Gas chromatography (GC) analysis of the postreaction mixtures for X = CH_3_O, C(CH_3_)_3_, CN, and C_2_H_5_ showed the formation of 2,6-di-*tert*-butyl-1,4-benzoquinone (2,6-DTBQ), a product of the decay of the parent phenoxyl radicals. Interestingly, as with **3**, in the postreaction EPR spectra for all substrates, no Mn signals were observed. Overall, **4** reacted with the phenolic substrates to yield phenoxyl radicals, which then decayed further in some instances.

**Figure 6:**
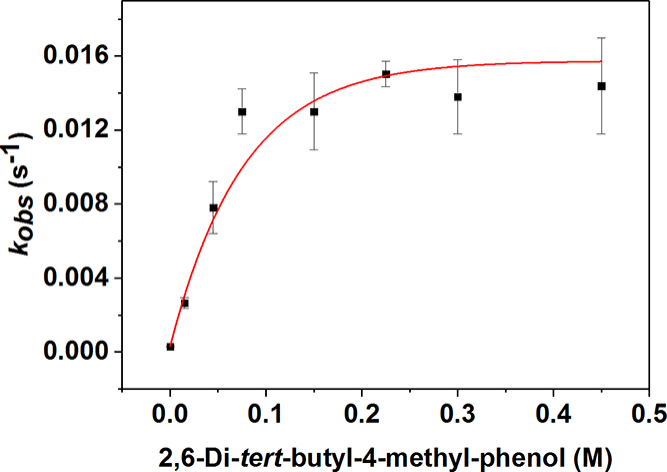
Plot of substrate concentration versus *k*_obs_ for the reaction of **4** and 4-CH_3_-2,6-DTBP.

Plotting the measured *k*_obs_ values against the substrate concentration ([*S*]) for the reaction between **4** and 4-CH_3_-2,6-DTBP revealed a nonlinear behavior (Figure 6), a trend which was observed for the reactions between **4** and all substrates. This trend indicates the presence of an equilibrium prior to an irreversible substrate transformation. In order to gain a deeper understanding of how **4** was reacting, the kinetic plots of the phenols were linearized according to the equation, , allowing us to calculate *k*_1_, the maximum rate of reaction, and *K*_M_, the [*S*] at half the maximum rate. Using *k*_1_, Δ*G*^‡^ was calculated for each substrate. Plotting Δ*G*^‡^ versus BDFE_O–H_ yielded a plot with a slope of 0.15 (Figure S62). As with **3**, **4** falls in the range of slopes reported for systems ascribed to concerted PCET (0.15–0.7),^51−53^ indicating that concerted PCET was the mechanism **4** employed to oxidize phenols. A plot of the logs of *k*_1_ or *K*_M_ versus the p*K*_a_’s, or *K*_M_ versus *E*_OX_ of the substrates revealed no discernible relationship (Figures S63–S65). In contrast, a Marcus plot of (*RT*/*F*) ln(*k*_1_) versus *E*_OX_ was linear with a slope of −0.36 (Figure S66). This value is on the borderline between those associated with the rate-limiting concerted PCET and rate-limiting ET.^55−58^ A KIE of 2.7 was determined for the reaction of **4** with H/D-4-CH_3_O-2,6-DTBP (Figures S67 and S68), emblematic of the PT or HAT involved in the rate-limiting step. This further supports a concerted PCET mechanism during the oxidation of phenols of **4**. Previous studies into the reactions of bis-μ-oxo-Mn_2_ complexes with hydrocarbons and phenols provided similar insights—the oxidants abstracted H atoms via a concerted PCET mechanism.^59,60^ The KIE value was identical to that obtained for **3**, suggesting that both complexes follow a similar pathway. Based on the collected evidence, we tentatively conclude that **3** and **4** oxidized substrates via a concerted PCET reaction mechanism.

**Scheme 3:**
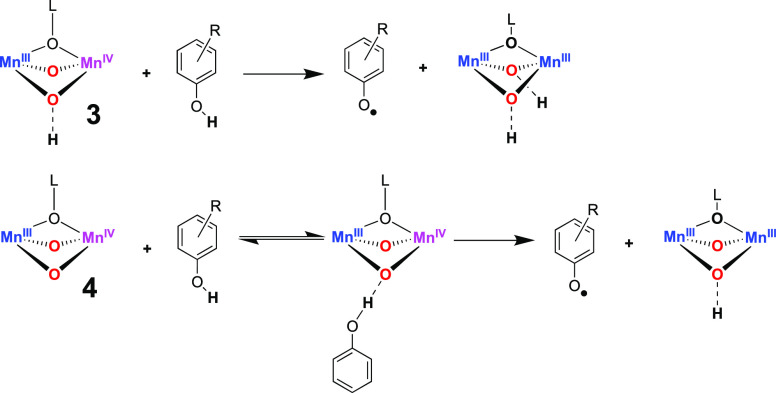
Graphical Explanation of the Influence of Protons in PCET Oxidation by **3** and **4**

We postulate that the source of the nonlinear kinetics in the reactivity of **4** is an initial association of the phenol O–H moiety to the bis-μ-oxo-Mn^III^Mn^IV^ core (Scheme 3). Jackson and co-workers reported Mn^III^–OH supported by the bis-pyridylamine monocarboxamidate ligand dpaq (dpaq = 2-[bis(pyridin-2-ylmethyl)]amino-*N*-quinolin-8-yl-acetamidate) that showed a similar saturation kinetic behavior in its reaction with the same family of phenols.^50^ They postulated that Mn^III^–OH formed a hydrogen-bonded adduct with phenol in the initial step, followed by an irreversible oxidation of the phenol. The structures of **3** and **4** differ only by the availability of protons that could reside on a μ-oxo-ligand in **3**, which are presumed absent in **4** due to the nonavailability of protons in the reaction mixture containing **4**. The absence of saturation kinetics for **3** supports this structural assignment and is consistent with our observations of linear kinetics from **3** in its reactions with the O–H bond-containing substrates.

As stated in Introduction, the role of a H^+^-donor in O–O bond cleavage in RNRs is poorly understood, and the protonation state of the bis-μ-oxo-Mn^III^Mn^IV^ core is unknown. The current evidence demonstrates that an H^+^ can facilitate O–O bond cleavage in a peroxide-Mn^II^Mn^III^ complex to yield a bis-μ-oxo-Mn^III^Mn^IV^ entity, **3**. However, provision of an H^+^ is not a prerequisite for bis-μ-oxo-Mn^III^Mn^IV^ formation, as evidenced by the thermally induced formation of **4**. Second, the protonation state has an impact on the reactivity properties of bis-μ-oxo-Mn^III^Mn^IV^ cores, where the current evidence demonstrates that the phenolic substrate displays a pre-equilibrium hydrogen-bonding step prior to its oxidation, in its interactions with a bis-μ-oxo-Mn^III^Mn^IV^ oxidant.

## Conclusions and Mechanistic Implications for Class Ib RNRs

In the postulated catalytic cycle of class Ib RNRs (Scheme 1), a Mn^II^_2_ center is suggested to react with superoxide (O_2_^–^), rather than dioxygen, to generate an intermediate peroxido-Mn^II^Mn^III^ adduct. The peroxido-Mn^II^Mn^III^ adduct is believed to rapidly convert to a putative Mn^III^Mn^IV^ entity, where the involvement/role of a H^+^-donor is poorly understood. The Mn^III^Mn^IV^ entity, identified through EPR analysis, is understood to be responsible for the tyrosyl radical/Mn^III^_2_ cofactor generation via PCET oxidation of tyrosine. In this work, a Mn^II^_2_ complex (**1**) was reacted with O_2_^–^ to form peroxido-Mn^II^Mn^III^ (**2**). Importantly, **1** did not react with atmospheric O_2_. **2** was converted to (μ-O)(μ-OH)Mn^III^Mn^IV^ (**3**) via the addition of an H^+^-donor (*p-*TsOH) or (μ-O)_2_Mn^III^Mn^IV^ (**4**) upon warming to room temperature. The physical properties of **3** and **4** were probed using UV–vis, EPR, X-ray absorption, and IR spectroscopies and mass spectrometry. Interestingly, **3** and **4** displayed EPR spectra similar to those of the implicated active Mn^III^Mn^IV^ species identified in the studies of class Ib RNRs, where a 16-line signal consistent with a Mn^III^Mn^IV^ oxidant was also observed. The synthetic model thus provides experimental support for the postulated biochemical O_2_ activation and high-valent oxidant generation pathways proposed for class Ib Mn_2_ RNRs. Critically, the trapped synthetic Mn^III^Mn^IV^ complexes **3** and **4** were capable of phenol oxidation to yield a phenoxyl radical, supporting the proposed mechanism of tyrosyl radical/Mn^III^_2_ cofactor generation in RNRs. Kinetic analysis revealed that **3** and **4** were oxidizing phenolic substrates through a concerted PCET reaction mechanism, albeit with different kinetic profiles consistent with their different protonation states. The synthetic models reported herein thus support the entire postulated catalytic cycle of the enzyme and provide insights into the spectral and reactivity properties of the as-yet unidentified intermediates in the RNR cycle.

## Supporting Information Available

Experimental and physical methods, NMR, electronic absorption, EPR, IR, XAS spectra, mass spectrometry data, kinetics data, and analysis (PDF)

## Notes

Perchlorate salts of metal complexes and KO_2_ are potentially explosive and must be handled with care and in small quantities. We limited our preparation of **1** to a maximum of 0.2 g per reaction. **Caution:** preparation and storage of K^18^O_2_: we had a minor explosion, inside our glovebox, when handling (using a metal spatula) a small quantity (20–40 mg) of our in-house-prepared K^18^O_2_ in a glass Schlenk flask. Details are provided in the Supporting Information.

## References

[ref1] Eriksson M.; Jordan A.; Eklund H. Structure of *Salmonella typhimurium nrdF* Ribonucleotide Reductase in Its Oxidized and Reduced Forms. Biochemistry 1998, 37, 13359–13369. 10.1021/bi981380s. 9748343

[ref2] Stubbe J.; Nocera D. G.; Yee C. S.; Chang M. C. Y. Radical Initiation in the Class I Ribonucleotide Reductase: Long-Range Proton-Coupled Electron Transfer?. Chem. Rev. 2003, 103, 2167–2201. 10.1021/cr020421u. 12797828

[ref3] Kolberg M.; Strand K. R.; Graff P.; Andersson K. K. Structure, function, and mechanism of ribonucleotide reductases. Biochim. Biophys. Acta (BBA) - Proteins Proteomics 2004, 1699 (1), 1–34. 10.1016/j.bbapap.2004.02.007. 15158709

[ref4] Cotruvo J. A.; Stubbe J. Class I Ribonucleotide Reductases: Metallocofactor Assembly and Repair In Vitro and In Vivo. Annu. Rev. Biochem. 2011, 80 (1), 733–767. 10.1146/annurev-biochem-061408-095817. 21456967 PMC4703083

[ref5] Aye Y.; Li M.; Long M. J. C.; Weiss R. S. Ribonucleotide reductase and cancer: biological mechanisms and targeted therapies. Oncogene 2015, 34 (16), 2011–2021. 10.1038/onc.2014.155. 24909171

[ref6] Ruskoski T. B.; Boal A. K. The periodic table of ribonucleotide reductases. J. Biol. Chem. 2021, 297 (4), 101137 10.1016/j.jbc.2021.101137. 34461093 PMC8463856

[ref7] Boal A. K.; Cotruvo J. A.; Stubbe J.; Rosenzweig A. C. Structural Basis for Activation of Class Ib Ribonucleotide Reductase. Science 2010, 329 (5998), 1526–1530. 10.1126/science.1190187. 20688982 PMC3020666

[ref8] Cotruvo J. A. Jr.; Stubbe J. Escherichia coli Class Ib Ribonucleotide Reductase Contains a Dimanganese(III)-Tyrosyl Radical Cofactor in Vivo. Biochemistry 2011, 50 (10), 1672–1681. 10.1021/bi101881d. 21250660 PMC3076206

[ref9] Boal A. K.; Cotruvo J. A. Jr; Stubbe J.; Rosenzweig A. C. The Dimanganese(II) Site of Bacillus subtilis Class Ib Ribonucleotide Reductase. Biochemistry 2012, 51 (18), 3861–3871. 10.1021/bi201925t. 22443445 PMC3348363

[ref10] Cox N.; Ogata H.; Stolle P.; Reijerse E.; Auling G.; Lubitz W. A Tyrosyl–Dimanganese Coupled Spin System is the Native Metalloradical Cofactor of the R2F Subunit of the Ribonucleotide Reductase of Corynebacterium ammoniagenes. J. Am. Chem. Soc. 2010, 132 (32), 11197–11213. 10.1021/ja1036995. 20698687

[ref11] Cotruvo J. A. Jr.; Stich T. A.; Britt R. D.; Stubbe J. Mechanism of Assembly of the Dimanganese-Tyrosyl Radical Cofactor of Class Ib Ribonucleotide Reductase: Enzymatic Generation of Superoxide Is Required for Tyrosine Oxidation via a Mn(III)Mn(IV) Intermediate. J. Am. Chem. Soc. 2013, 135 (10), 4027–4039. 10.1021/ja312457t. 23402532 PMC3739481

[ref12] Kang G.; Taguchi A. T.; Stubbe J.; Drennan C. L. Structure of a trapped radical transfer pathway within a ribonucleotide reductase holocomplex. Science 2020, 368 (6489), 424–427. 10.1126/science.aba6794. 32217749 PMC7774503

[ref13] Minnihan E. C.; Nocera D. G.; Stubbe J. Reversible, Long-Range Radical Transfer in E. coli Class Ia Ribonucleotide Reductase. Acc. Chem. Res. 2013, 46 (11), 2524–2535. 10.1021/ar4000407. 23730940 PMC3823682

[ref14] Magherusan A. M.; Nelis D. N.; Twamley B.; McDonald A. R. Catechol oxidase activity of comparable dimanganese and dicopper complexes. Dalton Trans. 2018, 47 (43), 15555–15564. 10.1039/C8DT01378K. 30345446

[ref15] Magherusan A. M.; Zhou A.; Farquhar E. R.; García-Melchor M.; Twamley B.; Que L.; McDonald A. R. Mimicking Class I b Mn_2_Ribonucleotide Reductase: A Mn^II^_2_ Complex and Its Reaction with Superoxide. Angew. Chem., Int. Ed. 2018, 57 (4), 918–922. 10.1002/anie.201709806. PMC5873984 29165865

[ref16] Magherusan A. M.; Kal S.; Nelis D. N.; Doyle L. M.; Farquhar E. R.; Que L.; McDonald A. R. A Mn^II^Mn^III^-Peroxide Complex Capable of Aldehyde Deformylation. Angew. Chem., Int. Ed. 2019, 58 (17), 5718–5722. 10.1002/anie.201900717. PMC6456399 30830996

[ref17] Cooper S. R.; Dismukes G. C.; Klein M. P.; Calvin M. Mixed Valence Interactions in Di-μ-oxo Bridged Manganese Complexes. Electron Paramagnetic Resonance and Magnetic Susceptibility Studies. J. Am. Chem. Soc. 1978, 100 (23), 7248–7252. 10.1021/ja00491a021.

[ref18] Brewer K. J.; Calvin M.; Lumpkin R. S.; Otvos J. W.; Spreer L. O. Synthesis, structure, and characterization of a mixed-valence manganese(III)-manganese(IV) bis-μ-oxo complex with a macrocyclic tetraaza ligand. Inorg. Chem. 1989, 28 (25), 4446–4451. 10.1021/ic00324a007.

[ref19] Mahapatra S.; Das P.; Mukherjee R. A new mixed-valence binuclear complex containing the [Mn^IV^(μ-O)_2_(μ-O_2_CMe)Mn^III^]^2+^ core: synthesis, magnetism, electron paramagnetic resonance and redox properties. J. Chem. Soc., Dalton Trans. 1993, 2, 217–220. 10.1039/DT9930000217.

[ref20] Frapart Y.-M.; Boussae A.; Albach R.; Anxolabéhére-Mallart E.; Delroisse M.; Verlhac J.-B.; Blondin G.; Girerd J.-J.; Guilhem J.; Cesario M.; et al. Chemical Modeling of the Oxygen-Evolving Center in Plants. Synthesis, Structure, and Electronic and Redox Properties of a New Mixed Valence Mn-Oxo Cluster: [Mn_2_^III,IV^O_2_(bisimMe_2_en)_2_]^3+^ (bisimMe_2_en = *N,N’*-Dimethyl-*N,N’*-bis(imidazol-4-ylmethyl)ethane-1,2-diamine). EPR Detection of an Imidazole Radical Induced by UV Irradiation at Low Temperature. J. Am. Chem. Soc. 1996, 118, 2669–2678. 10.1021/ja9436411.

[ref21] Gelasco A.; Kirk M. L.; Kampf J. W.; Pecoraro V. L. The [Mn_2_(2-OHsalpn)_2_]^2-,-,0,+^ System: Synthesis, Structure, Spectroscopy, and Magnetism of the First Structurally Characterized Dinuclear Manganese Series Containing Four Distinct Oxidation States. Inorg. Chem. 1997, 36 (9), 1829–1837. 10.1021/ic970140i. 11669787

[ref22] Collomb M.-N.; Deronzier A.; Richardot A. L.; Pe′caut J. Synthesis and characterization of a new kind of Mn_2_^III,IV^ μ-oxo complex: [Mn_2_O_2_(terpy)_2_(H_2_O)_2_](NO_3_)_3_·6 H_2_O, terpy = 2,2′:6′,2″-terpyridine. New. J. Chem. 1999, 23 (4), 351–354. 10.1039/A900814D.

[ref23] Yamazaki H.; Igarashi S.; Nagata T.; Yagi M. Substituent Effects on Core Structures and Heterogeneous Catalytic Activities of Mn^III^(μ-O)_2_Mn^IV^ Dimers with 2,2′:6′,2″-Terpyridine Derivative Ligands for Water Oxidation. Inorg. Chem. 2012, 51 (3), 1530–1539. 10.1021/ic201797h. 22280017

[ref24] Leto D. F.; Chattopadhyay S.; Day V. W.; Jackson T. A. Reaction landscape of a pentadentate N5-ligated Mn^II^ complex with O_2_^·–^ and H_2_O_2_ includes conversion of a peroxomanganese(III) adduct to a bis(μ-oxo)dimanganese(III,IV) species. Dalton Trans. 2013, 42 (36), 13014–13025. 10.1039/c3dt51277k. 23872704 PMC4498396

[ref25] Horner O.; Anxolabéhère-Mallart E.; Charlot M.-F.; Tchertanov L.; Guilhem J.; Mattioli T. A.; Boussac A.; Girerd J.-J. A New Manganese Dinuclear Complex with Phenolate Ligands and a Single Unsupported Oxo Bridge. Storage of Two Positive Charges within Less than 500 mV. Relevance to Photosynthesis. Inorg. Chem. 1999, 38 (6), 1222–1232. 10.1021/ic980832m. 11670906

[ref26] Baldwin M. J.; Stemmler T. L.; Riggs-Gelasco P. J.; Kirk M. L.; Penner-Hahn J. E.; Pecoraro V. L. Structural and Magnetic Effects of Successive Protonations of Oxo Bridges in High-Valent Manganese Dimers. J. Am. Chem. Soc. 1994, 116, 11349–11356. 10.1021/ja00104a014.

[ref27] Suzuki M.; Tokura S.; Suhara M.; Uehara A. Dinuclear Manganese(III,IV) and Manganese (IV, IV) Complexes with Tris(2-pyridylmethyl)amine. Chem. Lett. 1988, 17, 477–480. 10.1246/cl.1988.477.

[ref28] Goodson P. A.; Hodgson D. J. Synthesis and Characterization of a Bis-Oxo-Bridged Mn^III^Mn^III^ Complex, Di-μ-oxobis[N,N’-bis(2,6-dimethylpyridyl)ethane-1,2-diamine]dimanganese(III,III) Perchlorate. Inorg. Chem. 1989, 28, 3606–3608. 10.1021/ic00317a043.

[ref29] Goodson P. A.; Oki A. R.; Glerup J.; Hodgson D. J. Design, Synthesis, and Characterization of Bis(μ-oxo)dimanganese(III,III) Complexes. Steric and Electronic Control of Redox Potentials. J. Am. Chem. Soc. 1990, 112, 6248–6254. 10.1021/ja00173a011.

[ref30] Goodson P. A.; Glerup J.; Hodgson D. J.; Michelsen K.; Pedersen E. Binuclear bis(μ-oxo)dimanganese(III,IV) and -(IV,IV) complexes with N,N’-bis(2-pyridylmethyl)-1,2-ethanediamine. Inorg. Chem. 1990, 29 (3), 503–508. 10.1021/ic00328a034.

[ref31] Dismukes G. C.; Sheats J. E.; Smegal J. A. Mn^2+^/Mn^3+^ and Mn^3+^/Mn^4+^ Mixed Valence Binuclear Manganese Complexes of Biological Interest. J. Am. Chem. Soc. 1987, 109, 7202–7203. 10.1021/ja00257a057.

[ref32] Messinger J.; Robblee J. H.; Yu W. O.; Sauer K.; Rachandra V. K.; Klein M. P. The S_0_ State of the Oxygen-Evolving Complex in Photosystem II Is Paramagnetic: Detection of an EPR Multiline Signal. J. Am. Chem. Soc. 1997, 119, 11349–11350. 10.1021/ja972696a. 25221336 PMC4161286

[ref33] Teutloff C.; Schäfer K.-O.; Sinnecker S.; Barynin V.; Bittl R.; Wieghardt K.; Lendzian F.; Lubitz W. High-field EPR investigations of Mn^III^Mn^IV^ and Mn^II^Mn^III^ states of dimanganese catalase and related model systems. Magn. Reson. Chem. 2005, 43 (S1), S51–S64. 10.1002/mrc.1685. 16235205

[ref34] Hureau C.; Sabater L.; Anxolabéhère-Mallart E.; Nierlich M.; Charlot M.-F.; Gonnet F.; Rivière E.; Blondin G. Synthesis, Structure, and Characterisation of a New Phenolato-Bridged Manganese Complex [Mn_2_(mL)_2_]^2+^: Chemical and Electrochemical Access to a New Mono-μ-Oxo Dimanganese Core Unit. Chem.—Eur. J. 2004, 10 (8), 1998–2010. 10.1002/chem.200305515. 15079840

[ref35] Lessa J. A.; Horn A. Jr; Bull É. S.; Rocha M. R.; Benassi M.; Catharino R. R.; Eberlin M. N.; Casellato A.; Noble C. J.; Hanson G. R.; et al. Catalase vs Peroxidase Activity of a Manganese(II) Compound: Identification of a Mn(III)–(μ-O)_2_–Mn(IV) Reaction Intermediate by Electrospray Ionization Mass Spectrometry and Electron Paramagnetic Resonance Spectroscopy. Inorg. Chem. 2009, 48 (10), 4569–4579. 10.1021/ic801969c. 19425615

[ref36] Howard T.; Telser J.; DeRose V. J. An Electron Paramagnetic Resonance Study of Mn_2_(H_2_O)(OAc)_4_(tmeda)_2_ (tmeda = N,N,N‘,N‘-Tetramethylethylenediamine): A Model for Dinuclear Manganese Enzyme Active Sites. Inorg. Chem. 2000, 39 (15), 3379–3385. 10.1021/ic0000247. 11196878

[ref37] Golombek A. P.; Hendrich M. P. Quantitative analysis of dinuclear manganese(II) EPR spectra. J. Magn. Reson. 2003, 165 (1), 33–48. 10.1016/j.jmr.2003.07.001. 14568515

[ref38] Diril H.; Chang H.-R.; Nilges M. J.; Zhang X.; Potenza J. A.; Schugar H. J.; Isied S. S.; Hendrickson D. N. Simulation Strategies for Unusual EPR Spectra of Binuclear Mixed-Valence Manganese Complexes: Synthesis, Properties, and X-ray Structures of the Mn^II^Mn^III^ Complexes [Mn_2_(bpmp)(μ-OAc)_2_](ClO_4_)·H_2_O and [Mn_2_(bcmp)(m-OAc)_2_](ClO_4_)·CH_2_Cl_2_. J. Am. Chem. Soc. 1989, 111, 5102–5114. 10.1021/ja00196a013.

[ref39] Stemmler T. L.; Sossong J.; T M.; Goldstein J. I.; Ash D. E.; Elgren T. E.; Kurtz D. M. Jr.; Penner-Hahn J. E. EXAFS Comparison of the Dimanganese Core Structures of Manganese Catalase, Arginase, and Manganese-Substituted Ribonucleotide Reductase and Hemerythrin. Biochemistry 1997, 36, 9847–9858. 10.1021/bi9702795. 9245417

[ref40] Schreiber R. E.; Cohen H.; Leitus G.; Wolf S. G.; Zhou A.; Que L. Jr; Neumann R. Reactivity and O_2_ Formation by Mn(IV)- and Mn(V)-Hydroxo Species Stabilized within a Polyfluoroxometalate Framework. J. Am. Chem. Soc. 2015, 137 (27), 8738–8748. 10.1021/jacs.5b03456. 26070034 PMC4939246

[ref41] Rose H. R.; Ghosh M. K.; Maggiolo A. O.; Pollock C. J.; Blaesi E. J.; Hajj V.; Wei Y.; Rajakovich L. J.; Chang W.-C.; Han Y.; et al. Structural Basis for Superoxide Activation of Flavobacterium johnsoniae Class I Ribonucleotide Reductase and for Radical Initiation by Its Dimanganese Cofactor. Biochemistry 2018, 57 (18), 2679–2693. 10.1021/acs.biochem.8b00247. 29609464 PMC6488936

[ref42] Cua A.; Vrettos J. S.; de Paula J. C.; Brudvig G. W.; Bocian D. F. Raman spectra and normal coordinate analyses of low-frequency vibrations of oxo-bridged manganese complexes. J. Bioinorg. Chem. 2003, 8 (4), 439–451. 10.1007/s00775-002-0433-4. 12761665

[ref43] Cooper S. R.; Calvin M. Mixed valence interactions in di-μ-oxo bridged manganese complexes. J. Am. Chem. Soc. 1977, 99 (20), 6623–6630. 10.1021/ja00462a025.

[ref44] Manchanda R.; Brudvig G. W.; Crabtree R. H. High-Valent Oxomanganese Clusters: Structural and Mechanistic Work Relevant to the Oxygen Evolving Center in Photosystem II. Coord. Chem. Rev. 1995, 144, 1–38. 10.1016/0010-8545(95)01147-H.

[ref45] Dubois L.; Pécaut J.; Charlot M.-F.; Baffert C.; Collomb M.-N.; Deronzier A.; Latour J.-M. Carboxylate Ligands Drastically Enhance the Rates of Oxo Exchange and Hydrogen Peroxide Disproportionation by Oxo Manganese Compounds of Potential Biological Significance. Chem.—Eur. J. 2008, 14 (10), 3013–3025. 10.1002/chem.200701253. 18293345

[ref46] Banerjee S.; Draksharapu A.; Crossland P. M.; Fan R.; Guo Y.; Swart M.; Que L. Jr Sc^3+^-Promoted O–O Bond Cleavage of a (μ-1,2-Peroxo)Diiron(III) Species Formed from an Iron(II) Precursor and O_2_ to Generate a Complex with an Fe^IV^_2_(μ-O)_2_ Core. J. Am. Chem. Soc. 2020, 142 (9), 4285–4297. 10.1021/jacs.9b12081. 32017545 PMC7136037

[ref47] Wittman J. M.; Hayoun R.; Kaminsky W.; Coggins M. K.; Mayer J. M. A C–C Bonded Phenoxyl Radical Dimer with a Zero Bond Dissociation Free Energy. J. Am. Chem. Soc. 2013, 135 (35), 12956–12959. 10.1021/ja406500h. 23952108 PMC4084963

[ref48] Spedalotto G.; Lovisari M.; McDonald A. R. Reactivity Properties of Mixed- and High-Valent Bis(μ-Hydroxide)-Dinickel Complexes. ACS Omega 2021, 6 (42), 28162–28170. 10.1021/acsomega.1c04225. 34723014 PMC8554787

[ref49] Lovisari M.; McDonald A. R. Hydrogen Atom Transfer Oxidation by a Gold–Hydroxide Complex. Inorg. Chem. 2020, 6 (59), 3659–3665. 10.1021/acs.inorgchem.9b03225. 32125849

[ref50] Wijeratne G. B.; Corzine B.; Day V. W.; Jackson T. A. Saturation Kinetics in Phenolic O–H Bond Oxidation by a Mononuclear Mn(III)–OH Complex Derived from Dioxygen. Inorg. Chem. 2014, 53 (14), 7622–7634. 10.1021/ic500943k. 25010596

[ref51] Warren J. J.; Tronic T. A.; Mayer J. M. Thermochemistry of Proton-Coupled Electron Transfer Reagents and its Implications. Chem. Rev. 2010, 110 (12), 6961–7001. 10.1021/cr100085k. 20925411 PMC3006073

[ref52] Darcy J. W.; Koronkiewicz B.; Parada G. A.; Mayer J. M. A Continuum of Proton-Coupled Electron Transfer Reactivity. Acc. Chem. Res. 2018, 51 (10), 2391–2399. 10.1021/acs.accounts.8b00319. 30234963 PMC6197915

[ref53] Agarwal R. G.; Coste S. C.; Groff B. D.; Heuer A. M.; Noh H.; Parada G. A.; Wise C. F.; Nichols E. M.; Warren J. J.; Mayer J. M. Free Energies of Proton-Coupled Electron Transfer Reagents and Their Applications. Chem. Rev. 2022, 122 (1), 1–49. 10.1021/acs.chemrev.1c00521. 34928136 PMC9175307

[ref54] Blakely M. N.; Dedushko M. A.; Yan Poon P. C.; Villar-Acevedo G.; Kovacs J. A. Formation of a Reactive, Alkyl Thiolate-Ligated Fe^III^-Superoxo Intermediate Derived from Dioxygen. J. Am. Chem. Soc. 2019, 141 (5), 1867–1870. 10.1021/jacs.8b12670. 30661357 PMC6942688

[ref55] Ram M.; Hupp J. T. Linear free energy relations for multielectron transfer kinetics: a brief look at the Broensted/Tafel analogy. J. Phys. Chem. A 1990, 94 (6), 2378–2380. 10.1021/j100369a035.

[ref56] Weatherly S. C.; Yang I. V.; Thorp H. H. Proton-coupled electron transfer in duplex DNA: driving force dependence and isotope effects on electrocatalytic oxidation of guanine. J. Am. Chem. Soc. 2001, 123 (6), 1236–1237. 10.1021/ja003788u. 11456681

[ref57] Osako T.; Ohkubo K.; Taki M.; Tachi Y.; Fukuzumi S.; Itoh S. Oxidation mechanism of phenols by dicopper– dioxygen (Cu_2_/O_2_) complexes. J. Am. Chem. Soc. 2003, 125 (36), 11027–11033. 10.1021/ja029380+. 12952484

[ref58] Kundu S.; Miceli E.; Farquhar E. R.; Ray K. Mechanism of phenol oxidation by heterodinuclear NiCu-bis-(μ-oxo) complexes involving nucleophilic oxo groups. Dalton Trans. 2014, 43 (11), 4264–4267. 10.1039/C3DT52644E. 24362244 PMC4201338

[ref59] Wang K.; Mayer J. M. Oxidation of Hydrocarbons by [(phen)_2_Mn(μ-O)_2_Mn(phen)_2_]^3+^ via Hydrogen Atom Abstraction. J. Am. Chem. Soc. Society 1997, 119 (6), 1470–1471. 10.1021/ja963180e.

[ref60] Baldwin M. J.; Pecoraro V. L. Energetics of Proton-Coupled Electron Transfer in High-Valent Mn_2_(μ-O)_2_ Systems: Models for Water Oxidation by the Oxygen-Evolving Complex of Photosystem II. J. Am. Chem. Soc. 1996, 118, 11325–11326. 10.1021/ja9626906.

