# Supplementary material for: Class Ib Ribonucleotide Reductases: Activation of a Peroxido-MnIIMnIII to Generate a Reactive Oxo-MnIIIMnIV Oxidant
Source: Inorg Chem. 2024 Jan 17;63(4):2194–203. doi: 10.1021/acs.inorgchem.3c04163 (PMC10828993; doi:10.1021/acs.inorgchem.3c04163)
Supplement: Supplementary file 1 — ic3c04163_si_001.pdf [file ic3c04163_si_001.pdf]

Supporting Information File for:

Class Ib Ribonucleotide Reductases: Activation of a Peroxido-$Mn^{II}Mn^{III}$ to Generate a Reactive Oxo-$Mn^{III}Mn^{IV}$ Oxidant

Lorna Doyle,[a] Adriana Magherusan,[a] Shuangning Xu,[b] Kayleigh Murphy,[a] Erik R. Farquhar,[c] Florian Molton,[d] Carole Duboc,[d] Lawrence Que Jr.,[b] Aidan R. McDonald[a]*

a. School of Chemistry, Trinity College Dublin, The University of Dublin, College Green, Dublin 2, Ireland.
b. Department of Chemistry and Centre for Metals in Biocatalysis, University of Minnesota, 207 Pleasant Street SE, Minneapolis, Minnesota 55455, United States.
c. Case Western Reserve University Center for Synchrotron Biosciences, National Synchrotron Light Source II, Brookhaven National Laboratory Upton, New York 11973, United States
d. Univ. Grenoble Alpes, CNRS UMR 5250, DCM, F-38000, Grenoble, France

Email: aidan.mcdonald@tcd.ie

Materials

All reactions with air-sensitive materials were performed in a glove box under an N_2 atmosphere or under inert conditions using inert Schlenk line techniques. All reagents and solvents were purchased from commercial sources. Anhydrous *N,N*-dimethylformamide (DMF) was purchased and used without further purification. Anhydrous tetrahydrofuran (THF), acetonitrile (CH_3CN), and diethyl ether (Et_2O) were dispensed through an MBraun MB-SPS-5 Solvent Purification System and deoxygenated by purging with argon. *N,N*-bis(2-picoyl)amine and 2,6-bis(chloromethyl)-4-methylphenol,[1] 2,6-bis[(bis(2-pyridylmethyl)amino)methyl]-4-methylphenol (HBPMP),[2] $[Mn_2(O_2CCH_3)_2(BPMP)](ClO_4)$ (**1**),[3] and 4-methoxy-2,6-di-*tert*-butylphenoxyl radical,[4] were prepared as previously reported.

Cautions:

Perchlorate salts of metal complexes and KO_2 are potentially explosive and must be handled with care and in small quantities. We limited our preparations of **1** to a maximum of 0.2 g per reaction.

WARNING - PREPARATION AND STORAGE OF $K^{18}O_2$:

We had a minor explosion, ***inside our glovebox***, when handling (using a metal spatula) a small quantity (20-40 mg) of our in-house prepared $K^{18}O_2$ in a glass Schlenk flask. There was no spark or initial flame, the ignition was rapid and forceful. The explosion caused the Schlenk flask (volume = 20 mL) to shatter, but no injury to the researcher. We believe injury was avoided because the explosion occurred inside the glovebox, while the researcher was protected from the shattering glass by the glovebox structure and heavy-duty gloves. A more serious outcome was likely had the explosion not been inside the glovebox. The water content in the glovebox was below detectable levels at the time. We tentatively postulate that the sample contained some K_2O_2. K_2O_2 is considerably more shock-sensitive than KO_2. As a result

of this we have decided to not use/store/prepare $K^{18}O_2$ locally again. The method of $K^{18}O_2$ preparation was from a previously reported procedure, reacting TEMPO-K with $^{18}O_2$.[5]

Physical Methods

Nuclear magnetic resonance (NMR) analyses were performed on a Bruker Avance III 400 MHz instrument. Electrospray ionisation (ESI) mass spectrometry was obtained using a micromass time of flight spectrometer (TOF), interfaced to a Waters 2690 HPLC, or by direct injection in the mass spectrometry instrument. Electronic absorption spectra were recorded in quartz cuvettes on a Hewlett Packard (Agilent) 8453 diode array spectrophotometer (190 – 1100 nm range) coupled to a liquid nitrogen cooled cryostat from Unisoku Scientific Instruments (Osaka, Japan).

Electron paramagnetic resonance (EPR) measurements were conducted on a Bruker Elexsys E-500 spectrometer with an Oxford ESR 910 liquid helium cryostat and an Oxford temperature controller. The quantification of EPR signals measured at 30 K was against a $[Cu^{II}(NO_3)_2]$ spin standard in CH_3OH. The quantification of EPR signals measured at 77 K were relative to a TEMPO spin standard in 1:9 CH_3CN/THF. EPR spectra of 4-X-TEMPO and ABNO were recorded at 77 K, 9.2 GHz, 2.02 mW microwave power, with a 60 mT field sweep in 84 s, and 0.3 mT field modulation amplitude. Integration, simulation, and fitting were performed with Matlab and the easySpin computational package.[6]

X-ray absorption spectroscopy (XAS) methods: The Mn-K-edge X-ray absorption data were collected on beamline 7-3 of the Stanford Synchrotron Radiation Lightsource (SLAC National Accelerator Lab, Menlo Park, CA, USA). Data were collected with the storage ring operating at 3.0 GeV and 500 mA, using a LN2 cooled Si(220), $\phi = 90^{\circ}$ double-crystal monochromator,

calibrated by using the first inflection point of a Mn foil (6539.0 eV). The monochromator was detuned by ~50% for higher harmonic rejection. A Canberra 32-element solid state germanium detector was used for fluorescence detection. All measurements were performed at ambient pressure at ~17 K using an Oxford helium cryostat that was cooled by closed-cycle He gas loop. The parameters used for the scans were the following: 10 eV steps/1 second integration time in the pre-edge region, 0.3 eV steps /2 second integration time in the edge, and 0.05k steps above the edge, with integration time increasing in a k^2 -weighted fashion from 2 to 4 seconds over the selected energy range (k_{max} = 12.1k). The total detector counts were typically 3-7k, well within the linear range of the detector electronics. Each sample was monitored for photoreduction. Complexes **2**, **3**, and **4** were found to be photoreduced by the X-rays and so only one scan was obtained per spot. Evaluation of the XAS data, including averaging, background removal and normalization, was performed using Athena.[7] Edge energies were obtained by taking the first derivative of the rising edge, while pre-edge energies were identified using the second derivative.

Experimental Procedures

*General procedure for the preparation of **2**:*

A CH_3CN/THF (1:9) solution of **1** (1.5 mM) was prepared. In a quartz cuvette, 2 mL of this solution was cooled to -90 °C. KO_2 (0.0056 g, 0.04 M) and cryptand (0.06 g, 0.08 M) were dissolved in DMF (2 mL). 75 μL of this solution was added to the solution containing **1**. The reaction progress was monitored using electronic absorption spectroscopy. Lower yields were observed (less intense absorption features) with higher equivalencies of KO_2 and at higher concentrations of **1** as was previously observed.[8-10]

*General procedure for the preparation of **3**:*

2 was prepared as above. *para*-Toluene sulfonic acid (*p*-TsOH) was dissolved in 1:9 CH_3CN/THF (0.095g, 0.55 M). This solution was added to the solution containing **2** (volume depended on the stoichiometry required). The reaction progress was monitored using electronic absorption spectroscopy.

*General procedure for the preparation of **4**:*

2 was prepared as above at -90 °C. The temperature controller on the Unisoku cryostat was subsequently programmed to 20 °C resulting in the gradual warming of the solution to 20 °C. The reaction progress was monitored using electronic absorption spectroscopy.

Synthesis of ABNO-H

ABNO-H was synthesised used an adapted procedure.[11] $Na_2S_2O_4$ (1.00 g, 5.7 mmol) was added to a degassed solution of ABNO (500 mg, 3.7 mmol) dissolved in 1:1 acetone/water (15 ml) resulting in a colour change from orange to white. The acetone was removed under vacuum and the resulting aqueous solution was extracted with deoxygenated pentane (3 x 10 ml). The pentane was removed under vacuum to yield a white precipitate and triturated with ~15 ml Et_2O followed by solvent removal under vacuum (yield = 342 mg, 70%). The ^1H NMR spectrum was consistent with previously published spectra of ABNO-H.[12]

Calculation of ABNO-H BDFE in CH_3CN[13, 14]:

$$BDFE_g = BDE_g - TS°(H°)$$

$$ABNO\ BDE_g = 68.2\ kcal\, mol^{-1}$$
$$S°(H°) = 27.42\ cal\, K^{-1} mol^{-1}$$
$$BDFE_g = 63.179\ kcal\, mol^{-1}$$

$$BDFE_{solv} = BDFE_g + \Delta G°_{solv}(H°) + [\Delta G°(X°) - \Delta G°(XH)]$$

$$\Delta G°_{solv}(H°) = 5.11\ kcal\, mol^{-1}\ in\ CH_3CN$$

$$\Delta G° = -10.02\alpha_2^H\beta_2^H - 1.492$$
$$\alpha_2^H = 0.39\ (TEMPO - H)\ \beta_2^H = 0.44\ (CH_3CN)$$
$$\Delta G° = 3.21$$

$$ABNO - H\ BDFE_{MeCN} = 71.49\ kcal\ mol^{-1}$$

EPR sample preparation:

Samples for EPR analysis were prepared by transferring ~ 1 mL of the desired solution from the quartz cuvette at -90 °C, via a pre-cooled pipette into a pre-cooled EPR tube and immediately freezing it in liquid nitrogen. The EPR spectra were recorded at 2 K (9.64 GHz, 0.2 mW microwave power).

ESI-MS sample preparation:

The samples for frozen MS analysis were prepared by transferring ~ 0.1 mL of the desired solution from the quartz cuvette, pre-cooled to -90 °C, via a pre-cooled sample vial and immediately freezing in liquid nitrogen. ESI- MS was performed on the just thawed sample. Once the sample had started to melt it was taken into a pre-cooled syringe and rapidly injected directly into our micromass time of flight mass spectrometry instrument. The spray-head temperature was 180 °C and the accelerating voltage was 4,000 V.

Reactivity Studies:

3 and **4** were prepared as described above. Substrates were added as concentrated CH_3CN/THF solutions to solutions of **3** at -90 °C or **4** at +20 °C. The reactions were monitored using electronic absorption spectroscopy.

Note on the Hill Type kinetics observed in the reaction of ***4*** *and 4-CN-2,6-DTBP:*

Hill models are displayed by enzymes that display cooperativity (n). A value of $n > 1$ in a system displaying non-linear k_{obs} vs [S] (S = substrate) behaviour may be understood as more than one substrate molecule is involved in the reaction. The cooperativity for the reaction between **4** and 4-CN-2,6-DTBP was calculated to be 2.95 $\pm$ 1. The change from Michaelis-Menten behaviour to a Hill type may be attributed to an increase in acidity of the O-H bond of 4-CN-2,6-DTBP.

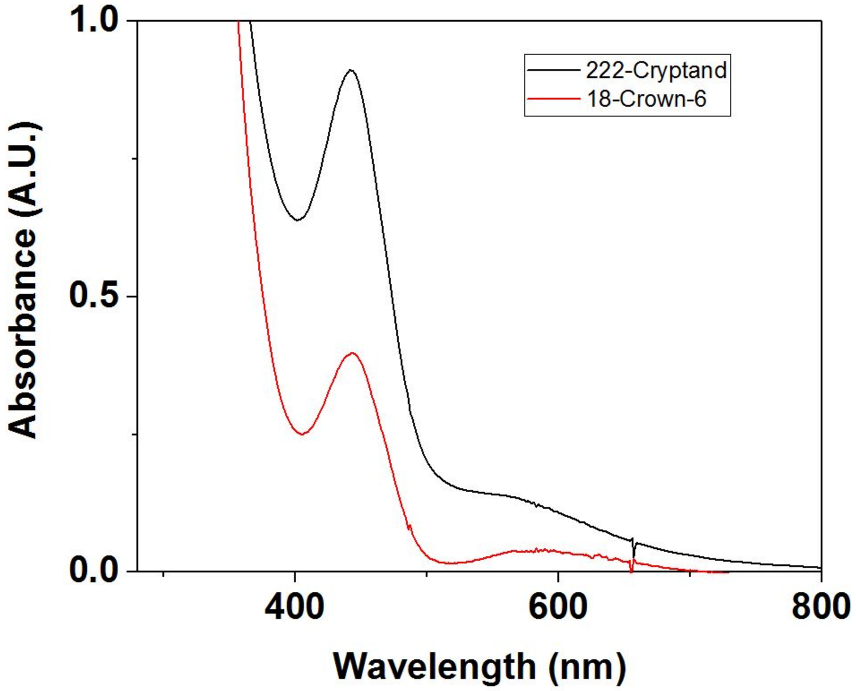

Figure S1. Electronic absorption spectra of **2** generated from the reaction of **1** (1.5 mM) with KO_2 using 2,2,2-cryptand (black trace) and 18-crown-6 (red trace) in 1:9 CH_3CN/THF at -90 °C.

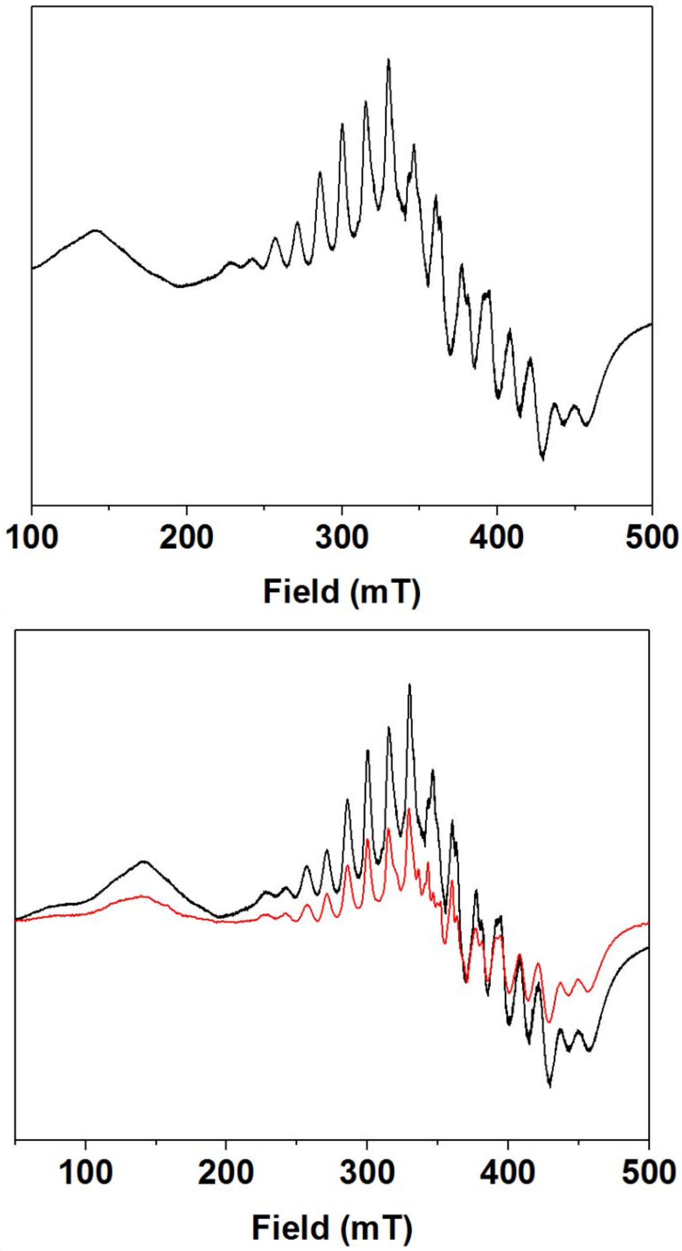

Figure S2. Top: Perpendicular mode X-band EPR spectrum of **2** at 2 K (black trace) obtained from the reaction of **1** (1.5 mM) with KO_2 solubilised with 2,2,2-cryptand. Bottom: comparison of X-band EPR spectrum of **2** prepared using 2,2,2-cryptand (black trace) or 18-crown-6 (red trace) in 1:9 CH_3CN:THF (9.64 GHz, 0.2 mW microwave power).

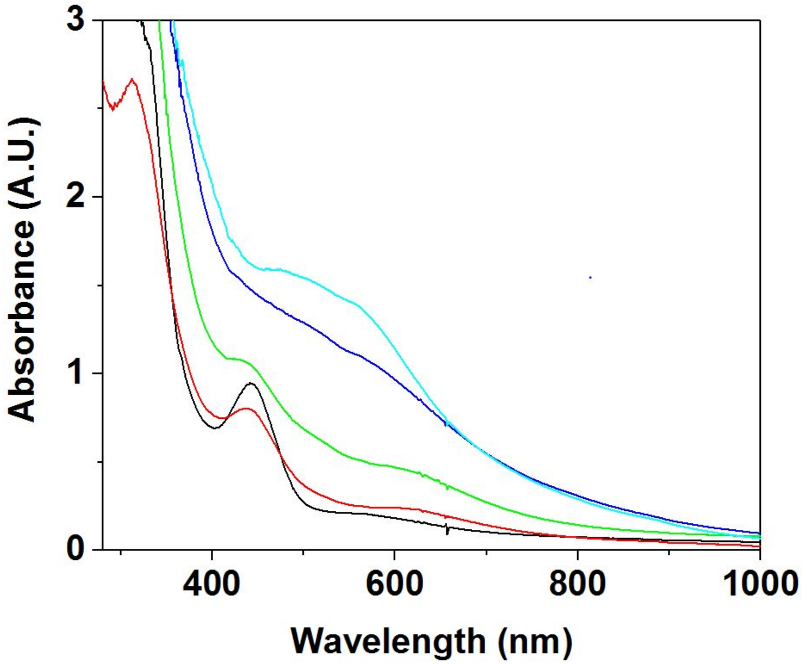

Figure S3. Comparison of electronic absorption spectra of **2** (black) and when *p*-TsOH (0.5 equiv., red trace; 1 equiv., green trace; 1.5 equiv., blue trace; and 2 equiv. cyan trace) was added.

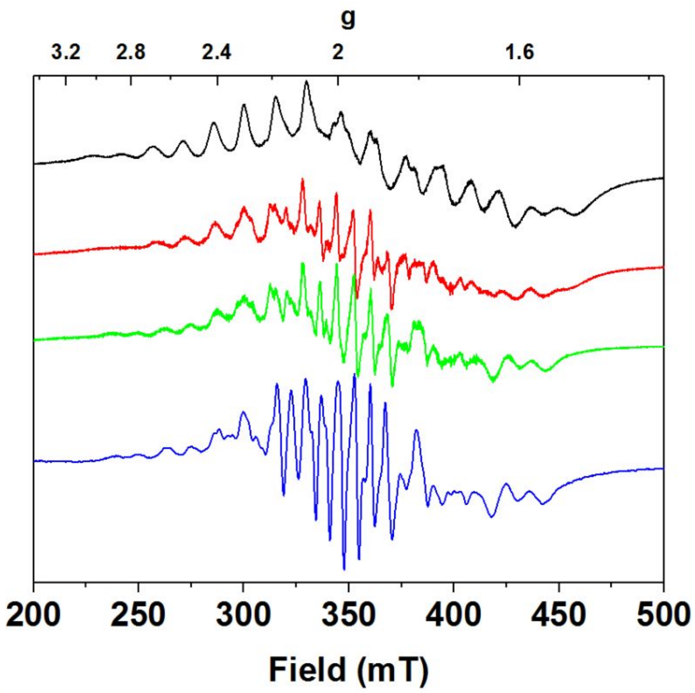

Figure S4. Perpendicular mode X-Band EPR spectra of the post-reaction mixture of **2** (black) and when *p*-TsOH (0.5 equiv., red trace; 1.0 equiv., green trace; 1.5 equiv., blue trace) were added. Measured at 2 K (9.65 GHz, 0.2 mW microwave power).

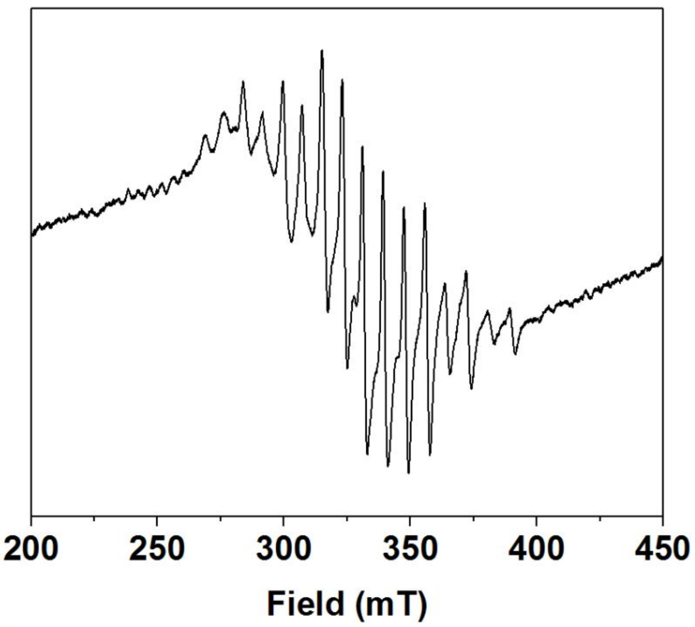

Figure S5. Perpendicular X-Band EPR spectrum of **3** (generated from **2** + 2 equiv. of *p*-TsOH in 1:9 CH_3CN/THF at -90 °C) at 77 K (9.65 GHz, 0.2 mW microwave power).

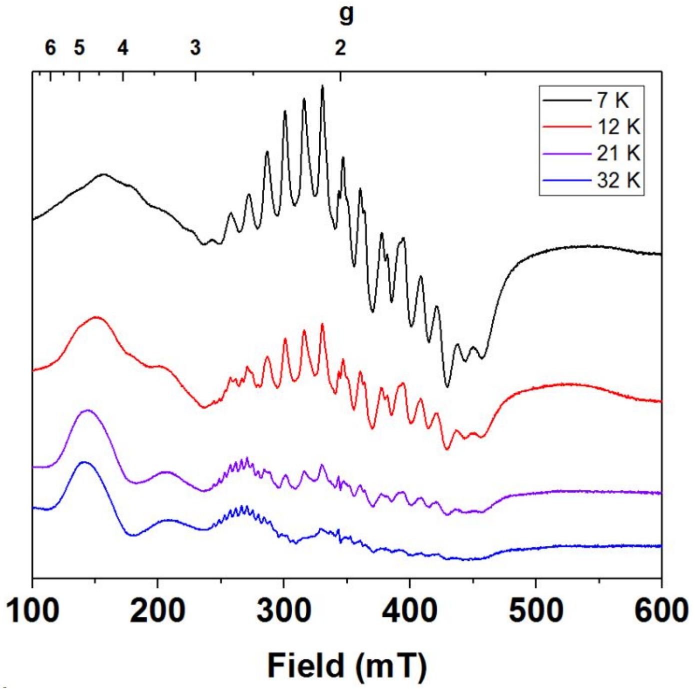

Figure S6. Perpendicular X-Band EPR spectrum of **2** measured at 7 K (black), 12 K (red), 21 K (purple), 32 K (blue) (9.65 GHz, 0.2 mW microwave power).

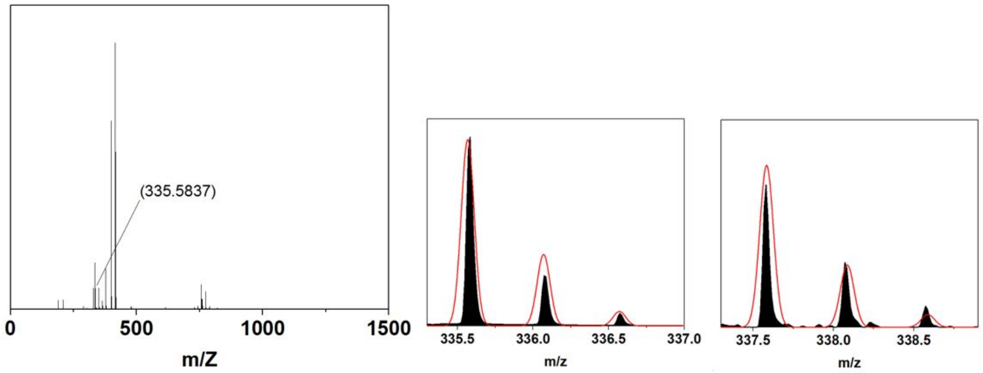

Figure S7. Left and centre: ESI-MS spectra of **3** prepared using $K^{16}O_2$ (black trace) with simulated spectrum (red trace). Right: ESI-MS spectra of **3** prepared using $K^{18}O_2$ (black trace) with simulated isotopic spectrum (red trace).

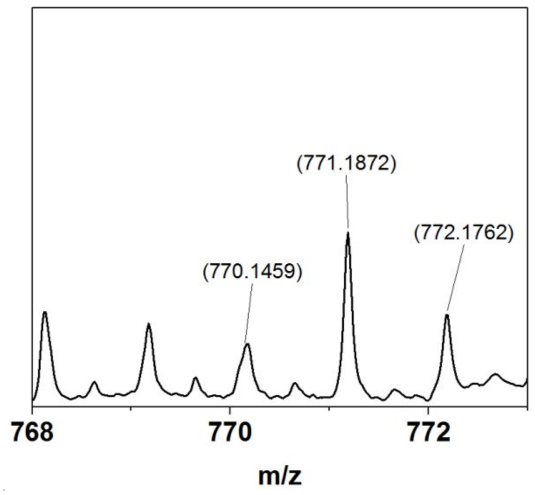

Figure S8. ESI-MS of sample containing **3** expanded in the regions where **2** is found.

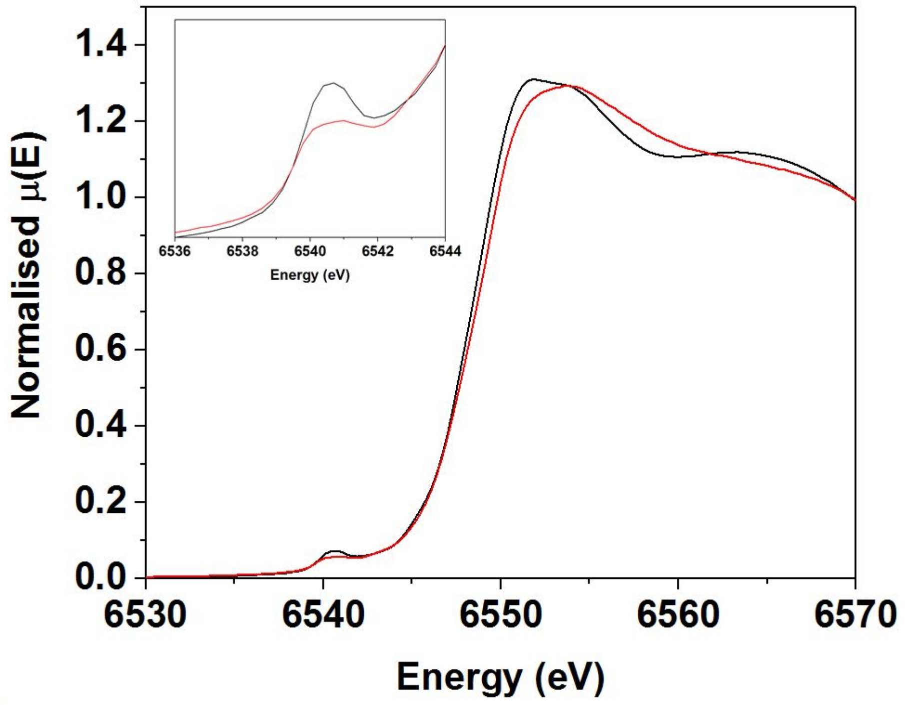

Figure S9. Normalised XANES spectra of **2** (black trace) and **3** (red trace). Inset: Expansion of the pre-edge region.

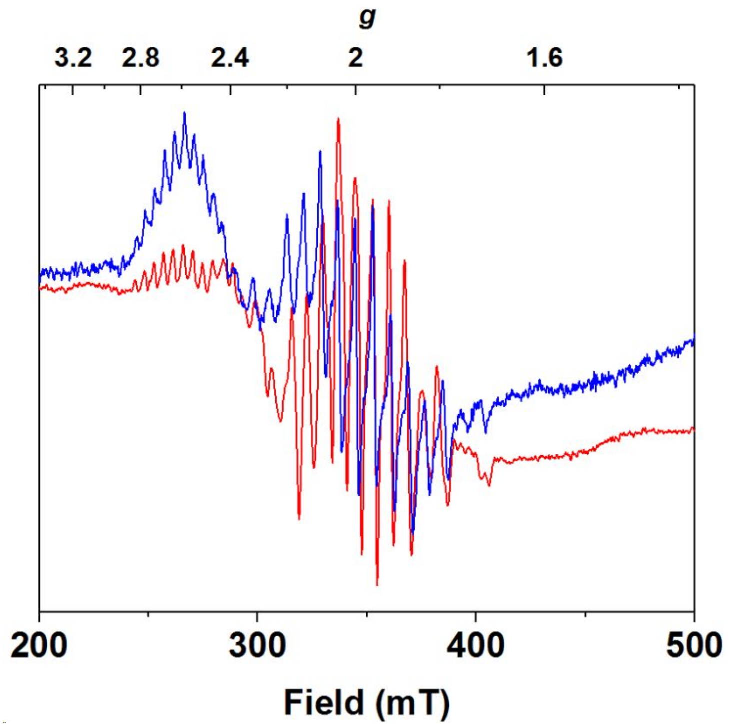

Figure S10. Comparison of the EPR spectra of **3** (red trace) and **4** (blue trace).

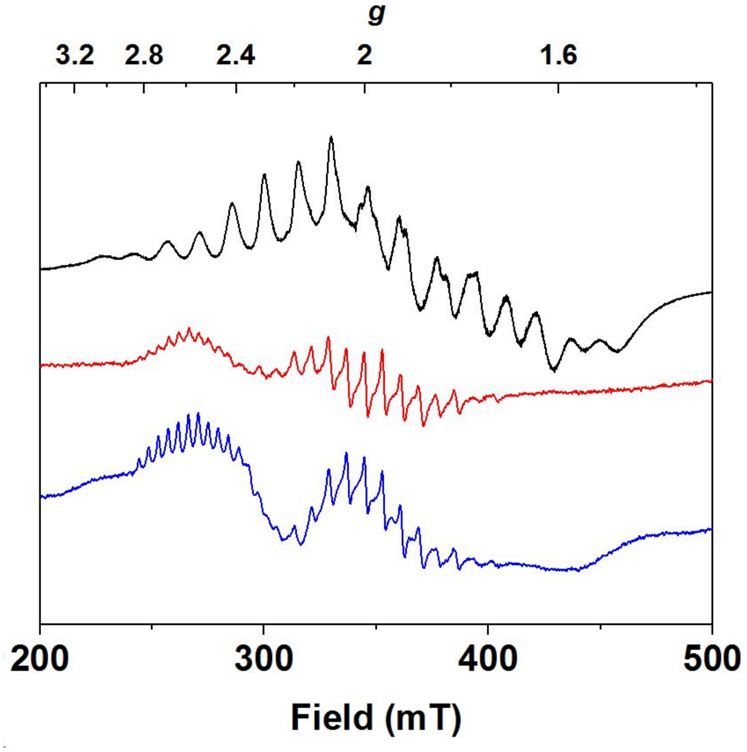

Figure S11. EPR spectra of **2** recorded at 2 K (black trace), **4** recorded at 7 K (red trace) and 30 K (blue trace) (9.65 GHz, 0.2 mW microwave power).

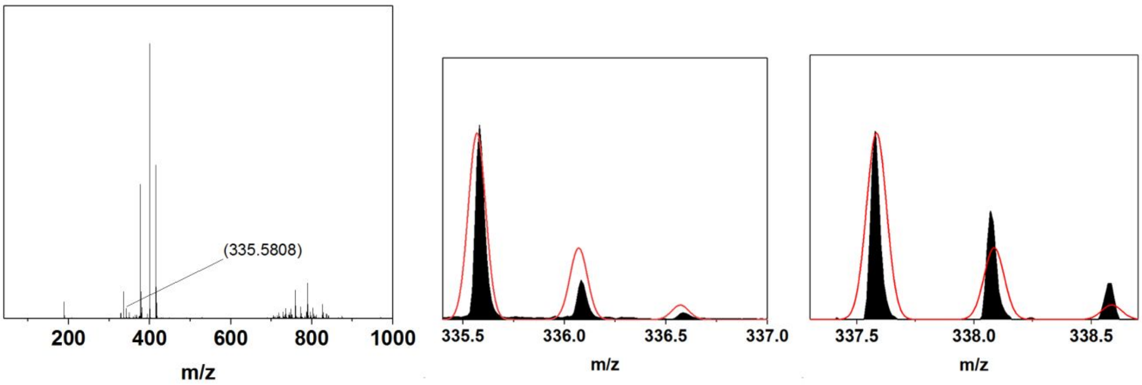

Figure S12. Left and centre: ESI-MS of **4** prepared using $K^{16}O_2$; and Right: ESI-MS of **4** prepared using $K^{18}O_2$ (right). Red traces = simulated mass spectra.

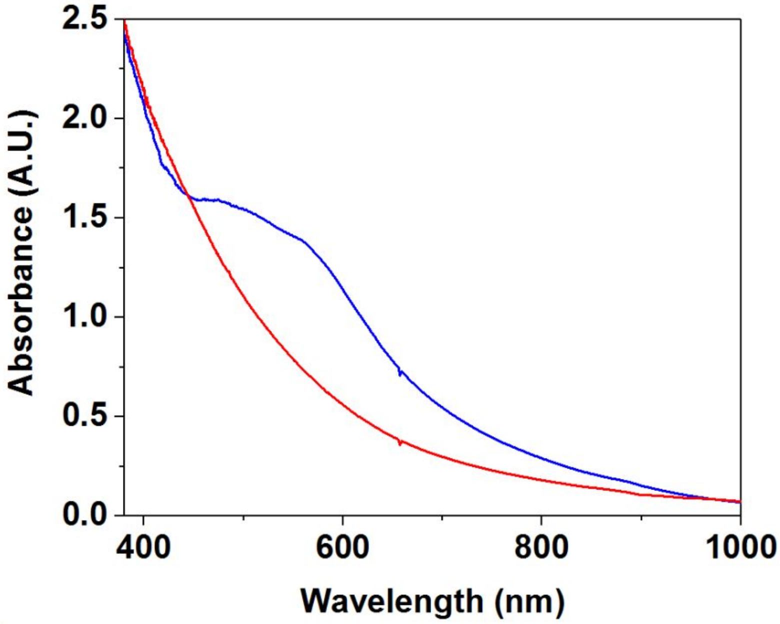

Figure S13. Comparison of the electronic absorption spectra of **3** (blue trace, generated from **2** + 2 equiv. *p*-TsOH, 1:9 CH_3CN/THF, at -90 °C) and **4** (red trace, generated from **2** heated from -90 °C → +20 °C, 1:9 CH_3CN/THF, measured at +20 °C).

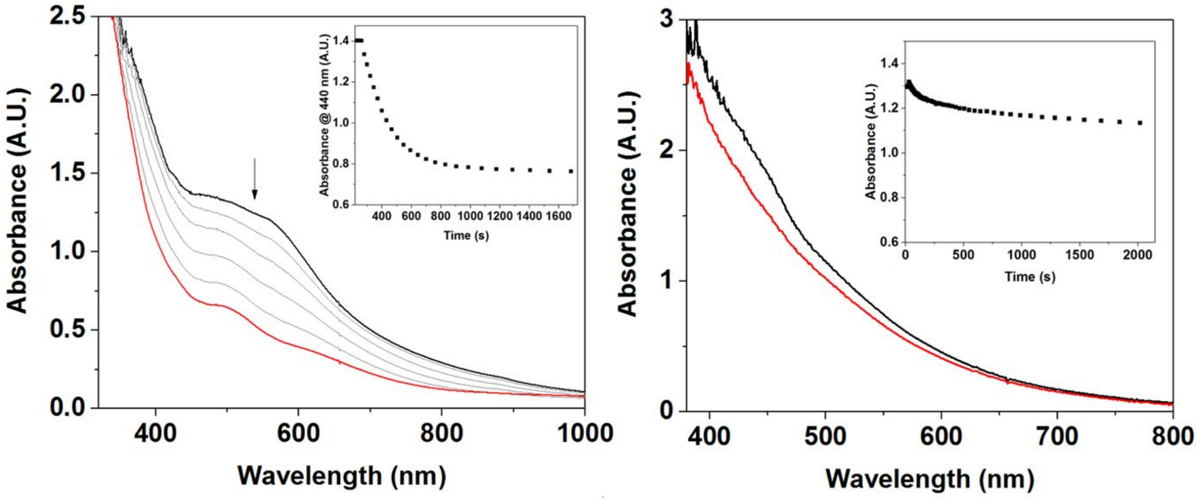

Figure S14. Left: electronic absorption changes during the reaction of **3** with NEt_3 (100 equiv.) Right: electronic absorption features of the reaction of **4** with NEt_3 (100 equiv.) Inset: plot of the absorbance at 440 nm versus time during reaction.

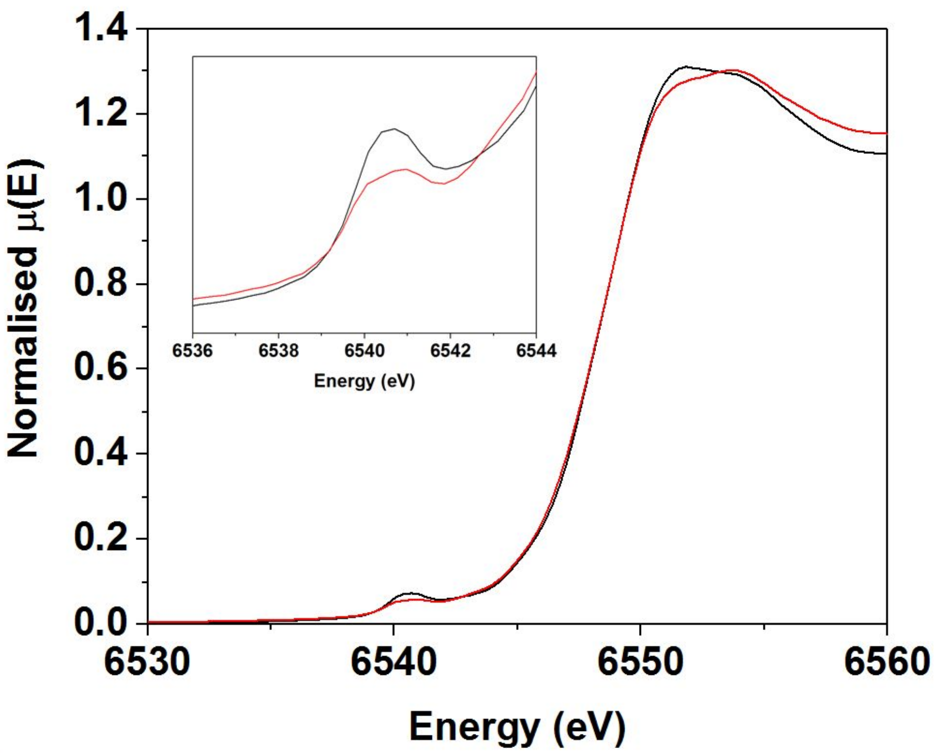

Figure S15. Normalised XANES spectra of **2** (black trace) and **4** (red trace). Inset: Expansion of the pre-edge region. As with the XANES analysis of **3**, upon conversion of **2** to **4** the edge energy shifted minimally (< 0.5 eV). As with **3**, irradiation of **4** resulted in a colour change

indicating photo-decay of the species of interest. We attributed the lack of a shift to the following: photo-reduction of **4** by incident X-rays during the XAS measurement *and* a less than optimal yield of **4**, only ~60 ± 20 % by EPR integration. We concluded that XAS was not going to provide insight into the electronic and geometric structures of **4**.

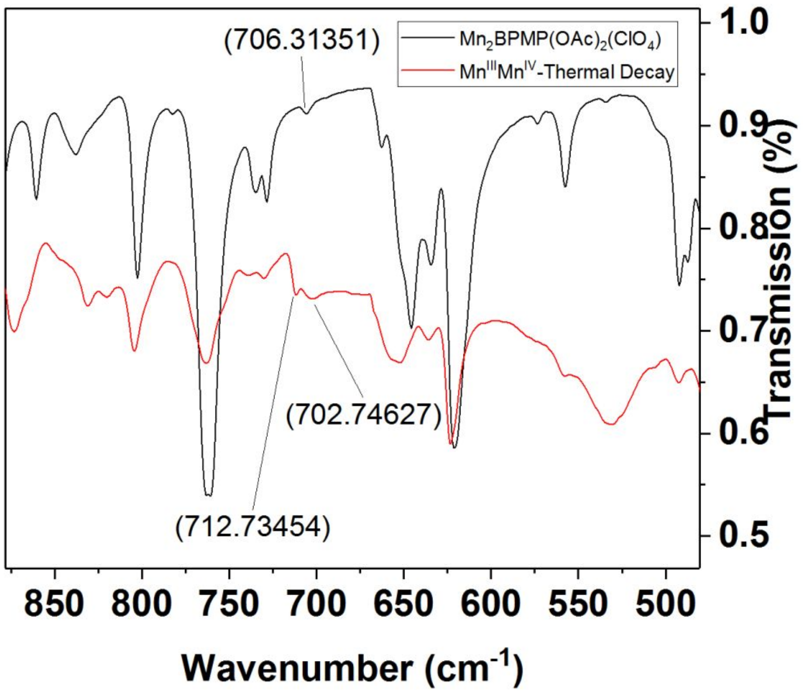

Figure S16. FT-IR spectra of **1** and **4**.

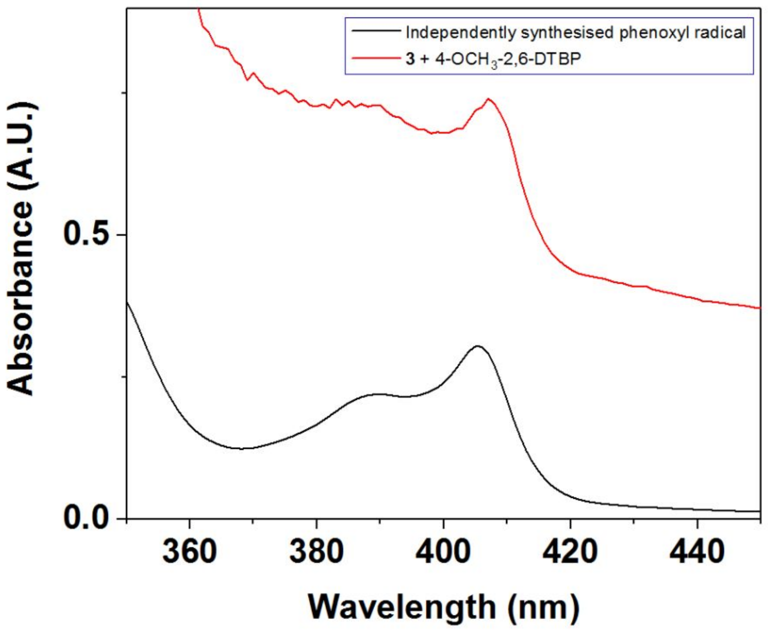

Figure S17. Post-reaction electronic absorption spectrum from the reaction of **3** with 4-CH_3O-2,6-DTBP (red trace) and independently synthesised 4-CH_3O-2,6-di-*tert*-butylphenoxyl radical (black trace).

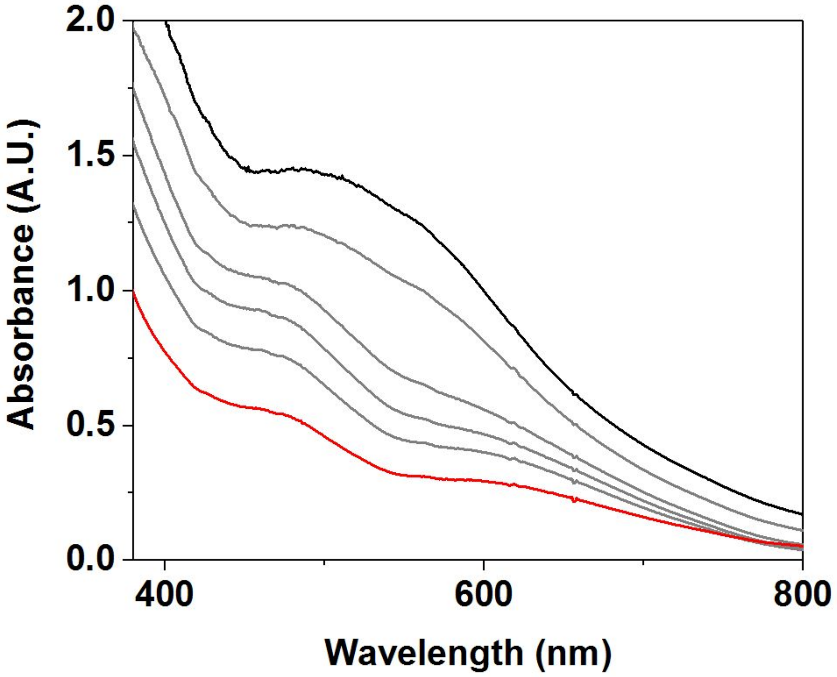

Figure S18. Electronic absorption spectra changes during the reaction of **3** (black trace) with ABNO-H (10 equiv., red trace is spectrum at end of the reaction).

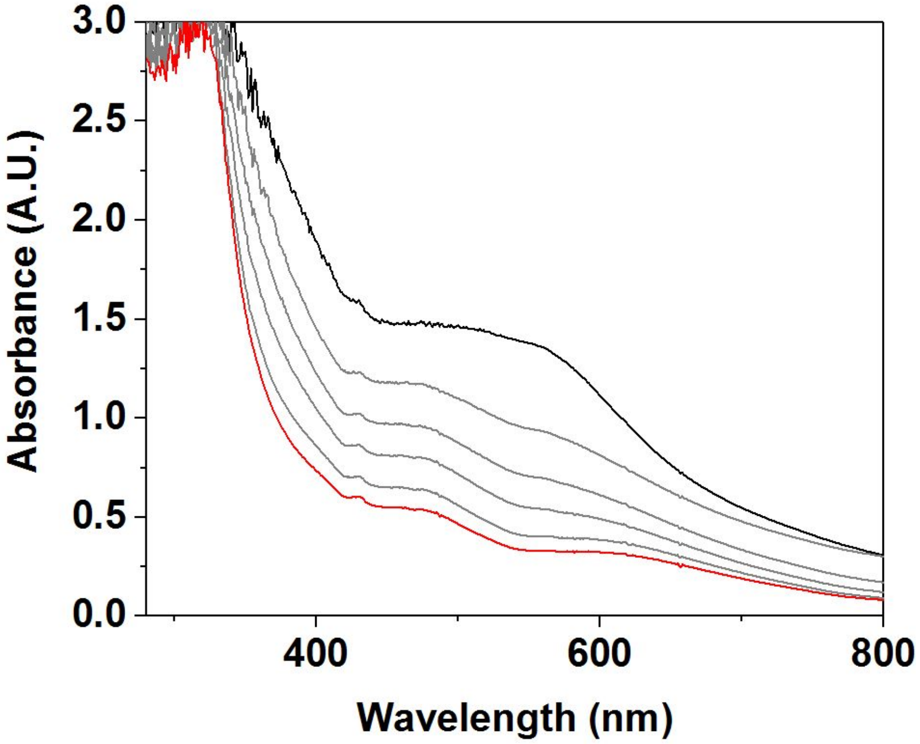

Figure S19. Electronic absorption spectra changes during the reaction of **3** (black trace) with TEMPO-H (40 equiv., red trace is spectrum at end of the reaction).

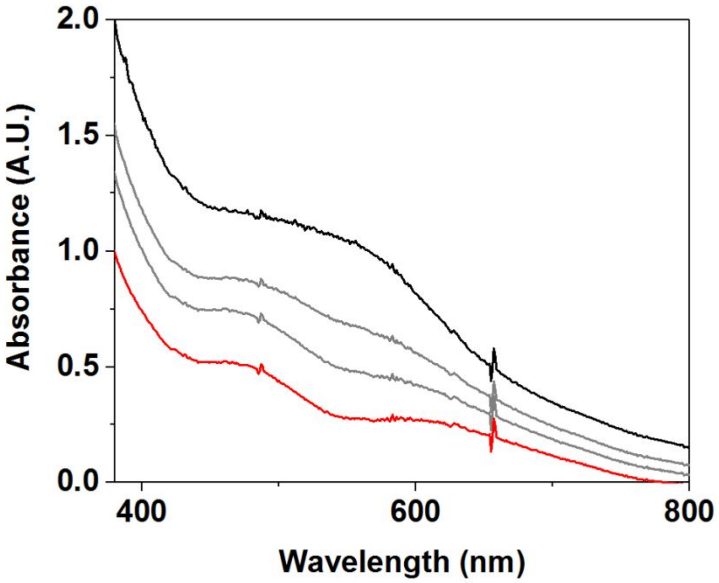

Figure S20. Electronic absorption spectra changes during the reaction of **3** (black trace) with 4-CH_3O-TEMPO-H (30 equiv., red trace is spectrum at end of the reaction).

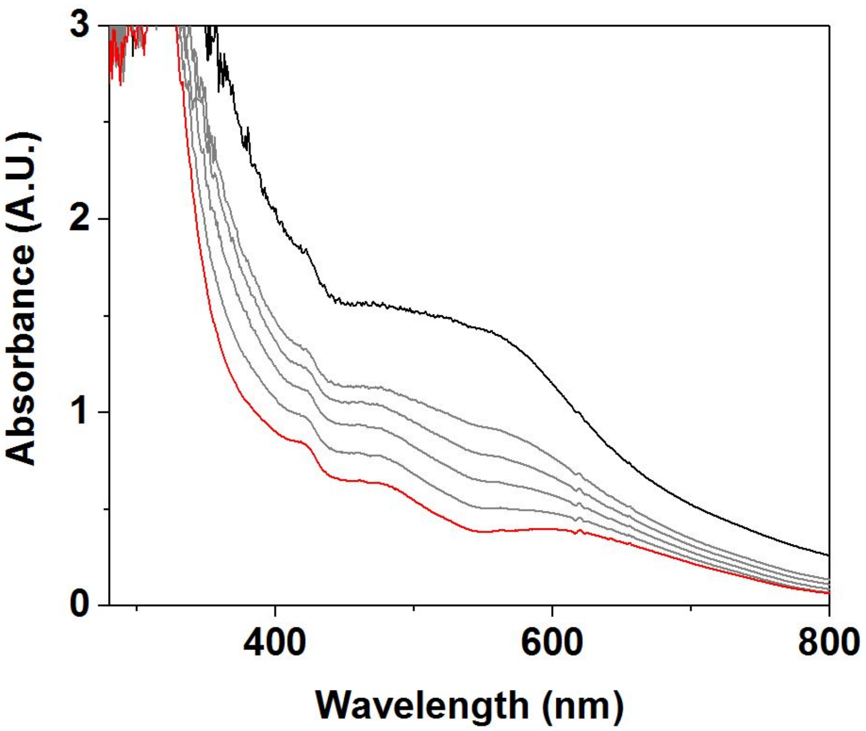

Figure S21. Electronic absorption spectra changes during the reaction of **3** (black trace) with 4-Oxo-TEMPO-H (30 equiv., red trace is spectrum at end of the reaction).

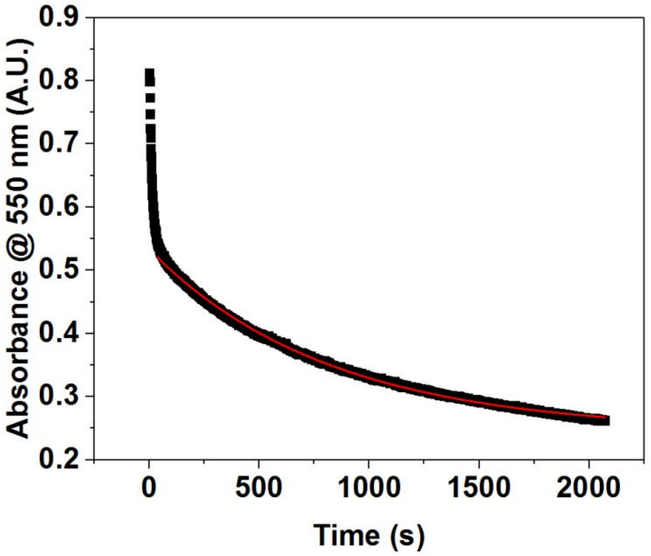

Figure S22. Plot of the absorbance at λ = 550 nm during the reaction of **3** and ABNO-H (black trace). First-order fit of the decay (red trace)

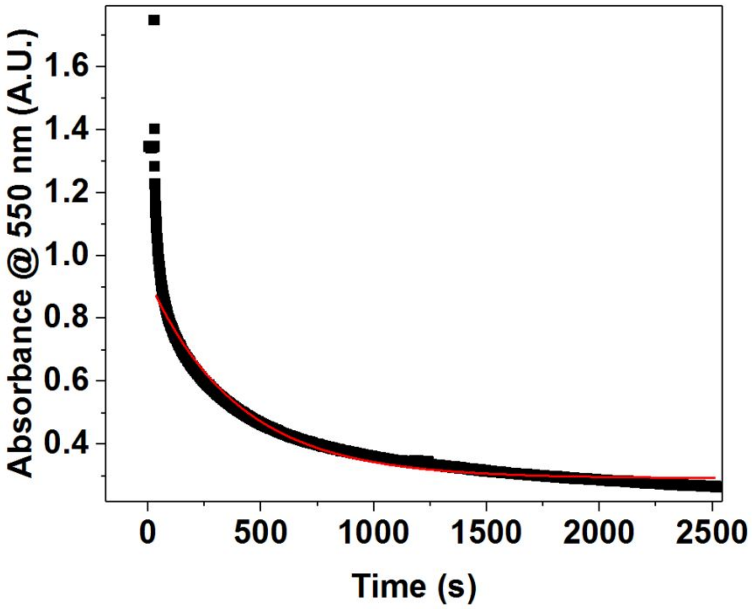

Figure S23. Plot of the absorbance at λ = 550 nm during the reaction of **3** and TEMPO-H (black trace). First-order fit of the decay (red trace).

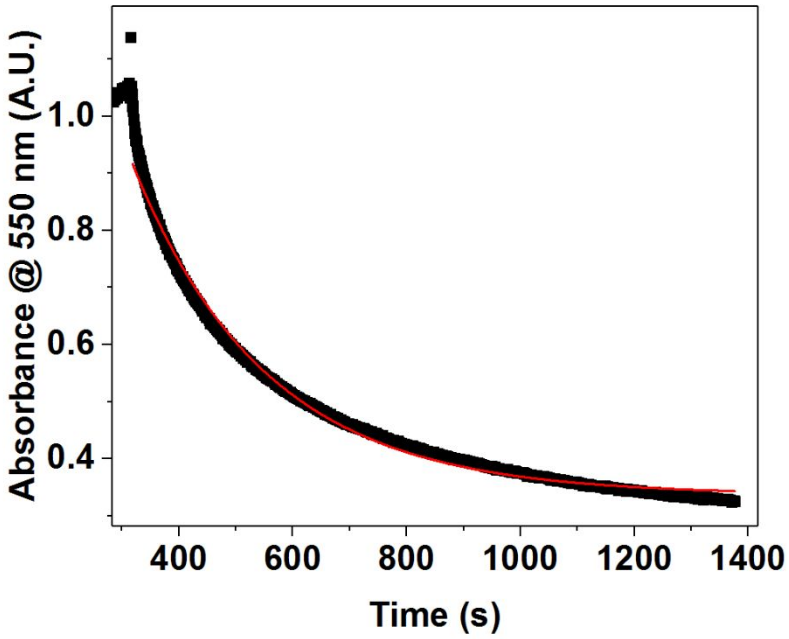

Figure S24. Plot of the absorbance at λ = 550 nm during the reaction of **3** and 4-CH_3O-TEMPO-H (black trace). First-order fit of the decay (red trace)

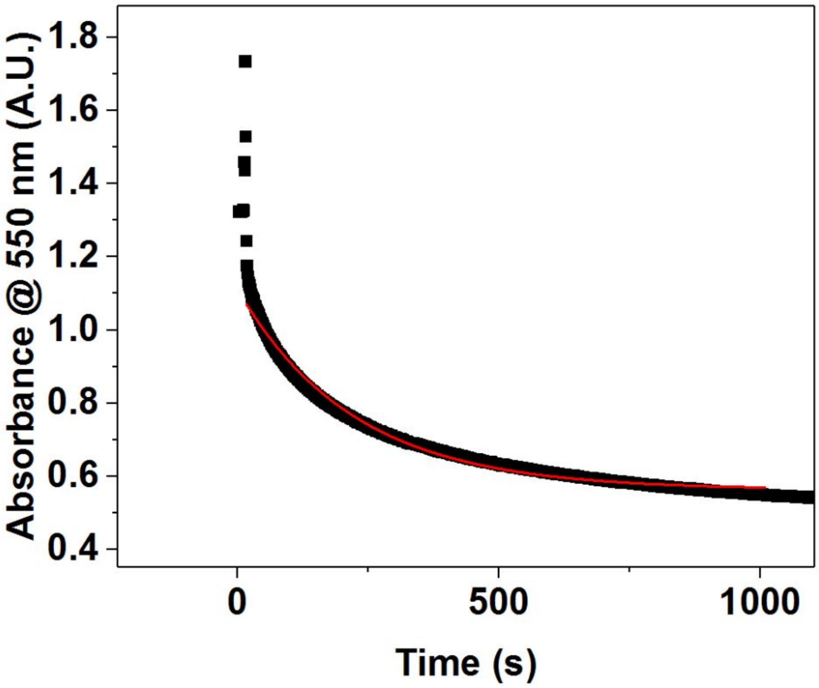

Figure S25. Plot of the absorbance at λ = 550 nm during the reaction of **3** and 4-oxo-TEMPO-H (black trace). First-order fit of the decay (red trace).

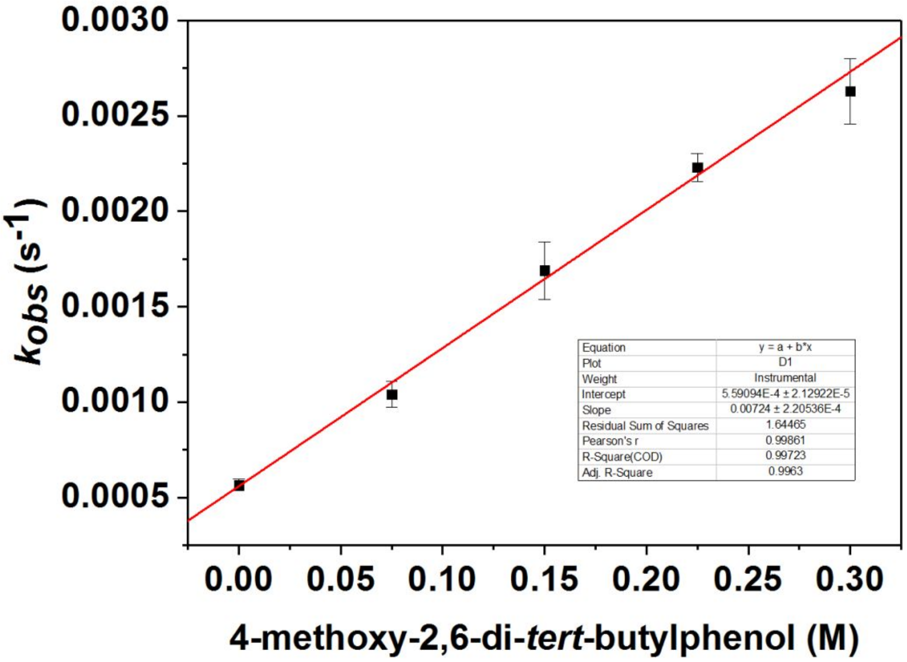

Figure S26. Plot of k_{obs} versus [S] determined for the reaction of **3** and 4-CH_3O-2,6-DTBP.

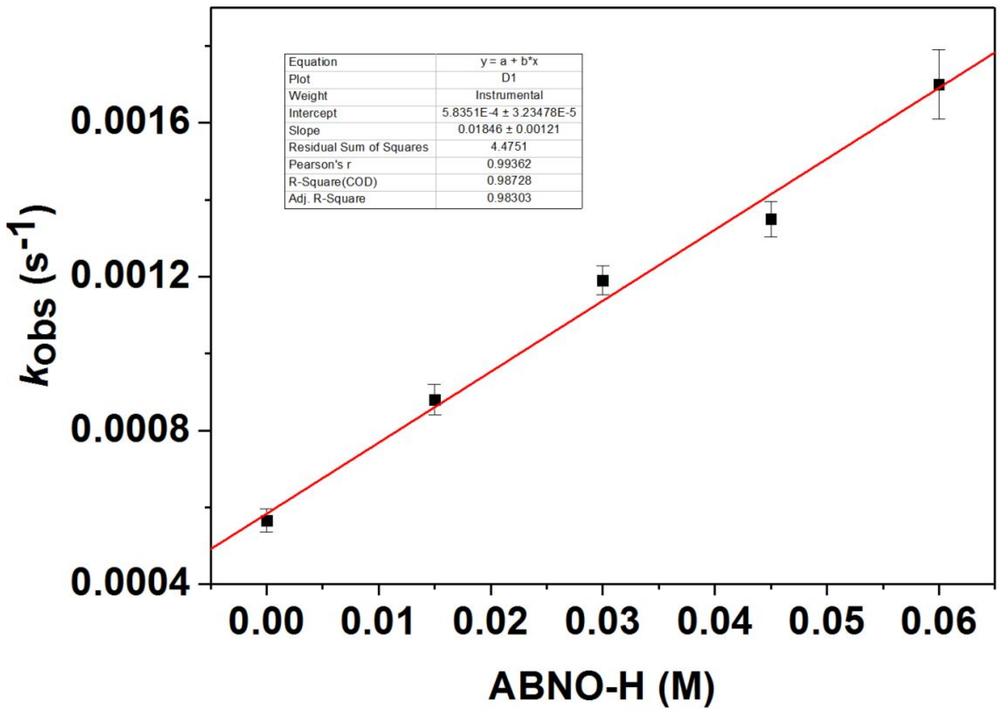

Figure S27. Plot of k_{obs} versus [S] determined for the reaction of **3** and ABNO-H.

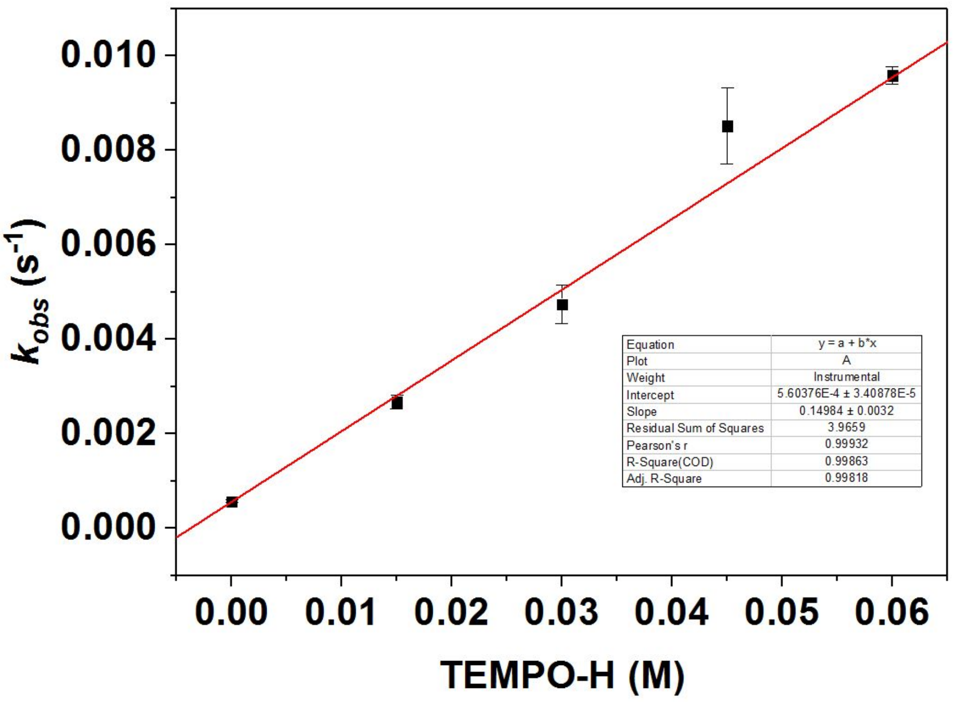

Figure S28. Plot of k_{obs} versus [S] determined for the reaction of **3** and TEMPO-H.

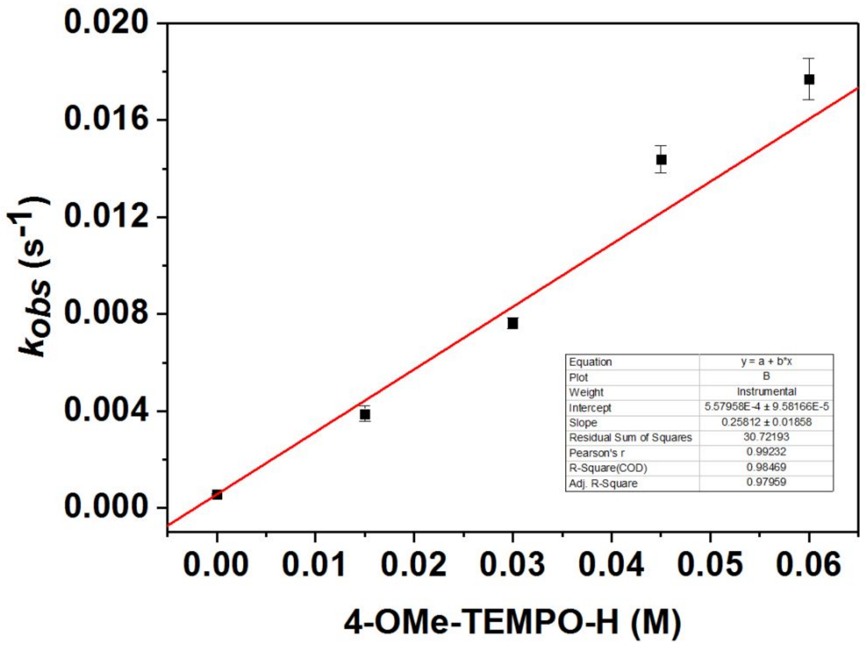

Figure S29. Plot of k_{obs} versus [S] determined for the reaction of **3** and 4-CH_3O-TEMPO-H.

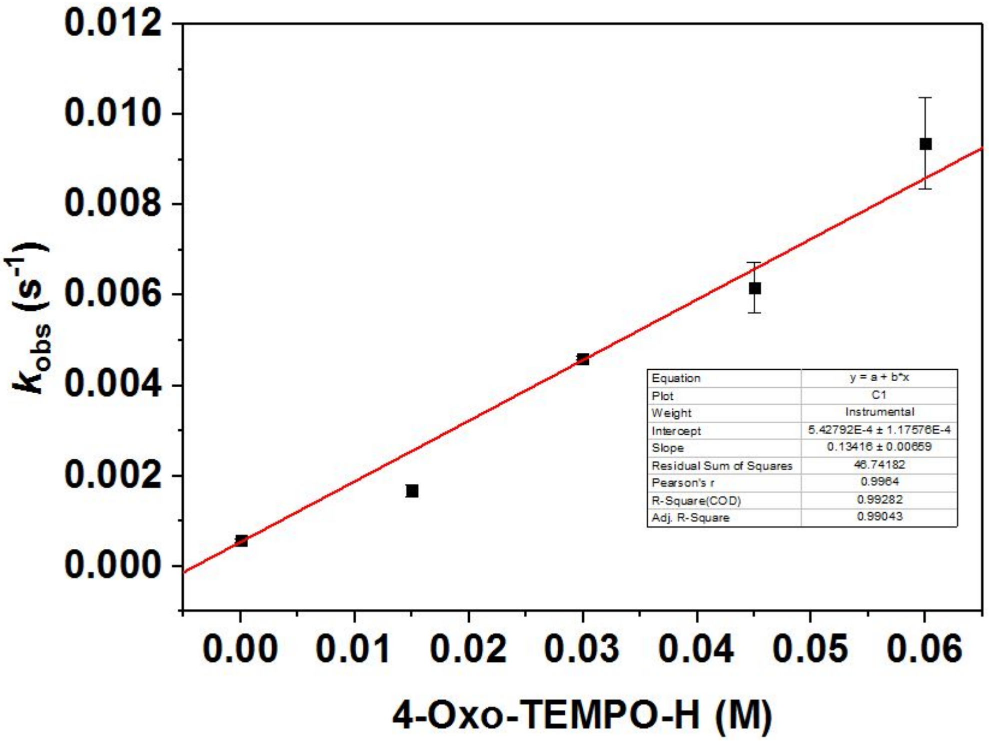

Figure S30. Plot of k_{obs} versus [S] determined for the reaction of **3** and 4-oxo-TEMPO-H.

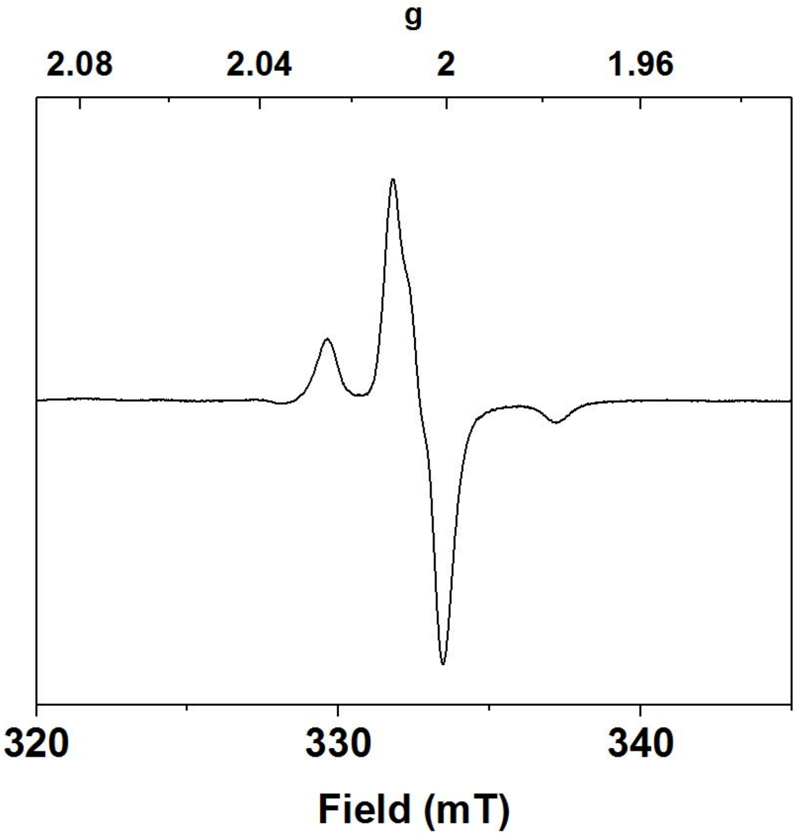

Figure S31. EPR spectrum from the reaction of **3** with ABNO-H.

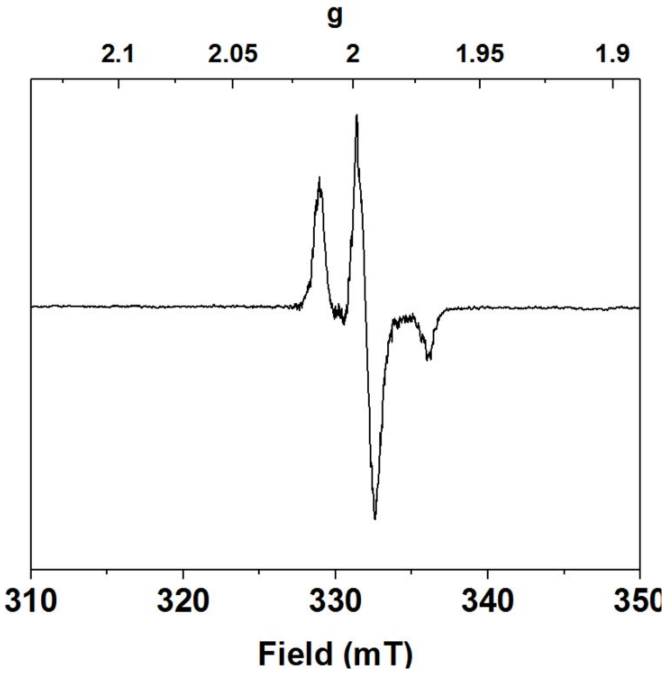

Figure S32. EPR spectrum from the reaction of **3** with TEMPO-H

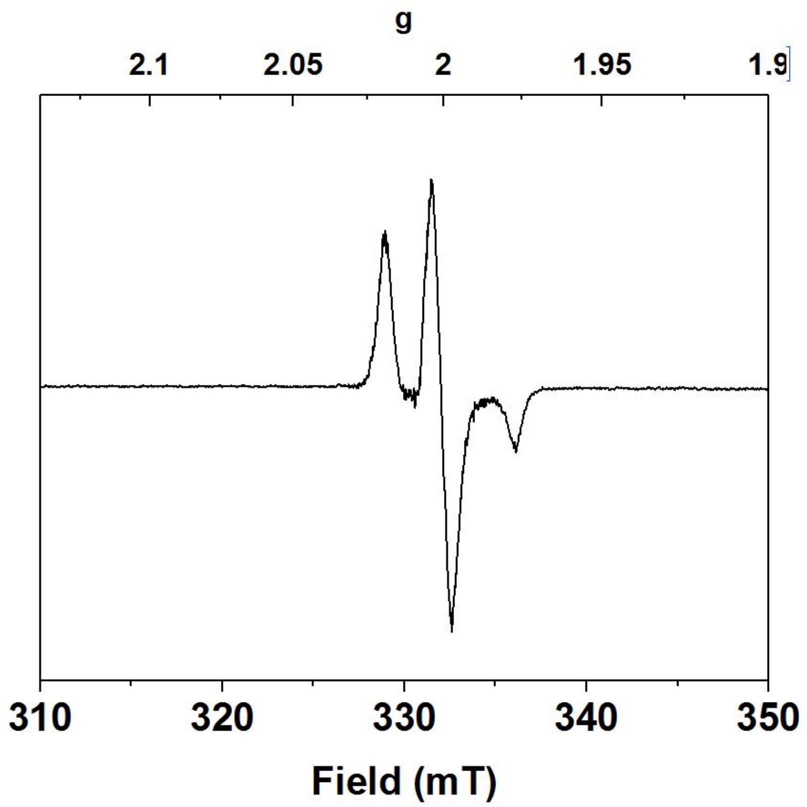

Figure S33. EPR spectrum from the reaction of **3** with 4-CH_3O-TEMPO-H.

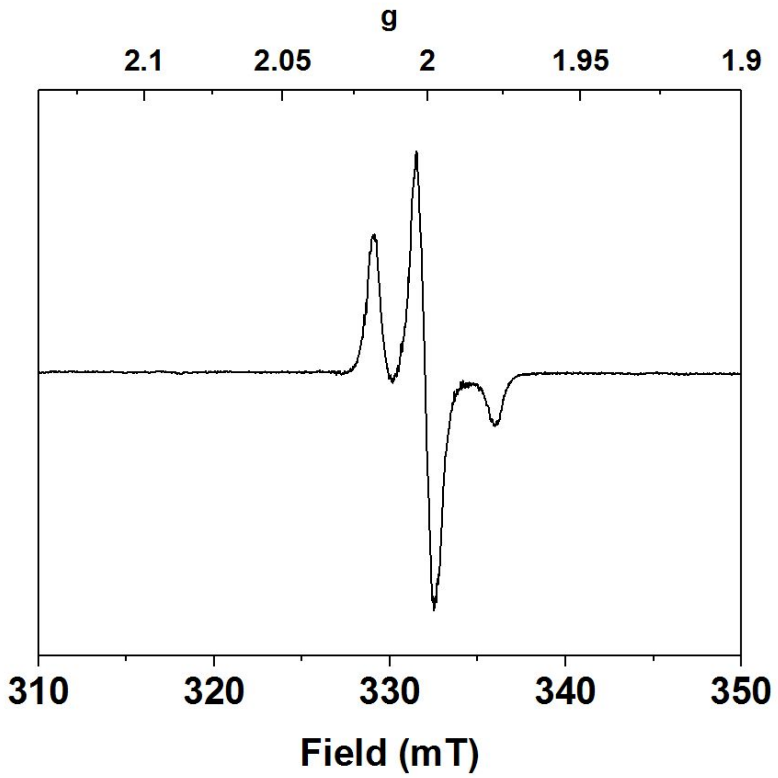

Figure S34. EPR spectrum from the reaction of **3** with 4-oxo-TEMPO-H

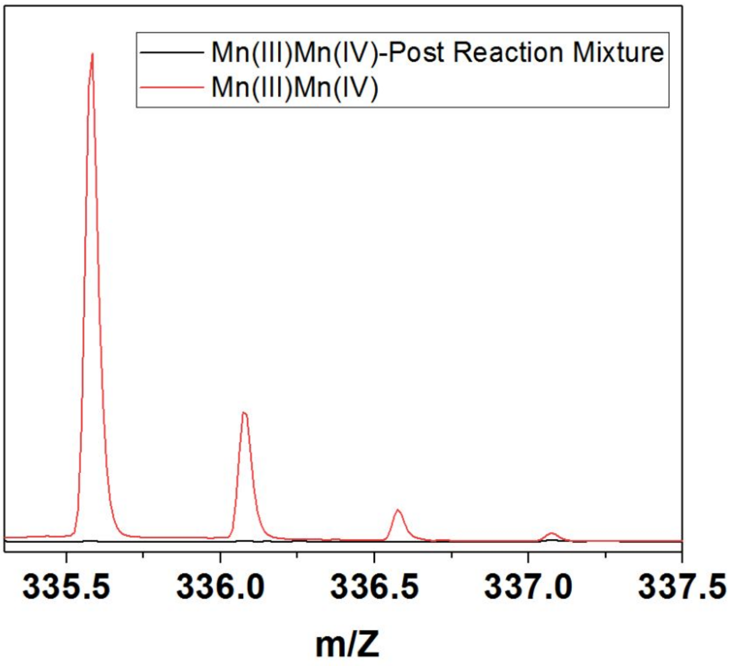

Figure S35. ESI-MS spectra of the post-reaction mixture from the reaction of **3** with TEMPO-H (black trace) and of pure **3** (red trace).

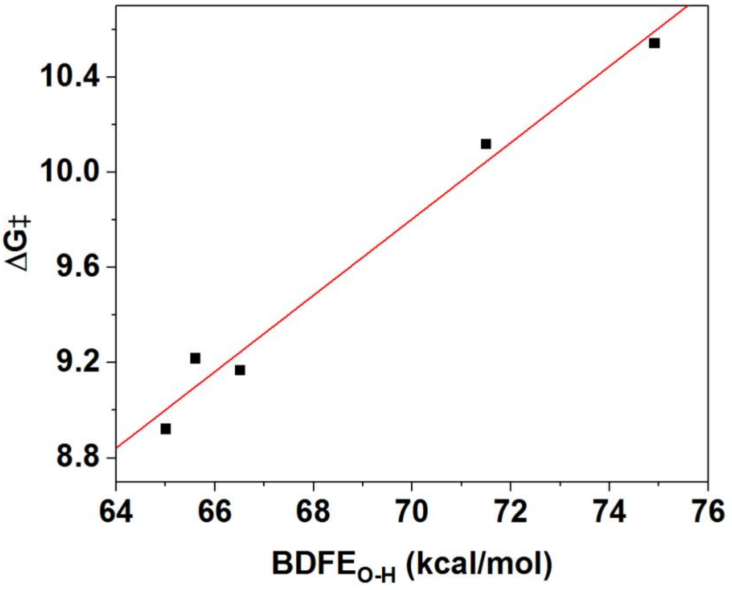

Figure S36. Plot of $\Delta G^{\ddagger}$ versus the $BDFE_{O-H}$ values for the reactions between **3** and ABNO-H, 4-X-TEMPO-H, where X = O, H, and $OCCH_3$. Values of $\Delta G^{\ddagger}$ were calculated from second-order constants k_2 via the Eyring equation (slope = 0.16). $BDFE_{O–H}$ values of the substrates are provided are plotted in CH_3CN.

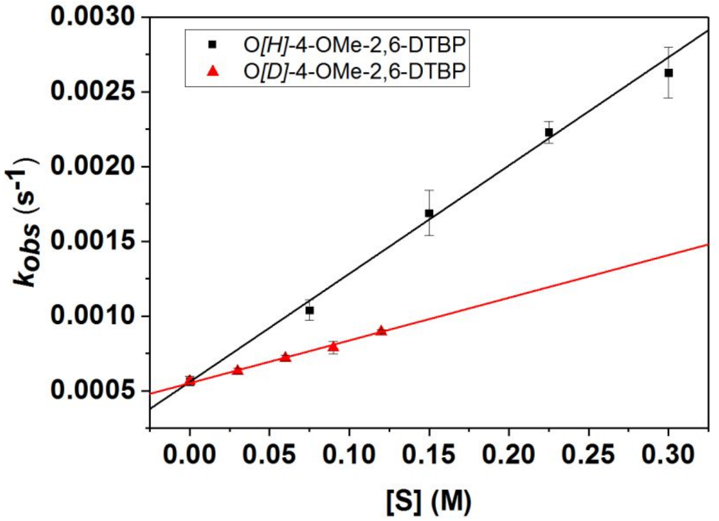

Figure S37. Plot of k_{obs} versus substrate determined for the reaction between complex **3** and *H*-4-CH_3O-2,6-DTBP (black trace) and *D*-4-CH_3O-2,6-DTBP (red trace).

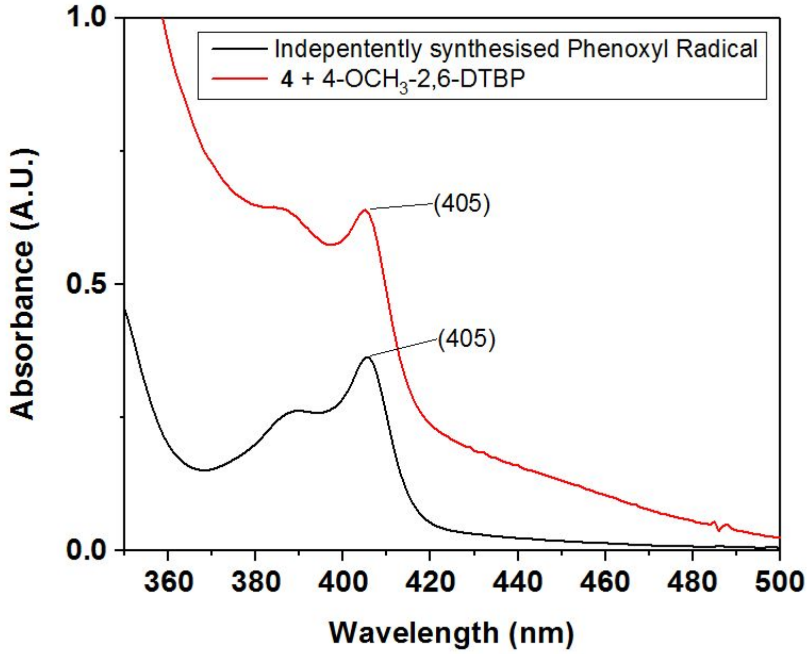

Figure S38. Electronic absorption spectra of the independently synthesised 4-methoxy-2,6-di-tert-butylphenoxyl radical (black) and after the reaction of **4** with 4-CH_3O-2,6-DTBP.

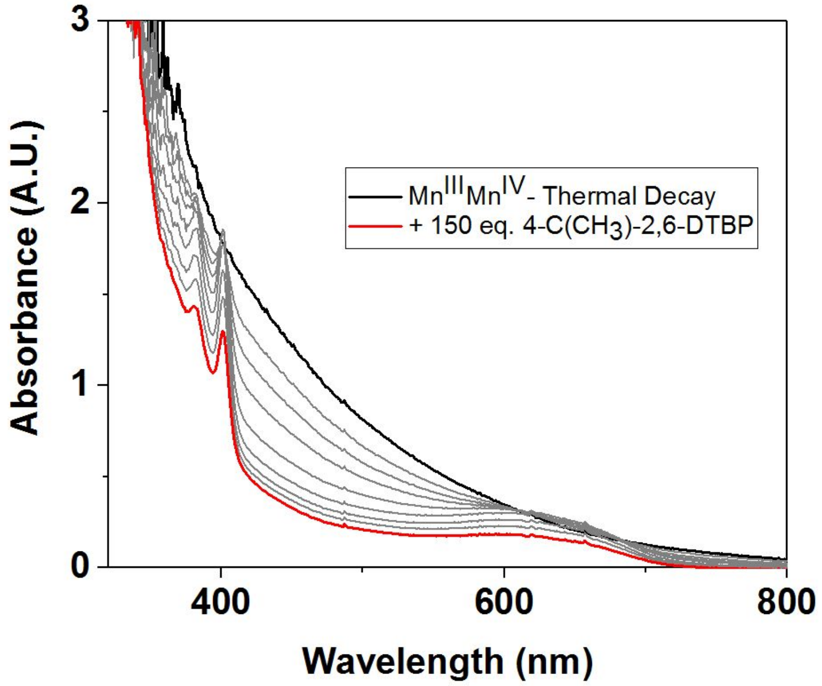

Figure S39. Electronic absorption spectra changes during the reaction of **4** (black trace) with 2,4,6-TTBP (red trace is spectrum at end of the reaction).

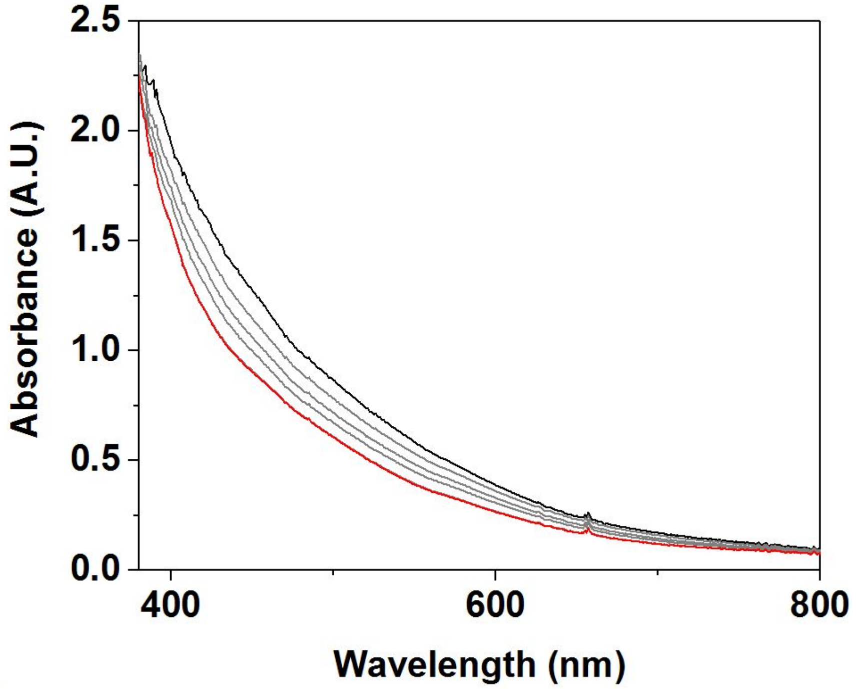

Figure S40. Electronic absorption spectra changes during the reaction of **4** (black trace) with 4-CH_3CH_2-2,6-DTBP (red trace is spectrum at end of the reaction).

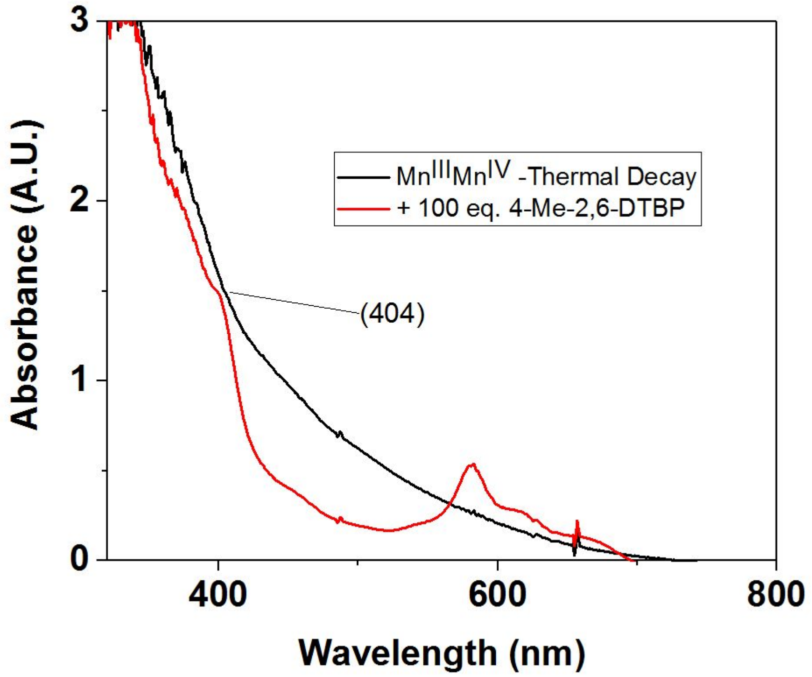

Figure S41. Electronic absorption spectra changes during the reaction of **4** (black trace) with 4-CH_3-2,6-DTBP (red trace is spectrum at end of the reaction).

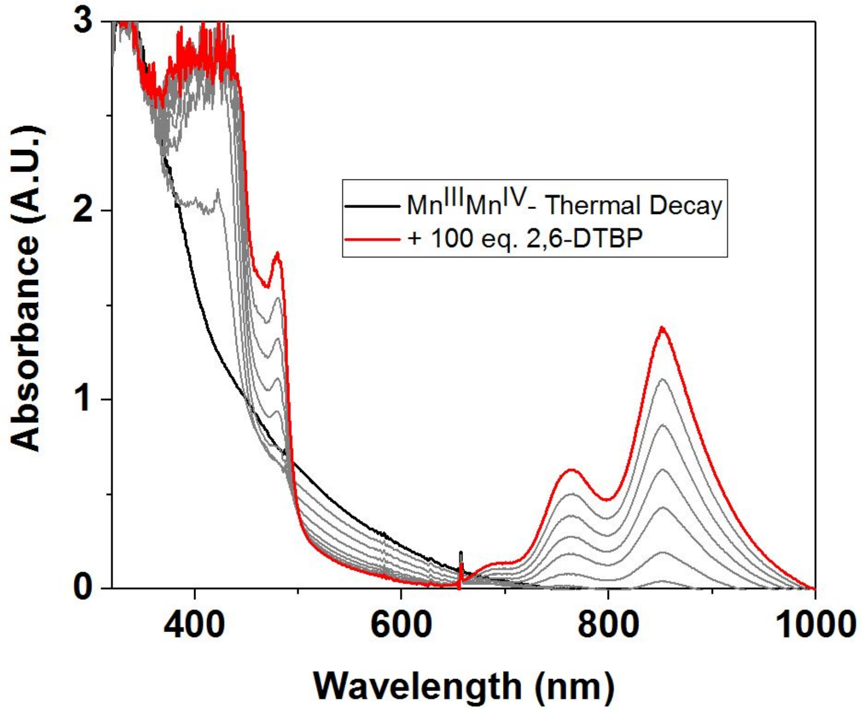

Figure S42. Electronic absorption spectra changes during the reaction of **4** (black trace) with 2,6-DTBP (red trace is spectrum at end of the reaction).

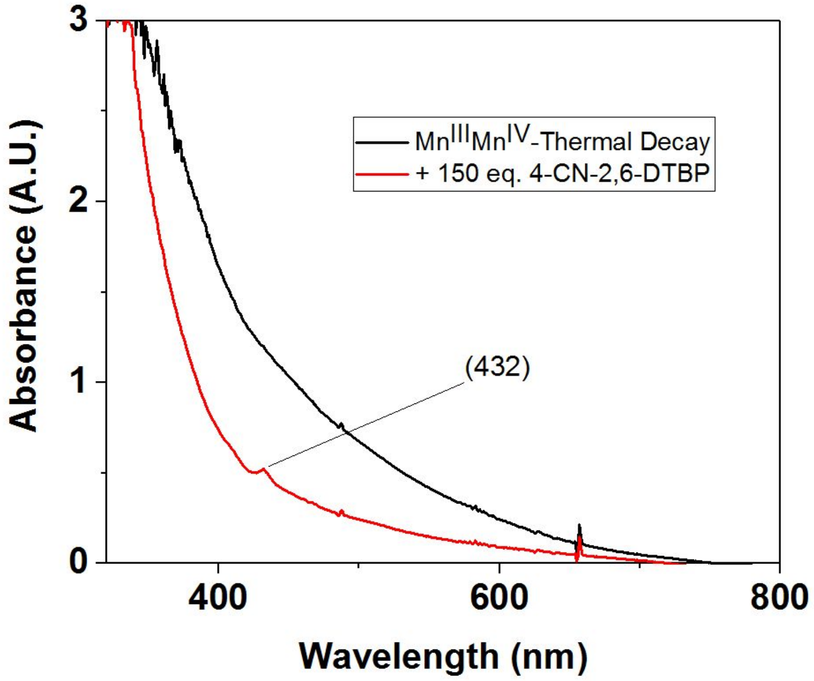

Figure S43. Electronic absorption spectra changes during the reaction of **4** (black trace) with 4-CN-2,6-DTBP (red trace is spectrum at end of the reaction).

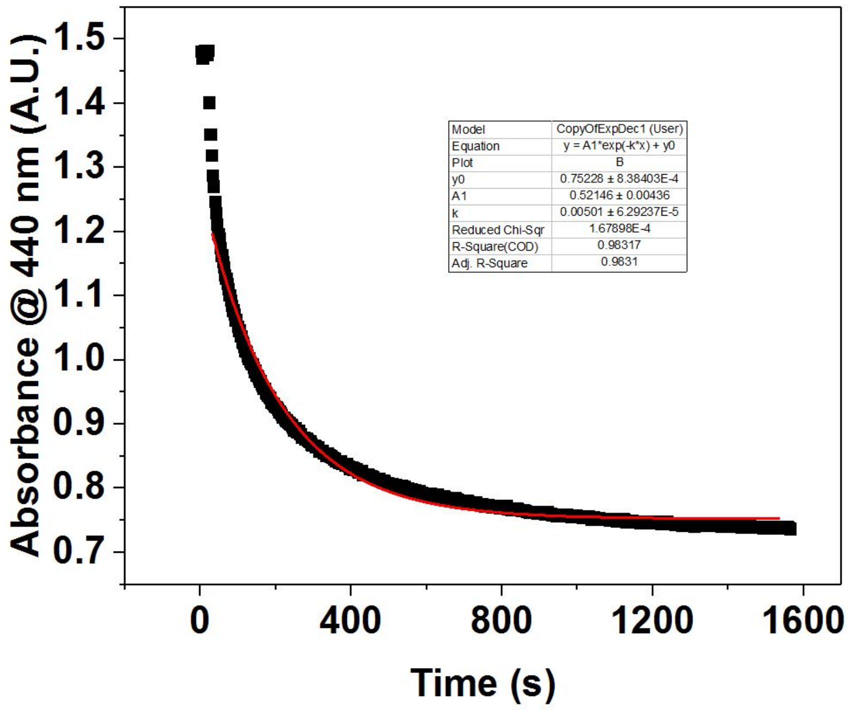

Figure S44. Plot of the absorbance at λ = 440 nm during the reaction of **4** and 2,4,6-TTBP (black trace). First-order fit of the decay (red trace).

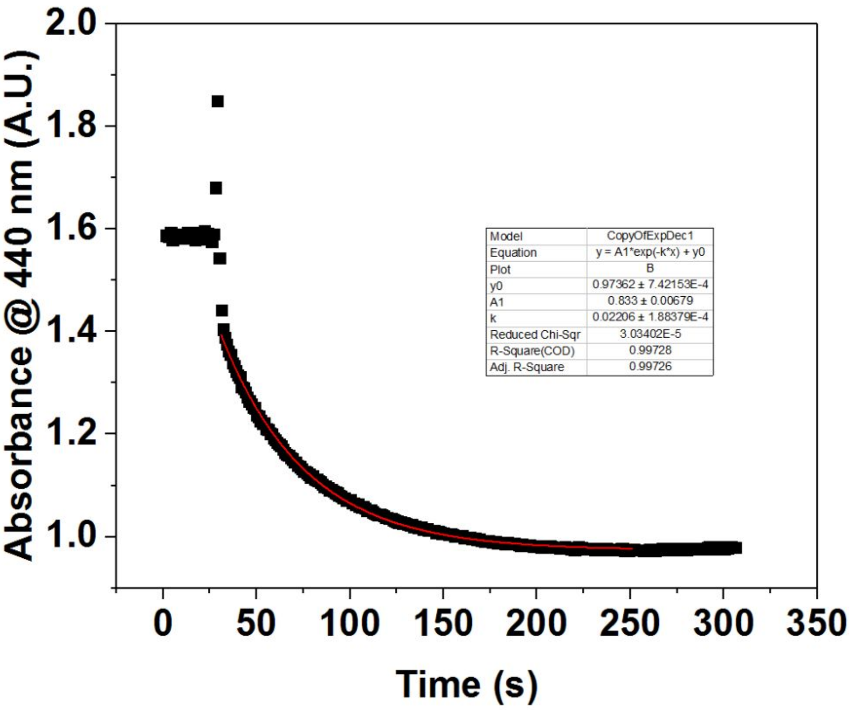

Figure S45. Plot of the absorbance at λ = 440 nm during the reaction of **4** and 4-CH_3CH_2-2,6-DTBP (black trace). First-order fit of the decay (red trace).

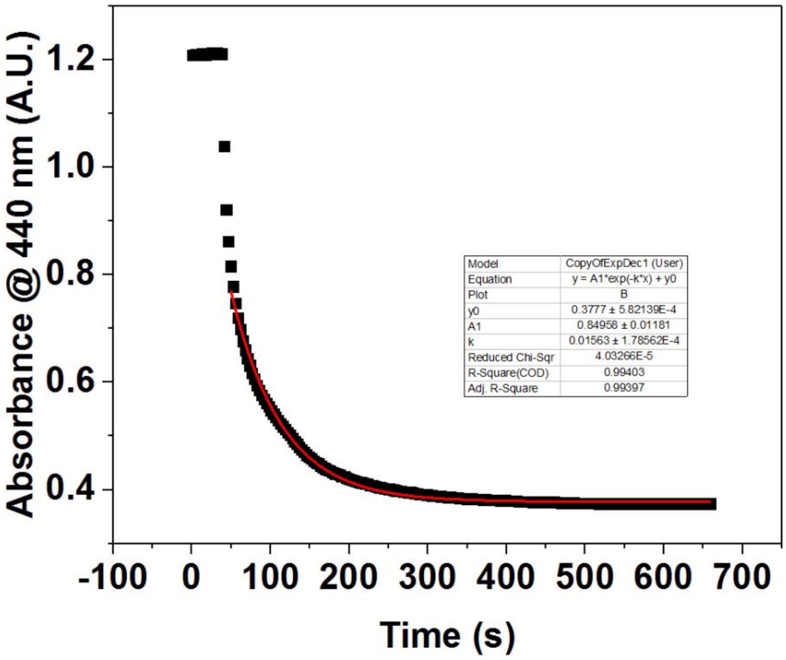

Figure S46. Plot of the absorbance at λ = 440 nm during the reaction of **4** and 4-CH_3-2,6-DTBP (black trace). First-order fit of the decay (red trace).

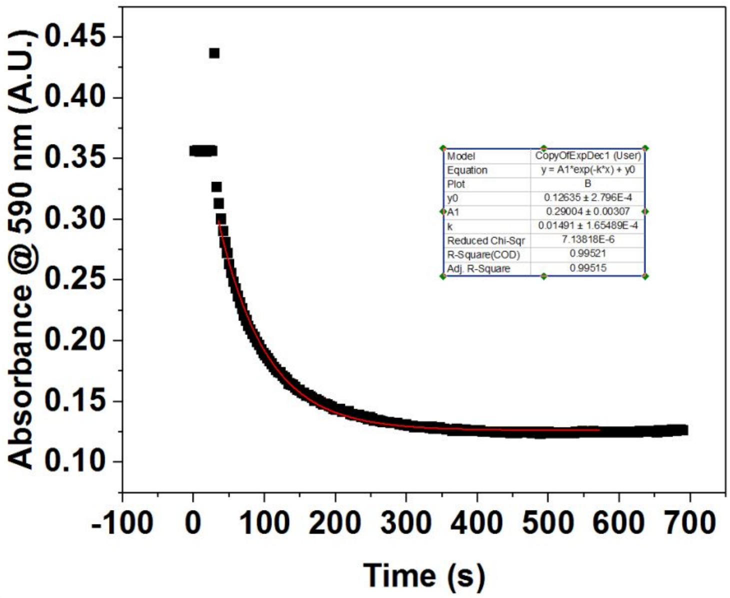

Figure S47. Plot of the absorbance at λ = 590 nm during the reaction of **4** and 2,6-DTBP (black trace). First-order fit of the decay (red trace). For 4-H-2,6-DTBP substrate, a product was formed that masked the decay of **4** at λ = 440 nm (Figure S68), hence, the decay of **4** was monitored at λ = 550 nm.

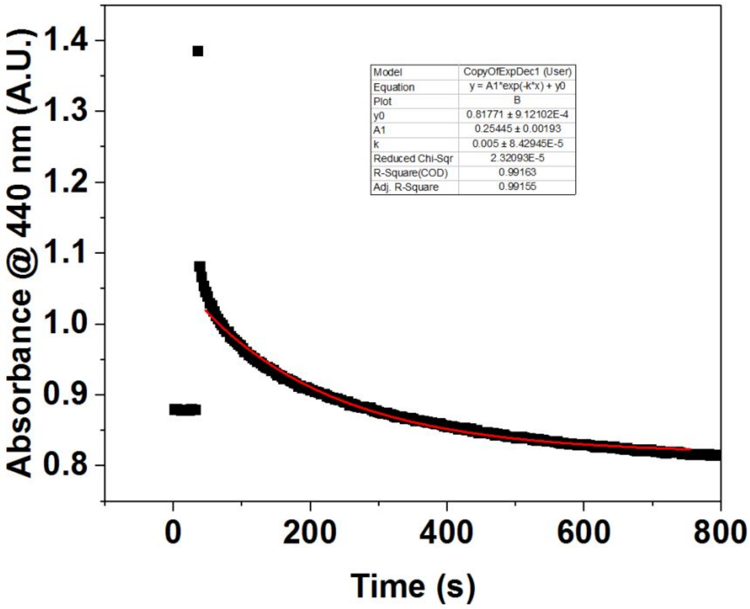

Figure S48. Plot of the absorbance at λ = 440 nm during the reaction of **4** and 4-CN-2,6-DTBP (black trace). First-order fit of the decay (red trace).

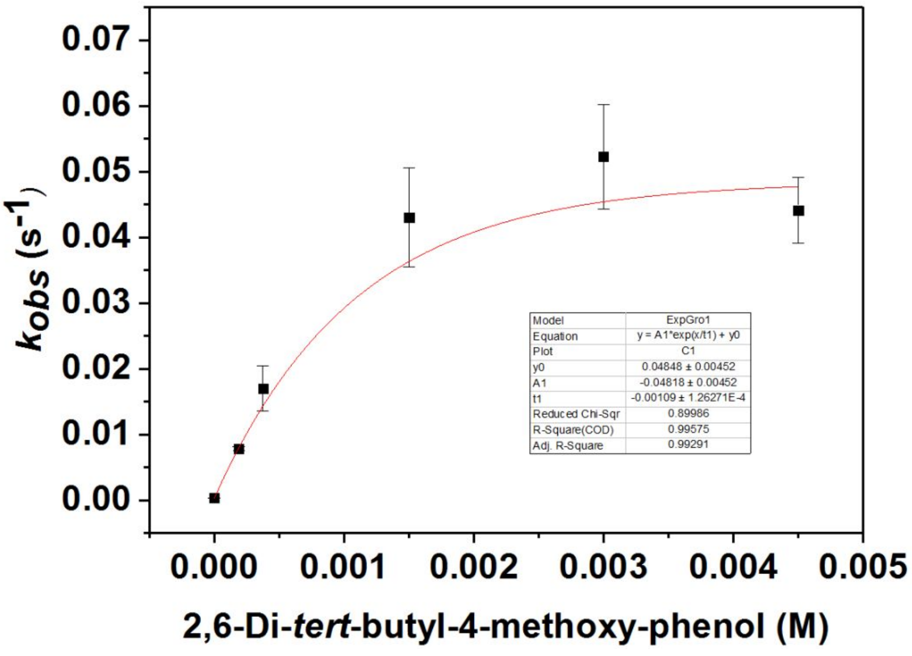

Figure S49. Plot of k_{obs} versus [4-CH_3O-2,6-DTBP] in its reaction with **4**.

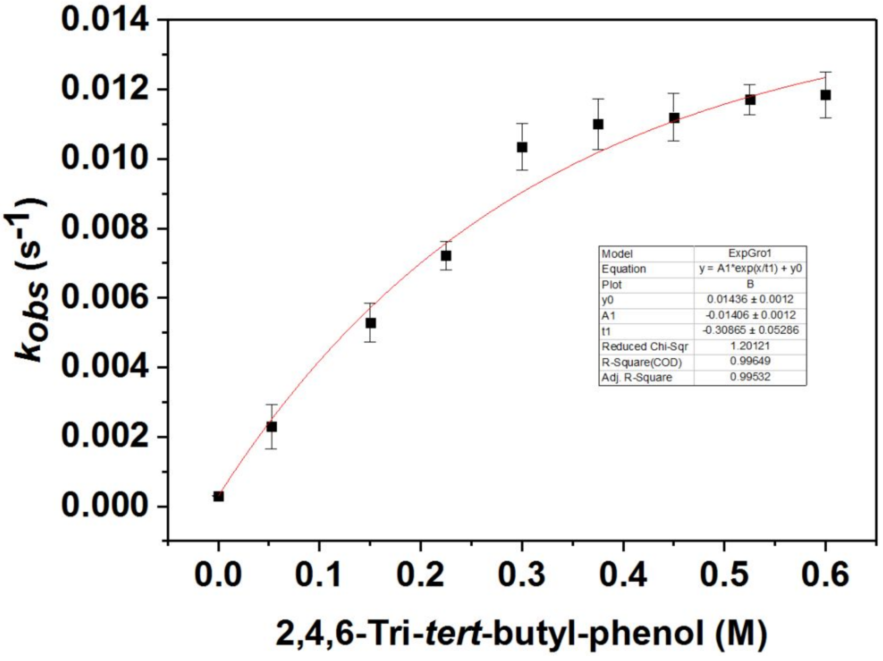

Figure S50. Plot of k_{obs} versus [2,4,6-TTBP] in its reaction with **4**.

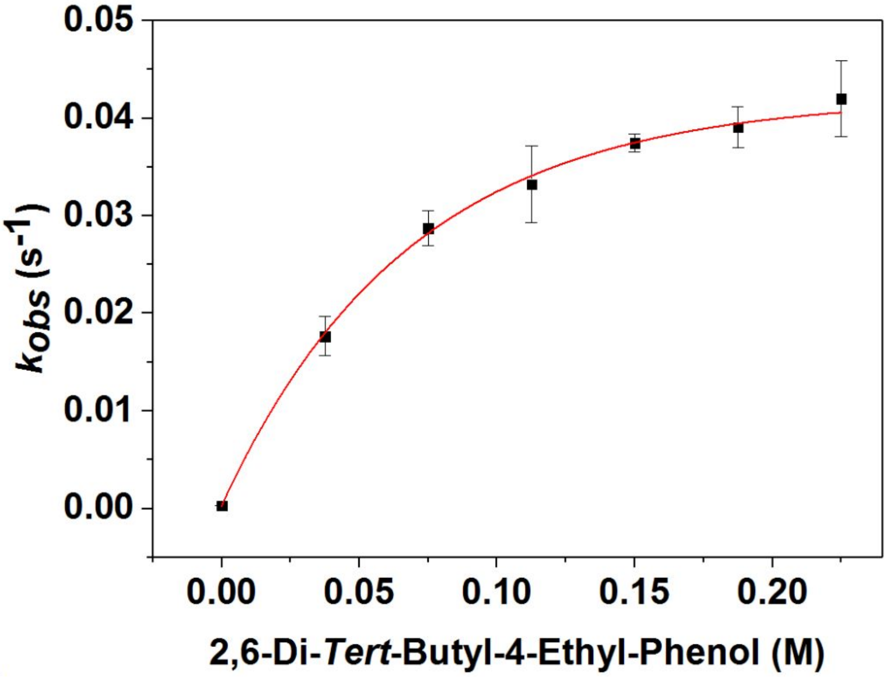

Figure S51. Plot of k_{obs} versus [4-CH_3CH_2-2,6-DTBP] in its reaction with **4**.

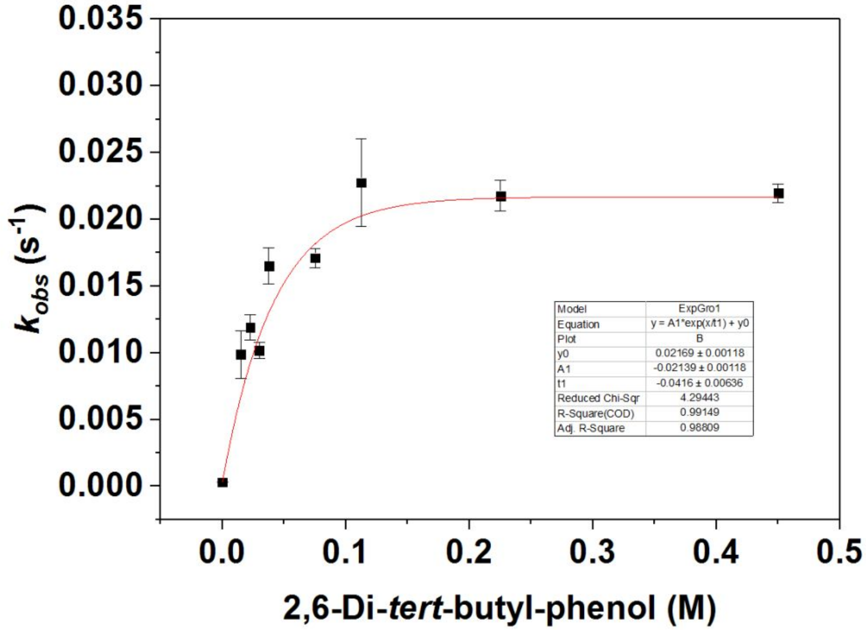

Figure S52. Plot of k_{obs} versus [2,6-DTBP] in its reaction with **4**.

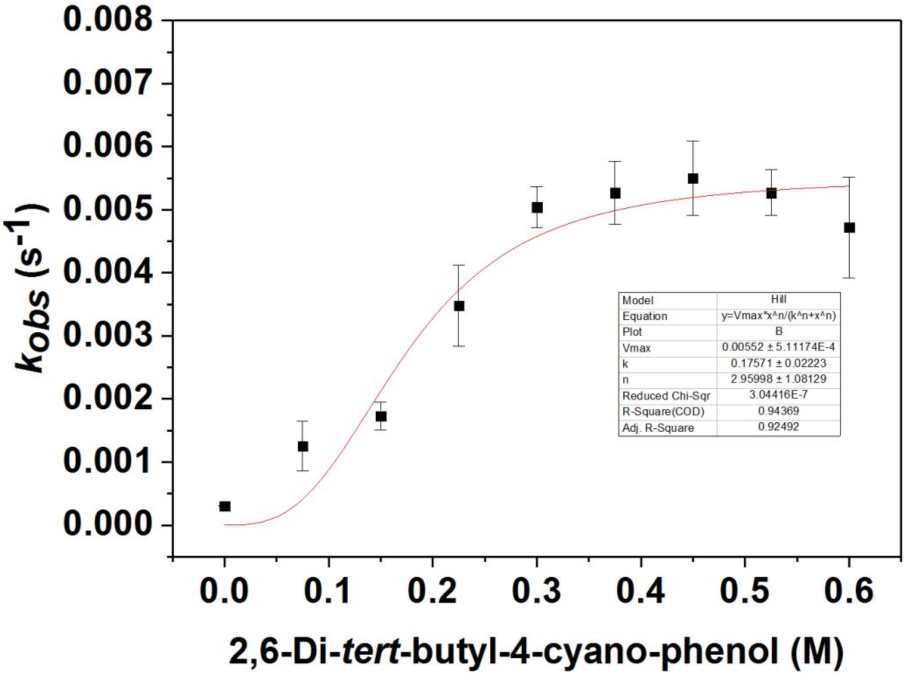

Figure S53. Plot of k_{obs} versus [4-CN-2,6-DTBP] in its reaction with **4**.

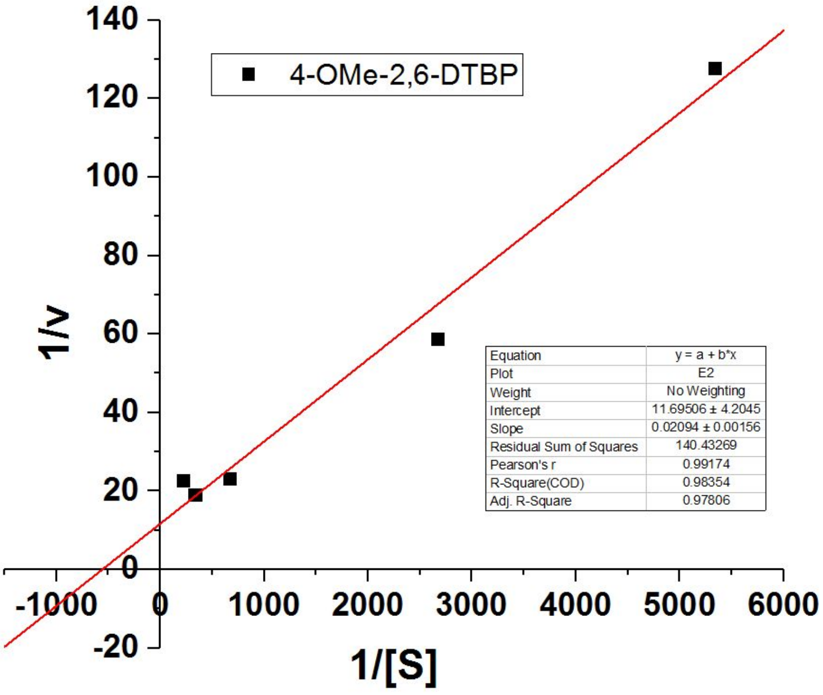

Figure S54. Plot of $1/k_{obs}$ versus 1/[4-CH_3O-2,6-DTBP] in its reaction with **4**.

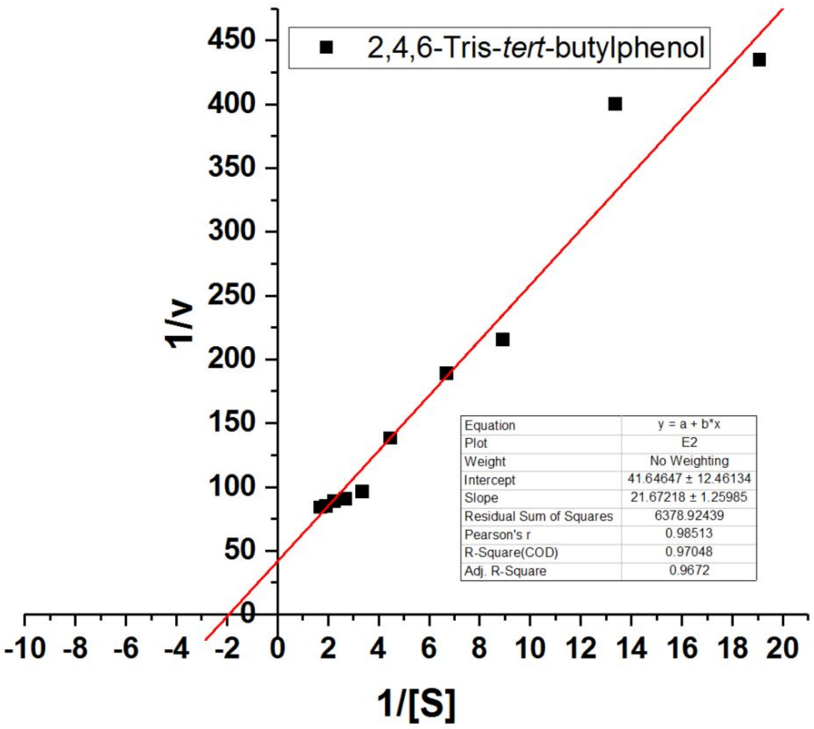

Figure S55. Plot of $1/k_{obs}$ versus 1/[2,4,6-TTBP] in its reaction with **4**.

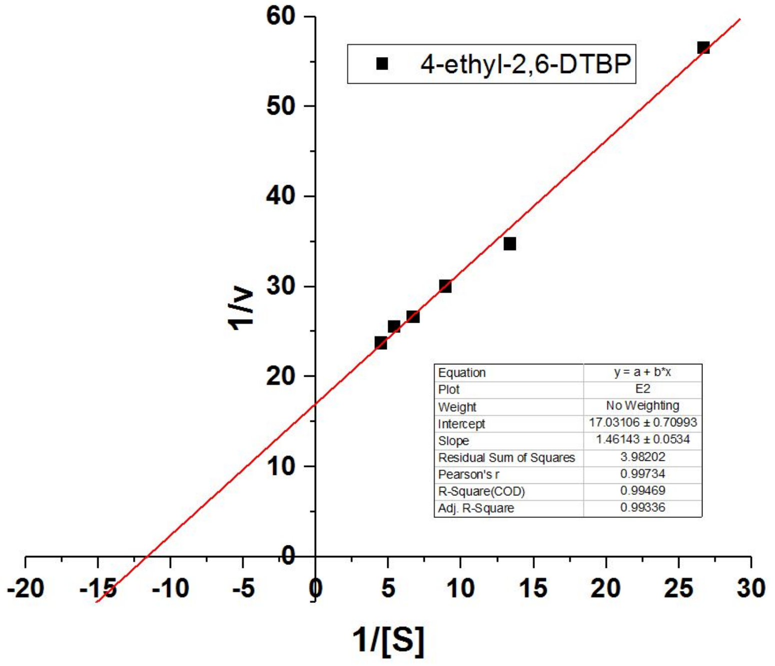

Figure S56. Plot of $1/k_{obs}$ versus 1/[4-CH_3CH_2-2,6-DTBP] in its reaction with **4**.

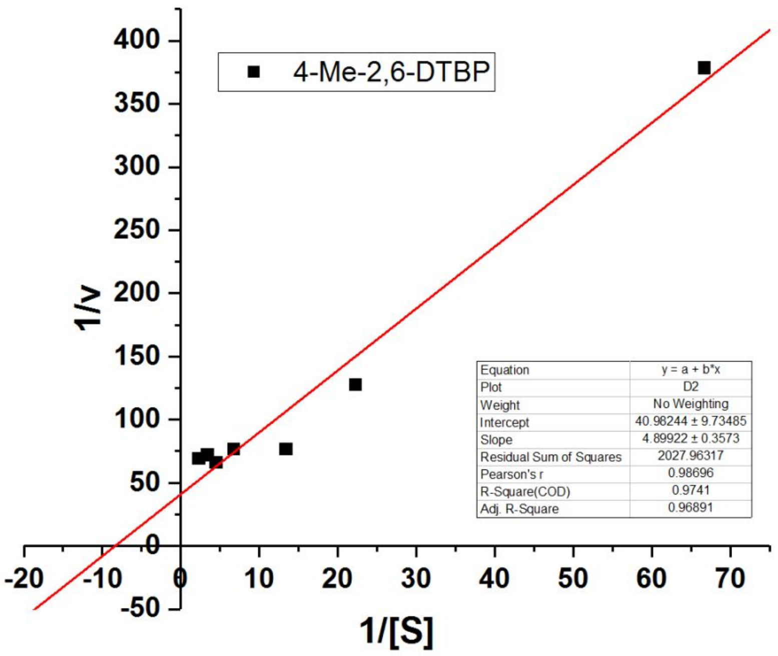

Figure S57. Plot of $1/k_{obs}$ versus 1/[4-CH_3-2,6-DTBP] in its reaction with **4**.

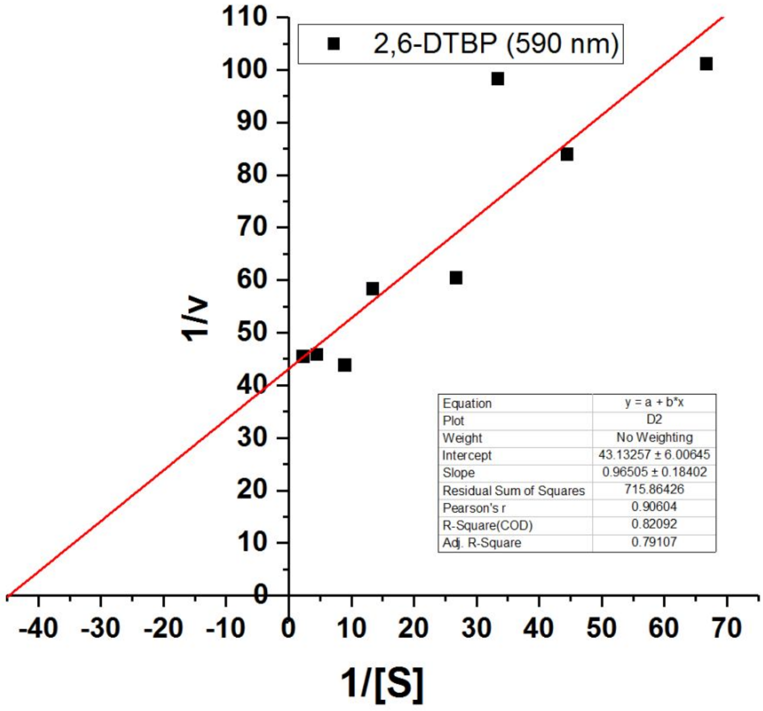

Figure S58. Plot of $1/k_{obs}$ versus 1/[2,6-DTBP] in its reaction with **4**.

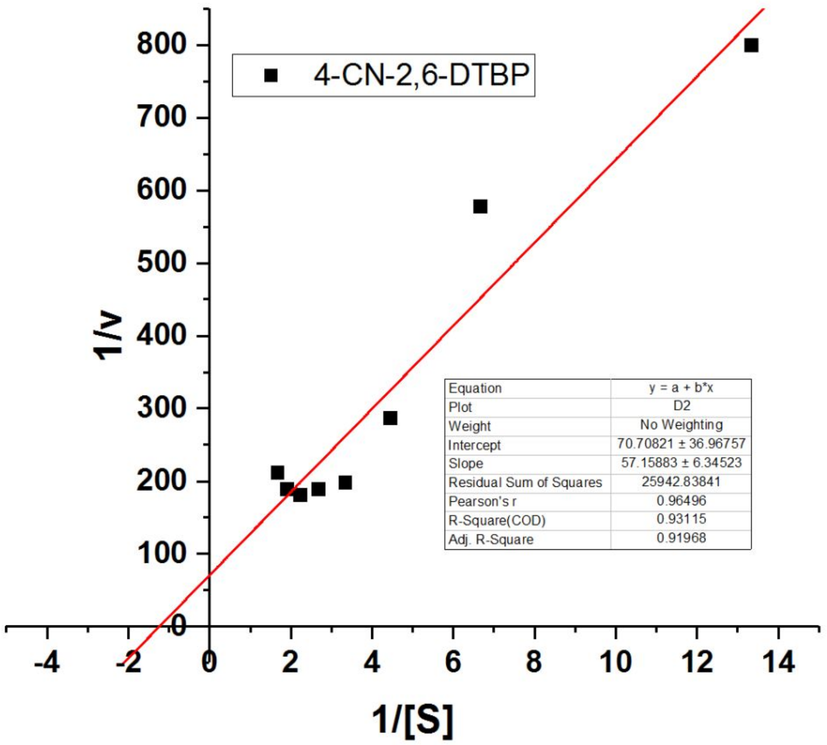

Figure S59. Plot of $1/k_{obs}$ versus 1/[4-CN-2,6-DTBP] in its reaction with **4**.

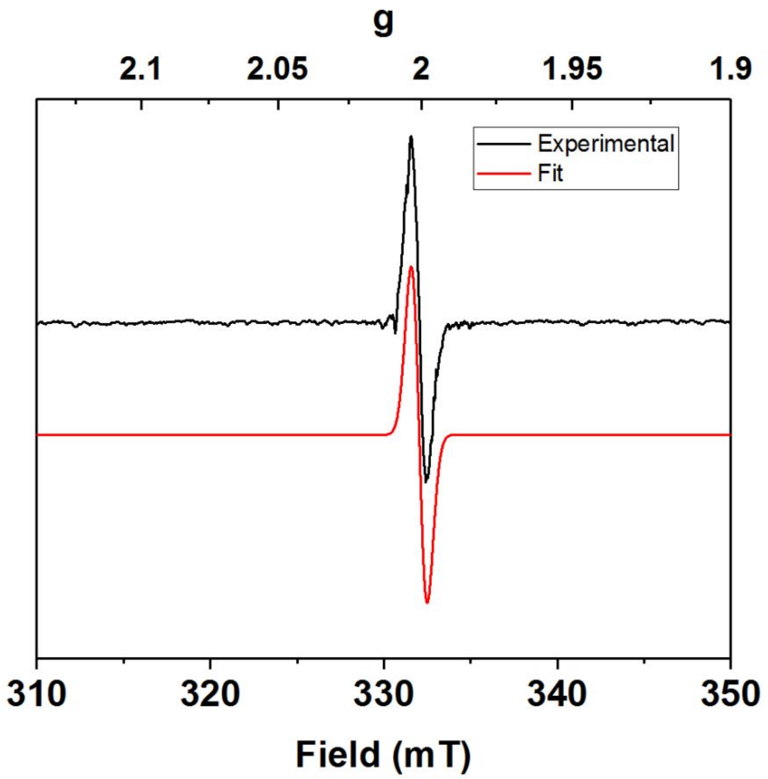

Figure S60. EPR spectrum of the post-reaction mixture from the reaction of **4** with 2,4,6-TTBP.

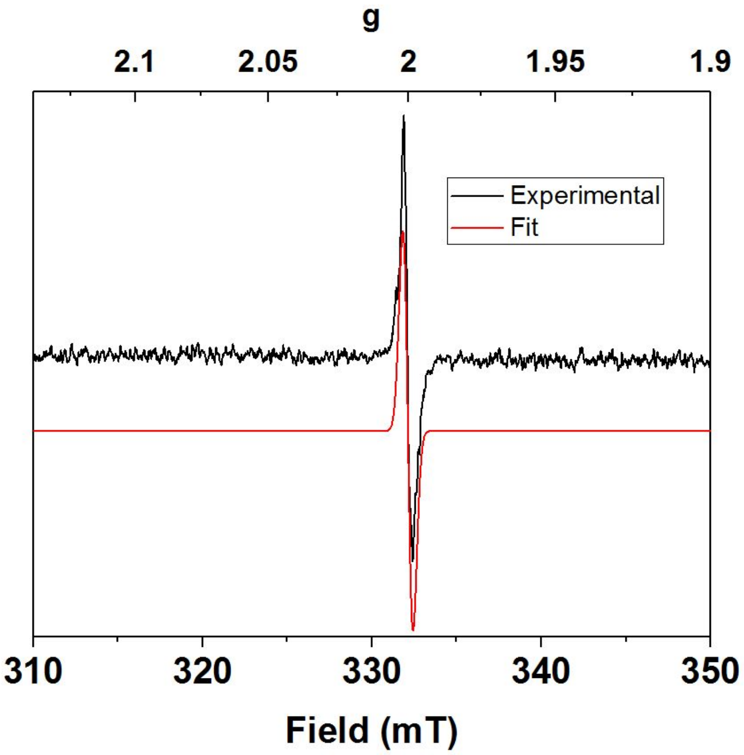

Figure S61. EPR spectrum of the post-reaction mixture from the reaction of **4** with 2,6-DTBP.

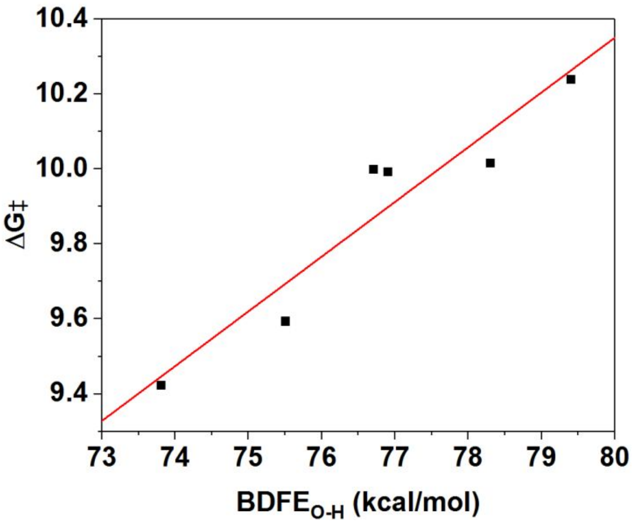

Figure S62: Plot of $\Delta G^{\ddagger}$ versus the $BDFE_{O\text{-}H}$ values for the substrates. Values of $\Delta G^{\ddagger}$ were calculated from first-order constants k_1 via the Eyring equation (slope = 0.15). The BDFE values for the substrates were not available in THF, hence, $BDFE_{O\text{-}H}$ values were plotted in benzene.

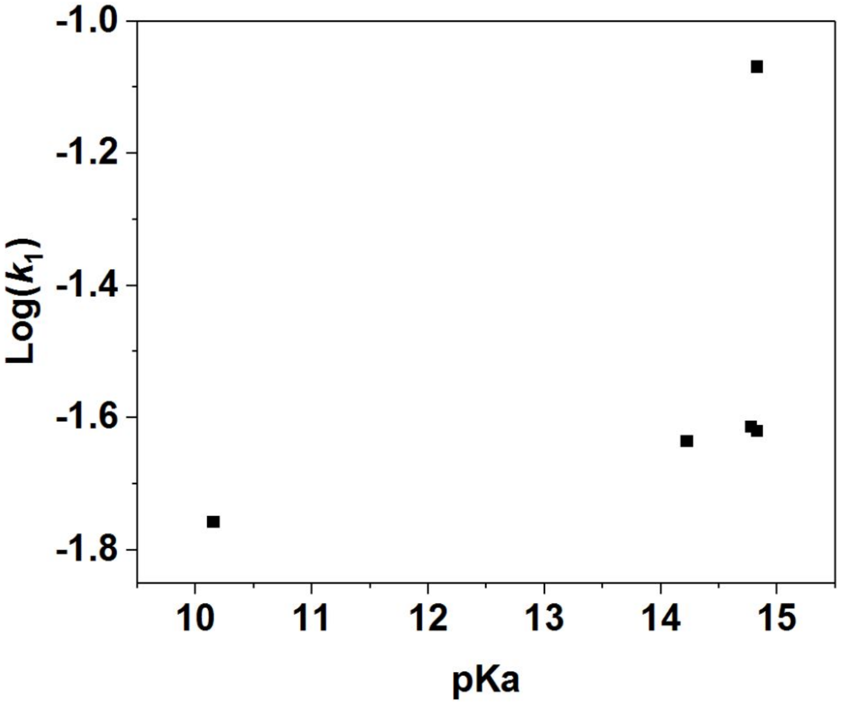

Figure S63. Plot of the log(k_1) versus pK_a of the phenol substrates for **4**.

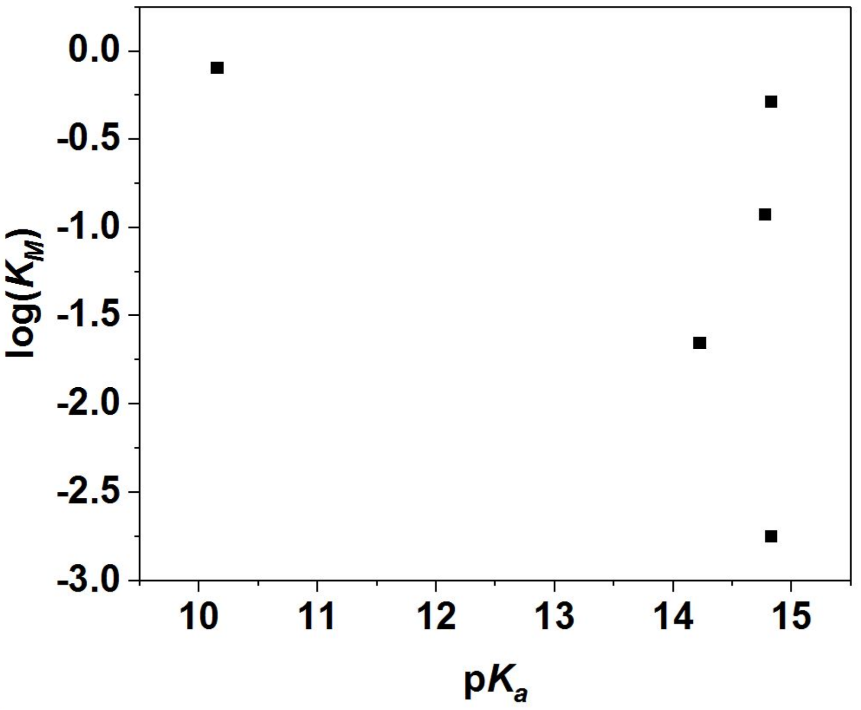

Figure S64. Plot of log(K_M) versus pK_a of the phenol substrates for **4**.

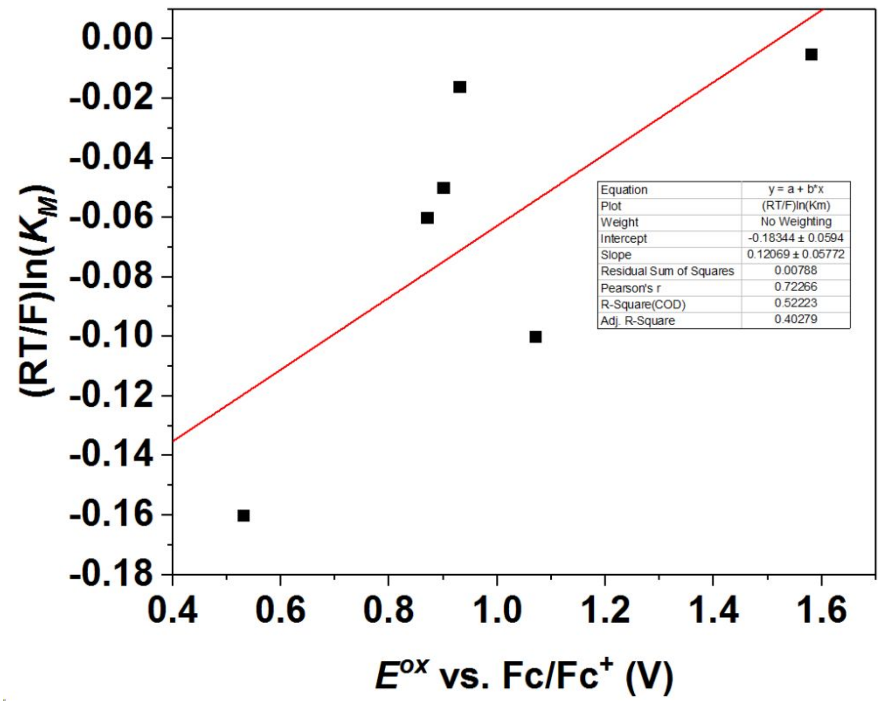

Figure S65. Plot of (RT/F)ln(K_M) versus E_{OX} for the phenol substrates for **4**.

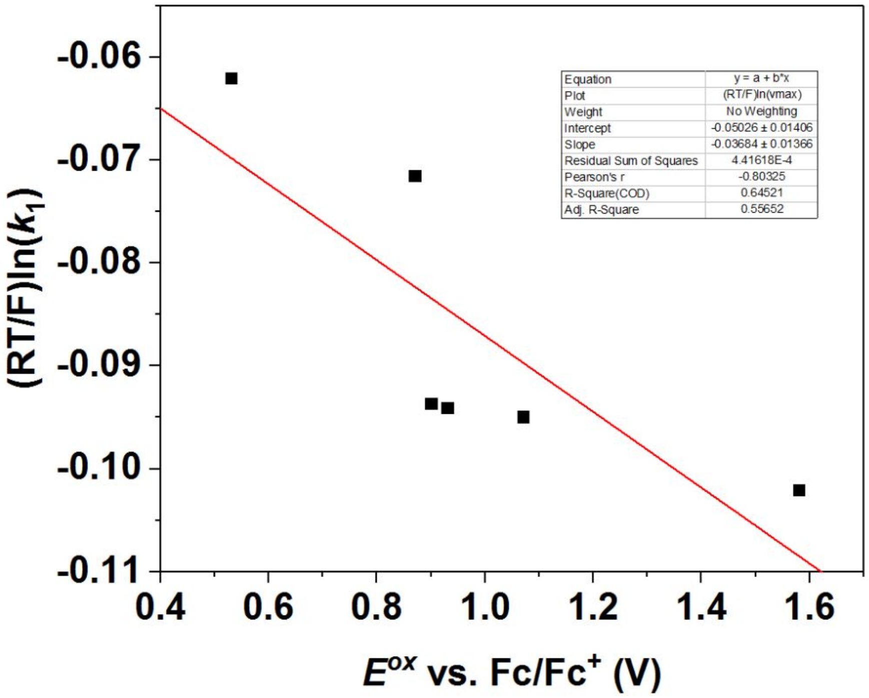

Figure S66. Plot of (RT/F)ln(k_1) versus E_{OX} for the phenol substrates for **4.**

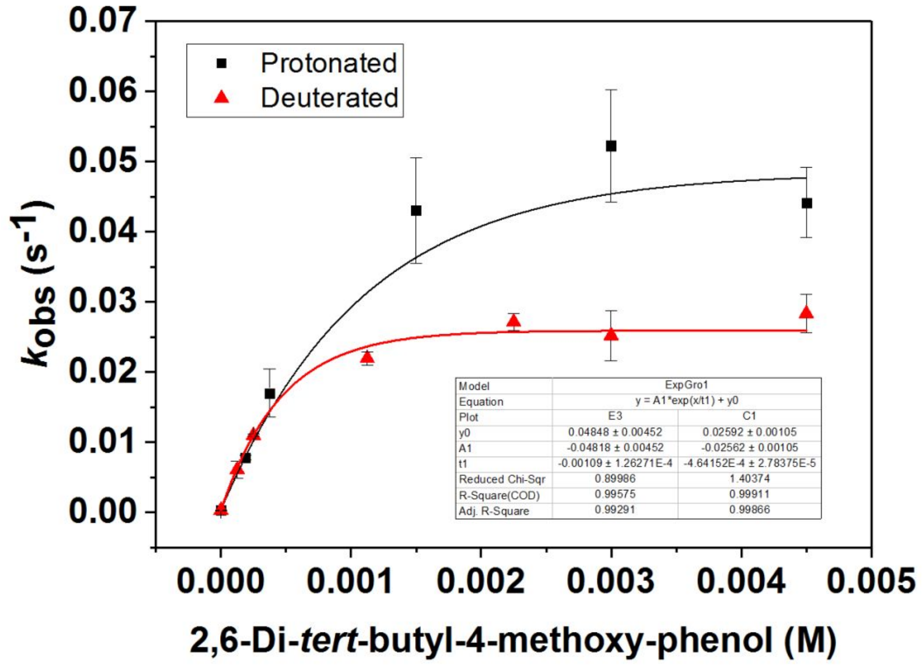

Figure S67. Plot of k_{obs} versus [*H*-4-CH_3O-2,6-DTBP] determined for the reaction of **4** and *H*-4-CH_3O-2,6-DTBP (black trace). Plot of k_{obs} versus [*D*-4-CH_3O-2,6-DTBP] determined for the reaction of **4** and *D*-4-CH_3O-2,6-DTBP (red trace).

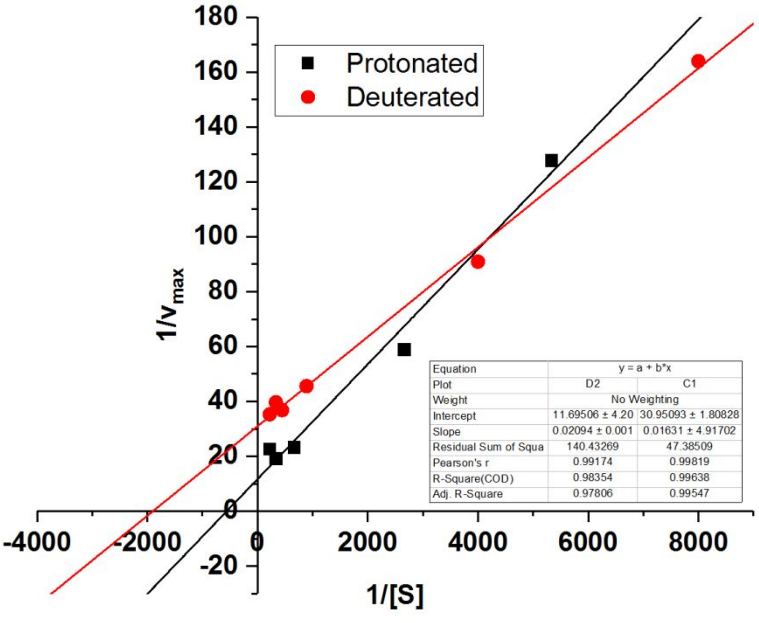

Figure S68. Plot of 1/k_{obs} versus 1/[*H*-4-CH_3O-2,6-DTBP] determined for the reaction between **4** and *H*-4-CH_3O-2,6-DTBP (black trace) and plot of 1/k_{obs} versus 1/[*D*-4-CH_3O-2,6-DTBP] determined for the reaction between **4** and *D*-4-CH_3O-2,6-DTBP (red trace).

References:

(1) Borovik, A. S.; Papaefthymiou, V.; Taylor, L. F.; Anderson, O. P.; Que, L., Jr. Models for Iron-Oxo Proteins. Structure and Properties of $Fe^{II}Fe^{III}$, $Zn^{II}Fe^{III}$, and $Fe^{II}Ga^{III}$ complexes. *J. Am. Chem. Soc.* **1989**, *111*, 6183-6195.
(2) Torelli, S.; Belle, C.; Gautier-Luneau, I.; Pierre, J. L.; Saint-Aman, E.; Latour, J. M.; Le Pape, L.; Luneau, D. pH-Controlled Change of the Metal Coordination in a Dicopper(II) Complex of the Ligand H−BPMP: Crystal Structures, Magnetic Properties, and Catecholase Activity. *Inorg. Chem.* **2000**, *39* (16), 3526-3536.
(3) Suzuki, M.; Mikuriya, M.; Murata, S.; Uehara, a.; Oshio, H.; Kida, S.; Saito, K. Syntheses and Characterization of Dinuclear Manganese(II,II) and Manganese(II,III) Complexes with Phenolate and Two Carboxylate Bridges. *Bull. Chem. Soc. Jpn.* **1987**, *60*, 4305-4312.
(4) Coggins, M. K.; Brines, L. M.; Kovacs, J. A. Synthesis and Structural Characterization of a Series of MnIIIOR Complexes, Including a Water-Soluble MnIIIOH That Promotes Aerobic Hydrogen-Atom Transfer. *Inorg. Chem.* **2013**, *52* (21), 12383-12393.
(5) Miyazawa, T.; Endo, T.; Okawara, M. New method for preparation of superoxide ion by use of amino oxide. *J. Org. Chem.* **1985**, *50* (25), 5389-5391.
(6) Stoll, S.; Schweiger, A. EasySpin, a comprehensive software package for spectral simulation and analysis in EPR. *J. Mag. Reson.* **2006**, *178*, 42-55.
(7) Ravel, B.; Newville, M. ATHENA, ARTEMIS, HEPHAESTUS: data analysis for X-ray absorption spectroscopy using IFEFFIT. *J. Synchrotron Rad.* **2005**, *12* (4), 537-541.
(8) Magherusan, A. M.; Nelis, D. N.; Twamley, B.; McDonald, A. R. Catechol oxidase activity of comparable dimanganese and dicopper complexes. *Dalton Trans.* **2018**, *47* (43), 15555-15564, Article.
(9) Magherusan, A. M.; Zhou, A.; Farquhar, E. R.; Garcia-Melchor, M.; Twamley, B.; Que, L., Jr.; McDonald, A. R. Mimicking Class Ib Mn2-Ribonucleotide Reductase: A MnII2 Complex and Its Reaction with Superoxide. *Angew. Chem. Int. Ed.* **2018**, *57* (4), 918-922.
(10) Magherusan, A. M.; Kal, S.; Nelis, D. N.; Doyle, L. M.; Farquhar, E. R.; Que, L.; McDonald, A. R. A Mn II Mn III -Peroxide Complex Capable of Aldehyde Deformylation. *Angew. Chem. Int. Ed.* **2019**, *58* (17), 5718-5722.
(11) Porter, T. R.; Capitao, D.; Kaminsky, W.; Qian, Z.; Mayer, J. M. Synthesis, Radical Reactivity, and Thermochemistry of Monomeric Cu(II) Alkoxide Complexes Relevant to Cu/Radical Alcohol Oxidation Catalysis. *Inorg. Chem.* **2016**, *55* (11), 5467-5475.
(12) Nelsen, S. F.; Kessel, C. R.; Brien, D. J. Bredt's rule kinetically stabilized nitrogen-centered radical cations and radicals in the 9-azabicyclo[3.3.1]nonyl system. *J. Am. Chem. Soc.* **1980**, *102* (2), 702-711.
(13) Warren, J. J.; Tronic, T. A.; Mayer, J. M. Thermochemistry of Proton-Coupled Electron Transfer Reagents and its Implications. *Chem. Rev.* **2010**, *110* (12), 6961-7001.
(14) Agarwal, R. G.; Coste, S. C.; Groff, B. D.; Heuer, A. M.; Noh, H.; Parada, G. A.; Wise, C. F.; Nichols, E. M.; Warren, J. J.; Mayer, J. M. Free Energies of Proton-Coupled Electron Transfer Reagents and Their Applications. *Chem. Rev.* **2022**, *122* (1), 1-49.
